# Single-cell multi-omics reveals dyssynchrony of the innate and adaptive immune system in progressive COVID-19

**DOI:** 10.1038/s41467-021-27716-4

**Published:** 2022-01-21

**Authors:** Avraham Unterman, Tomokazu S. Sumida, Nima Nouri, Xiting Yan, Amy Y. Zhao, Victor Gasque, Jonas C. Schupp, Hiromitsu Asashima, Yunqing Liu, Carlos Cosme, Wenxuan Deng, Ming Chen, Micha Sam Brickman Raredon, Kenneth B. Hoehn, Guilin Wang, Zuoheng Wang, Giuseppe DeIuliis, Neal G. Ravindra, Ningshan Li, Christopher Castaldi, Patrick Wong, John Fournier, Santos Bermejo, Lokesh Sharma, Arnau Casanovas-Massana, Chantal B. F. Vogels, Anne L. Wyllie, Nathan D. Grubaugh, Anthony Melillo, Hailong Meng, Yan Stein, Maksym Minasyan, Subhasis Mohanty, William E. Ruff, Inessa Cohen, Khadir Raddassi, The Yale IMPACT Research Team, Allison Nelson, Denise Shepard, Michael Rainone, Xiaohua Peng, Laura E. Niklason, Albert I. Ko, Ruth R. Montgomery, Shelli F. Farhadian, Akiko Iwasaki, Albert C. Shaw, David van Dijk, Hongyu Zhao, Steven H. Kleinstein, David A. Hafler, Naftali Kaminski, Charles S. Dela Cruz

**Affiliations:** 1 Section of Pulmonary, Critical Care and Sleep Medicine, Department of Internal Medicine, School of Medicine, Yale University, New Haven, CT USA; 2 Pulmonary Institute, Tel Aviv Sourasky Medical Center, Tel Aviv University, Tel Aviv, Israel; 3 Department of Neurology, School of Medicine, Yale University, New Haven, CT USA; 4 Department of Immunobiology, School of Medicine, Yale University, New Haven, CT USA; 5 Department of Pathology, Yale School of Medicine, New Haven, CT USA; 6 Center for Medical Informatics, Yale School of Medicine, New Haven, CT USA; 7 The Jackson Laboratory for Genomic Medicine, Farmington, CT USA; 8 Department of Biostatistics, Yale School of Public Health, Yale University, New Haven, CT USA; 9 Department of Genetics, Yale School of Medicine, New Haven, CT USA; 10 Department of Internal Medicine, Yale School of Medicine, New Haven, CT USA; 11 Department of Computer Science, Yale University, New Haven, CT USA; 12 Cardiovascular Research Center, Section of Cardiovascular Medicine, Department of Internal Medicine, Yale School of Medicine, New Haven, CT USA; 13 Department of Respiratory Medicine, Hannover Medical School and Biomedical Research in End-stage and Obstructive Lung Disease Hannover, German Lung Research Center (DZL), Hannover, Germany; 14 Department of Biomedical Engineering, Yale University, New Haven, CT USA; 15 Medical Scientist Training Program, Yale School of Medicine, New Haven, CT USA; 16 Yale Center for Genome Analysis/Keck Biotechnology Resource Laboratory, Department of Molecular Biophysics and Biochemistry, Yale School of Medicine, New Haven, CT USA; 17 SJTU-Yale Joint Center for Biostatistics and Data Science, Department of Bioinformatics and Biostatistics, School of Life Sciences and Biotechnology, Shanghai Jiao Tong University, Shanghai, China; 18 Yale Center for Genome Analysis, Yale School of Medicine, New Haven, CT USA; 19 School of Medicine, Yale University, New Haven, CT USA; 20 Department of Epidemiology of Microbial Diseases, Yale School of Public Health, New Haven, CT USA; 21 Section of Infectious Diseases, Department of Internal Medicine, Yale School of Medicine, Yale University, New Haven, CT USA; 22 Departments of Anesthesiology & Biomedical Engineering, Yale University, New Haven, CT USA; 23 Howard Hughes Medical Institute, Chevy Chase, MD USA; 24 Inter-Departmental Program in Computational Biology and Bioinformatics, Yale University, New Haven, CT USA; 25 West Haven Veterans Affair Medical Center, West Haven, CT USA

**Keywords:** Viral infection, SARS-CoV-2, Systems analysis, Cellular immunity

## Abstract

Dysregulated immune responses against the SARS-CoV-2 virus are instrumental in severe COVID-19. However, the immune signatures associated with immunopathology are poorly understood. Here we use multi-omics single-cell analysis to probe the dynamic immune responses in hospitalized patients with stable or progressive course of COVID-19, explore V(D)J repertoires, and assess the cellular effects of tocilizumab. Coordinated profiling of gene expression and cell lineage protein markers shows that S100A^hi^/HLA-DR^lo^ classical monocytes and activated LAG-3^hi^ T cells are hallmarks of progressive disease and highlights the abnormal MHC-II/LAG-3 interaction on myeloid and T cells, respectively. We also find skewed T cell receptor repertories in expanded effector CD8^+^ clones, unmutated IGHG^+^ B cell clones, and mutated B cell clones with stable somatic hypermutation frequency over time. In conclusion, our in-depth immune profiling reveals dyssynchrony of the innate and adaptive immune interaction in progressive COVID-19.

## Abstract (web summary)

SARS-CoV-2 infection can lead to progressive pathology in patients with COVID-19, but information for this disease progression is sparse. Here the authors use multi-omics approach to profile the immune responses of patients, assessing immune repertoire and effects of tocilizumab treatments, to find a dyssynchrony between innate and adaptive immunity in progressive COVID-19.

## Introduction

SARS-CoV-2, the virus that causes coronavirus disease 2019 (COVID-19), has caused global infection in pandemic proportions already leading to over five million deaths worldwide^1^. Infected patients can range from being asymptomatic, to having mild-moderate disease, or more severe disease requiring intensive care unit (ICU)-level care that may include mechanical ventilation and extracorporeal membrane oxygenation (ECMO)^2^. Intensive research efforts are actively ongoing to better understand the pathogenesis and treatment options of this new disease. COVID-19 associated hospitalization data have suggested severe disease disproportionately affects older individuals, those with pre-existing comorbidities, and Black and Hispanic individuals^3^.

There is accumulating evidence to suggest that dysregulated inflammation plays a significant role in the mortality and morbidity of the disease^4^. Patients with severe COVID-19 exhibit substantial immune changes including lymphopenia and increased blood levels of inflammatory biomarkers such as C-Reactive Protein (CRP), IL-1β, TNF-α, IL-8, and IL-6^4–8^. The magnitude and severity of this inflammatory response have driven attention to interventions that modulate immune responses in COVID-19 from corticosteroids to specific cytokine inhibitors^9^. The signaling pathways driven by IL-1β, TNF-α, and IL-6 have been implicated in the pathogenesis of COVID-19^10^, and antibodies against IL-6 receptor have shown early promise, including our own experience^9^; recent large-scale clinical trials have highlighted the efficacy of tocilizumab, a humanized anti-IL-6 receptor monoclonal antibody, in hospitalized COVID-19 patients^11^. In contrast to early reports emphasizing cytokine storm as a feature of COVID-19, recent studies with deeper profiling of immune cells and with larger cohorts suggest not only a hyper-activated inflammatory response, but also an aberrantly suppressed immune signature^12–16^. These seemingly conflicting results might stem from differences in disease severity and/or from cross-sectional observations at a single time-point that may vary across studies. Given that COVID-19 is an acute viral disease, it is crucial to explore changes in the immune system response across time.

Here, we employ a single-cell multi-omics approach to study the dynamics of the innate and adaptive immune system responses in COVID-19, and explore the molecular mechanisms that contribute to disease progression. Our results show a dynamic type-1 interferon response across all cell types that wanes over time with association to a decrease in viral load and is more prominent in progressive COVID-19 patients. We highlight the abnormal MHC-II/LAG-3 interaction on myeloid and T cells, respectively. TCR and BCR repertoire analysis demonstrate the altered adaptive immune response in early disease with an expansion of effector CD8^+^ T cells and unmutated plasmablasts. Lastly, we characterize the effects of tocilizumab treatment on peripheral blood immune cells. Our in-depth immune profiling reveals dyssynchrony of the innate and adaptive immune interaction in progressive COVID-19, which may contribute to delayed virus clearance.

## Results

### PBMC subtypes shift across time and disease severity in COVID-19

**Fig. 1:**
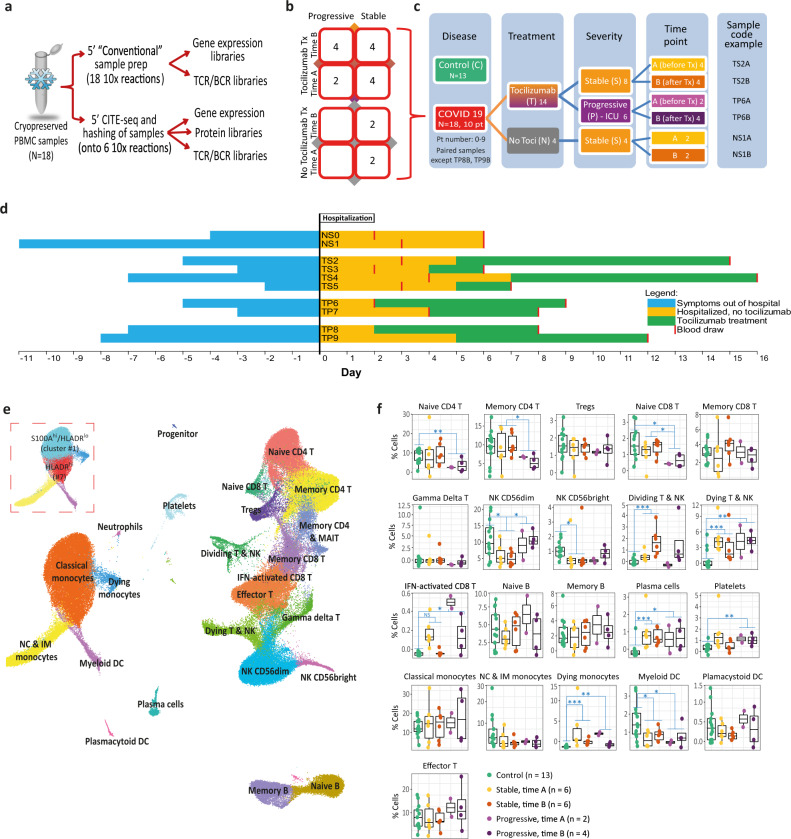
Study outline and cell clustering results. Eighteen PBMC samples from ten COVID-19 patients were included in this study, as well as 13 control samples. All COVID-19 patients had PBMC samples analyzed at two time points, except for two progressive patients who were only sampled after tocilizumab treatment. **a** Flowchart of the sample preparation methods and single-cell library types used in this study. Each COVID-19 PBMC sample was split into two after thawing and processed in parallel by two methods: conventional and CITE-seq. Control PBMC samples were only processed with the conventional sample preparation method, without CITE-seq. **b** Matrix representation of all 18 COVID-19 samples used, according to disease progression, tocilizumab treatment, and timing of blood draw. **c** A guide to patient codes and colors used throughout this manuscript. **d** A scheme depicting the timing of symptoms, hospitalization, blood draws, and tocilizumab treatment for each of the 10 COVID-19 patients. **e** UMAP embedding of single-cell transcriptomes from 153,554 cells from 18 COVID-19 and 13 control PBMC samples, annotated by cell types. Dashed box shows the two clusters of classical monocytes, HLADR^hi^ (#7) and S100A^hi^/HLADR^lo^ (#1). **f** Comparison of differential cell counts (as % of all PBMCs) between patient groups for each of the annotated cell types shown in **e**. The results are depicted in boxplots, in which the value for each sample is represented by a dot, the upper and lower bounds represent the 75% and 25% percentiles, respectively. The center bars indicate the medians, and the whiskers denote values up to 1.5 interquartile ranges above the 75% or below the 25% percentiles. The number of patients (*n*) is indicated for each group in the figure. *p-value < 0.05; **p-value < 0.01; ***p-value < 0.001, as determined by two-tailed Wilcoxon rank-sum test. DC dendritic cells, IM intermediate, IFN interferon, MAIT mucosal-associated invariant T cells, NC non-classical, NK natural killer. Source data are provided as a Source Data file.

In the current study, we sought to gain deeper insight into the immune response of COVID-19 patients across disease severities and time course of the disease. To that end, we adopted a multimodality single-cell approach to study 18 PBMC samples from 10 patients at various time-points. Age- and sex-matched healthy subjects (*n* = 13), whose samples were collected before the COVID-19 pandemic, were used as controls. Single-cell RNA-sequencing (scRNA-seq) was performed using a droplet-based single-cell platform (10x Chromium)^17^, in order to construct 5′ gene expression libraries, as well as surface protein libraries (CITE-seq)^18^, T cell receptor (TCR) libraries and B cell receptor (BCR) libraries (Fig. 1a). Following filtration and cleanup, 153,554 cells were included in the scRNA-seq analysis. In addition, we obtained clinical and laboratory information on all patients, including viral loads and cytokine panels.

Our samples were derived from both stable and progressive COVID-19 patients as part of the Yale COVID-19 IMPACT (Implementing Medical and Public Health Action Against Coronavirus CT) Biorepository. Critical patients (*n* = 4) who required treatment in the ICU and eventually succumbed to the disease were defined as having “progressive” disease, while “stable” disease defined severe patients (*n* = 6) hospitalized in internal medicine wards and eventually recovered and discharged. We analyzed PBMCs from two separate blood samples for each patient, an early (A) and a late (B) time-point, except for two progressive patients (TP8, TP9) for whom only a single sample was available (Fig. 1b–d). Eighty percent of subjects (8/10) were treated with tocilizumab according to clinical parameters, with the time-point A and time-point B samples obtained before and after the initiation of the treatment, respectively. Baseline characteristics (Supplementary Table 1), including age and sex, were similar for both control and COVID-19 patients, while individuals of European ancestry were more prevalent in the controls. Progressive patients did not differ from the stable group with regard to baseline characteristics, comorbidities, and timelines (Fig. 1d and Supplementary Table 1). The progressive patients had significantly higher modified-SOFA score, a prognostic severity score, at both time points (Supplementary Table 1).

SARS-CoV-2 RNA was not detected in any of our PBMC samples. In addition, we did not detect the expression of *ACE2*, the functional host receptor for SARS-CoV-2^19^, which may diminish the likelihood of PBMC infection.

Applying Louvain clustering to the filtered and integrated Seurat object, and plotting in uniform manifold approximation and projection (UMAP) space, 22 cell types were identified and manually annotated (Fig. 1e and Supplementary Fig. 1) across 30 cell clusters (Supplementary Fig. 2a), with a good overlap between different samples and subgroups (Supplementary Fig. 2b, c). Automated annotation using SingleR package^20^ (Supplementary Fig. 2d) supported the results of the manual annotation. Good overlap was also noted between cells processed with and without CITE-seq (i.e., non-CITE, Supplementary Fig. 2e), except for the dying monocytes cluster which was reduced in CITE samples, possibly due to the exclusion of these dying cells during the additional staining process. Importantly, viability was similar (approximately 85–90%) for CITE and non-CITE samples before loading the cells to the 10× Chromium Chip. A detailed comparison between the CITE and non-CITE samples (Supplementary Fig. 3) showed a high similarity of gene expression, and data sets were therefore combined for subsequent analysis.

Several differences in the relative abundance of specific cell types were detected across control, stable, and progressive samples at the two time points (Fig. 1f). Some notable statistically significant differences were a relative decrease in naive T cells (both CD4^+^ and CD8^+^) in progressive patients, as well as an increase in plasmablasts and dividing T & NK cells in COVID-19 patients vs controls. Cells belonging to the interferon (IFN)-activated CD8 T cell cluster (Fig. 1e and Supplementary Fig. 4), a small cluster of 191 cells characterized by very high expression of IFN stimulated genes (ISGs), were found almost exclusively in COVID-19 patients (*p* = 0.006), especially at time point A.

Some differences in relative cell proportions were noted for the innate immune arm as well. The classical monocytes population sub-clustered into two distinct populations (dashed box in Fig. 1e): one with low expression of HLA-DR, a major histocompatibility complex (MHC) class II molecule (cluster #1), subsequently referred to as S100A^hi^/HLA-DR^lo^ monocyte, and an HLA-DR^hi^ monocyte (cluster #7) as further discussed below. The proportion of S100A^hi^/HLA-DR^lo^ monocyte cluster was higher in COVID-19 patients compared to controls (Supplementary Fig. 5a, b), which is consistent with previous studies^15,21^. On the other hand, non-classical monocytes (and their marker FCGR3A) were decreased in COVID-19 (Supplementary Fig. 5), which is also consistent with recent studies^13,16,21^. Myeloid dendritic cells (DC) were also decreased in COVID-19 patients (Fig. 1f).

To conclude, our comprehensive atlas of PBMCs in stable and progressive COVID-19 patients at different time points, captured dynamic shifts in the relative abundance of specific cell types, reflective of the immune system response to the virus.

### Type-1 interferon signature dominates peripheral immune cells in COVID-19

**Fig. 2:**
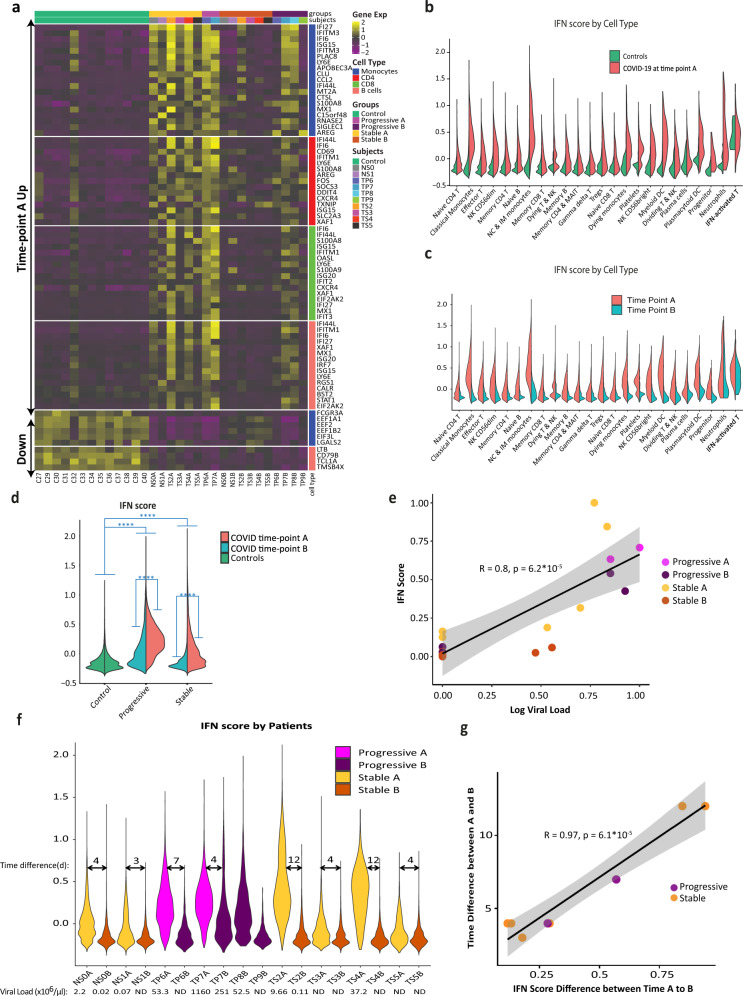
Strong interferon response is observed in COVID-19 samples. **a** Heatmap showing the top differentially expressed genes (logFC > 0.5, adjusted *p*-value < 0.05 calculated by Wilcoxon rank-sum test with Bonferroni correction for multiple comparisons) for each major cell type, comparing time-point A and B. The level of expression of these genes in control samples is shown as well. The upper part of the heatmap depicts genes that are increased at time-point A compared to B (marked “time-point A up”). **b**–**g** IFN-I scores were calculated based on the expression of 12 ISGs for each sample. **b** IFN-I score is markedly increased in all cell types in COVID-19 at time point A, relative to controls. **c** IFN-I score decreases from time-point A to B in nearly all cell types**. d** IFN-I score is higher in progressive vs stable COVID-19 patients, and at time-point, A (earlier blood draw) compared to time-point B (later one). ****p-value < 1E−300 calculated by Wilcoxon rank-sum test. **e**, **f** Viral load for each patient was calculated based on RT-qPCR analysis of nasopharyngeal swabs or saliva samples. **e** Shown is a scatter plot of scaled log viral load vs scaled IFN-I score for all COVID-19 samples. Correlation coefficient (*R*) and *p*-value are indicated. Error bands denote a 95% confidence interval. *p*-value was calculated based on an *F*-test for the significance of the regression model. **f** Shown is a violin plot depicting the IFN-I score for each sample, with the corresponding viral loads indicated below the plot. Arrows mark the time difference (in days) between paired samples (i.e., from the same patient) at two time-points: A (early/before tocilizumab treatment) and B (late/after tocilizumab). **g** Scatter plot for the 8 paired samples, showing a very high correlation between the time difference from sample A to B and the respective change in scaled IFN-I score during that time. Correlation coefficient (*R*) and *p*-value are indicated. Error bands denote a 95% confidence interval. *p*-value was calculated based on an *F*-test for the significance of the regression model. IFN interferon, ND not detectable. FC fold-change. Source data are provided as a Source Data file.

We further analyzed gene expression changes in each cell type as well as alterations over the time course of the disease. We observed that type-1 interferon (IFN-I) response was elevated in COVID-19 across all cell types, especially at time-point A (Fig. 2a, c), and more so in progressive subjects (Fig. 2d). As expected, there was a strong correlation between the IFN-I score and the concurrent viral load at the sample level (*R* = 0.8; Fig. 2e, f). Conventional ISGs, such as *IFI6, IFI44L, LY6E*, and *ISG15*, were markedly increased in COVID-19 patients compared to healthy controls across all major cell types in PBMCs (Fig. 2a). These results are in agreement with recently published studies^22,23^, which identified a strong IFN-I response in various subpopulations of PBMCs derived from COVID-19 patients. Amphiregulin (AREG), a ligand for epidermal growth factor receptor (EGFR) not known as a major ISG in humans, is barely detectable in healthy control PBMCs but is significantly increased in COVID-19 patients’ monocytes, T cells, NK cells, and DCs (Supplementary Fig. 6). Although AREG is known to play important roles in wound repair and resolution of inflammation^24^, its expression has also been reported to be increased in viral infections of the lung^25^ and induce severe lung pathology in a mouse model of SARS-CoV infection^26^. IFN-I signaling plays an important role in AREG induction within myeloid cells in mice^27^. A recent report using bulk RNA-sequencing showed an increase of *AREG* in the PBMCs of COVID-19 patients^28^, supporting a potential role of AREG in SARS-CoV-2-induced lung pathology.

### IFN-I response decreases over time in correlation with virus clearance

The time course of COVID-19 disease is characterized by shifts in many genes (Fig. 2a) and ligand–receptor interactions (Supplementary Fig. 7)^29^. As expected, the IFN-I score markedly decreases over time from time-point A (earlier blood draw) to B (later one) in all patients and all cell types, corresponding to a decrease in viral loads between those time-points (Fig. 2f). Notably, the decrease in IFN-I score between time-point A and B correlates strongly with the time difference between them (*R* = 0.97, Fig. 2g). Symptom onset is reported to occur at a median of 5.2 days after infection^7^, and since blood draw A was taken at least 5 days after symptom onset (Fig. 1d), at this time-point our patients would be expected to be on the descending slope of the viral load curve^30^. This is consistent with our observations of a uniform decrease in viral load and IFN-I score between the two time points. However, in two out of four progressive patients (and none of the six stable ones), both IFN-I score and viral load remained relatively high at time-point B. The gene expression signature of these two patients at time-point B (TP7B, TP8B) resembles the signature of other patients at the earlier time point A, while the other patients at time point B are closer to the healthy controls’ gene expression signature (Fig. 2a). This observation is consistent with a recent publication^4^, suggesting that some progressive patients are slower in clearing the virus, possibly due to immunosuppressive mechanisms discussed in the following sections. Altogether, these findings suggest that in most patients, the initially elevated IFN-I response decreases over time together with the decrease in viral loads. Interestingly, in some progressive patients, the IFN-I response seems to persist, concordantly with decreased viral clearance.

### Marked gene expression changes differentiate progressive from stable patients

**Fig. 3:**
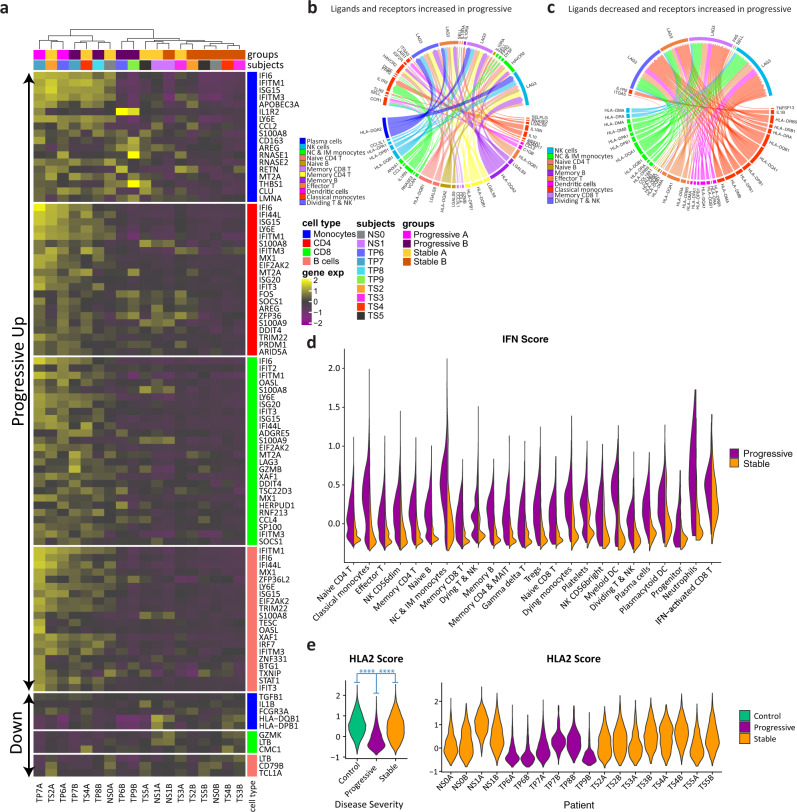
Severe COVID-19 is associated with marked changes in gene expression and connectome. **a** Heatmap showing the top differentially expressed genes (logFC > 0.5, adjusted *p*-values < 0.05 calculated by Wilcoxon rank-sum test with Bonferroni correction for multiple comparisons) for each major cell type, comparing progressive to stable patients. The upper part of the heatmap depicts genes that are increased in progressive compared to stable patients (marked “progressive up”). Hierarchical clustering separates most of the progressive samples from the stable ones based on gene expression similarities (except for some stable samples at time point A, which cluster with the progressive ones). **b**, **c** Differential connectivity maps (connectomes) of ligands (bottom half) and receptors (upper half). For each cell type, log-fold changes of ligands and receptors were calculated, comparing progressive to stable COVID-19 patients; we only plotted edges with >10% of cells expressing the ligand and receptor, and with an adjusted *p*-value < 0.05 for the comparison; edge size is proportional to the degree of change between progressive and stable patients. **b** Connectome of ligands and receptors that are both increased in progressive vs stable patients. **c** Connectome of ligands that are decreased and receptors that are increased in progressive vs stable patients. **d** A violin plot depicting differences in IFN-I score between progressive and stable COVID-19 patients in all PBMC subpopulations. **e** A composite score of nine HLA type 2 genes that are highly expressed in all subjects (HLA2 score) is decreased in monocytes of progressive patients relative to stable ones and controls. The right panel depicts the HLA2 scores of individual patient samples. ****p-value < 1E−300 calculated by Wilcoxon rank-sum test.

We observed marked gene expression differences between stable and progressive patients that span across all cell lineages (Fig. 3a–c and Supplementary Data 1–4). The expression of ISGs is increased in all cell types in progressive subjects (Fig. 3a, d). Interestingly, there is an increased expression of the suppressive cytokine *IL10* in myeloid cells and several additional cell types in progressive patients (Supplementary Fig. 8a). Levels of IL-10 in plasma are known to be increased in severe COVID-19, as reported in our recent study^4^ as well as by others^7,31^. IFN-I has been reported to induce IL-10 expression, thus limiting immune-related tissue damage in certain conditions^32,33^. Similar to ISGs, the level of plasma IL-10 decreases from time-point A to B (Supplementary Fig. 8b), although in a larger cohort of patients this decrease was only seen in stable non-ICU patients and the IL-10 level was kept higher in ICU patients (Supplementary Fig. 8d). We observed a modest positive correlation (*R* = 0.50, Supplementary Fig. 8c) between the IFN-I score in PBMCs and plasma IL-10 levels, which may support an association between the strength of the IFN-I response and the suppressive IL-10 response observed in COVID-19 patients.

In addition, we observed a decrease in MHC-II transcripts in antigen-presenting cells (APCs) of progressive subjects compared to stable ones, with the latter being more similar to that of control subjects (Fig. 3c, e and Supplementary Fig. 9). Increased IL-10 is known to downregulate the expression of MHC-II^34,35^, possibly explaining this observed decrease in progressive subjects.

Together, this suppressive signature of increased IL-10 and decreased MHC-II in progressive patients might serve as a double-edged sword: on the one hand, decreasing inflammation and protecting tissues from immune-related damage, and on the other hand hampering the ability to mount an effective antiviral response, as will be further discussed in the next section.

### Progressive patients exhibit S100A^hi^/HLA-DR^lo^ myeloid phenotype

**Fig. 4:**
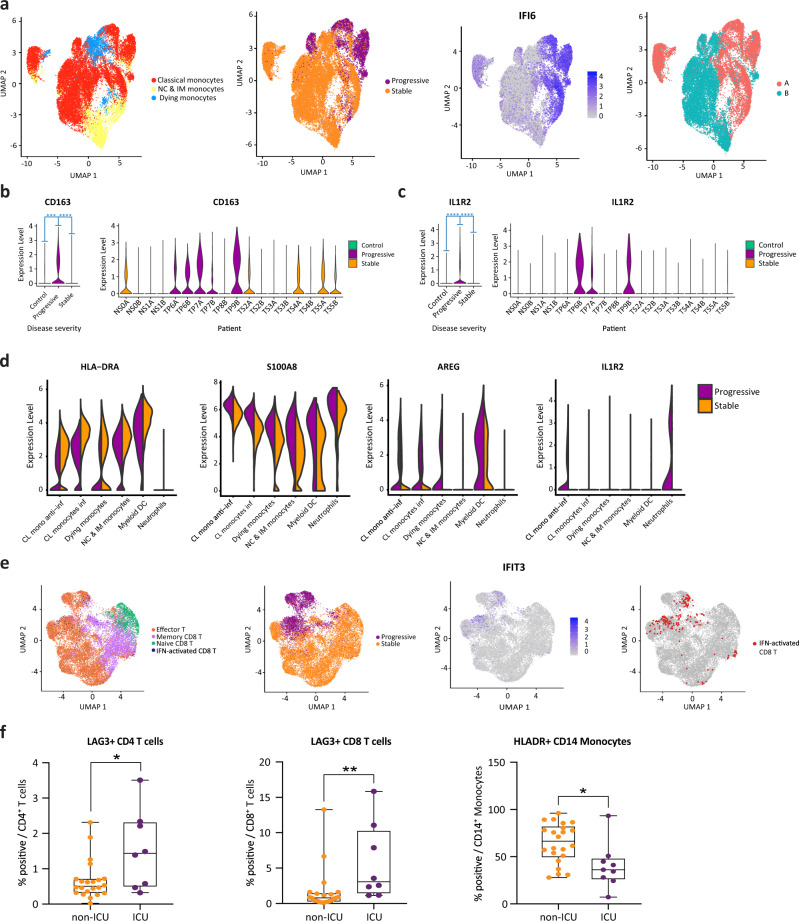
Progressive COVID-19 is associated with an immune dyssynchrony in monocytes and T-cells. **a** Four congruent UMAPs showing sub-clustering results of all monocytes. The left panel shows the original cell annotation. Cells from progressive patients concentrate in the high ISG pole (IFI6 is given as a representative example of ISGs). The right panel highlights a clear separation between cells from the earlier blood draw (time point A) and those from the later one (time point B) which follows the ISG expression pattern. **b**, **c** *CD163* and *IL1R2* are increased in progressive COVID-19. ***p-value < 1E−200; ****p-value < 1E−300 calculated by Wilcoxon rank-sum test with Bonferroni correction for multiple comparisons. **d** Violin plots showing the expression of *HLA-DRA, S100A8, AREG,* and *IL1R2* in myeloid cell clusters, comparing stable to progressive patients. **e** Four congruent UMAPs depicting sub-clustering results of CD8^+^ T cells. The left panel shows the original cell annotation. Cells from progressive patients show a clear separation from those of stable ones, and seem to concentrate at the high ISG pole (*IFIT3* is given as a representative example for ISGs). Most of the cells belonging to the IFN-activated CD8^+^ T cluster are located in the high IFN/progressive pole (right panel). **f** LAG-3 and HLA-DR levels were measured in the indicated cell types by flow cytometry, in an independent cohort of patients. Shown are % positive for these markers out of total cells of the same cell type, comparing patients that were admitted to the intensive care unit (ICU, comparable to progressive patients, *n* = 8 for LAG-3 analysis and *n* = 9 for HLA-DR analysis) and those that were not (non-ICU, comparable to stable patients, *n* = 22). The results are depicted in boxplots, in which the value for each patient is represented by a dot, the upper and lower bounds represent the 75% and 25% percentiles, respectively, the center bars indicate the medians, and the whiskers denote values up to 1.5 interquartile ranges above the 75% or below the 25% percentiles. *p-value < 0.05; **p-value < 0.01, as assessed by two-tailed Mann–Whitney test. Source data are provided as a Source Data file.

In order to better understand the transcriptional differences between stable and progressive COVID-19 monocytes, we sub-clustered them after excluding cells from control subjects. This yielded 23,701 monocytes in seven clusters (Fig. 4a and Supplementary Fig. 10). We identified a clear separation between cells of stable and progressive patients, which is driven in part by increased expression of ISGs in progressive patients (Figs. 3a, 4a and Supplementary Data 1). Regulatory and tissue repair-associated genes are increased in progressive vs stable monocytes, including *CD163* (Fig. 4b)*, IL1R2* (Fig. 4c)*, AREG* (Fig. 4d), the co-inhibitory receptor *HAVCR2* (encoding TIM-3), and its ligand *LGALS9* (encoding Galectin-9; Fig. 3b), and *IL10* (Supplementary Fig. 8). The expression of *RNASE2*, encoding a protein with antiviral activity (mainly against single-stranded RNA viruses)^36^, is also increased in progressive patients (Fig. 3a). Of note, *LGALS9* expression was increased not only in myeloid cells but also in B and CD4 T cells in progressive patients (Fig. 3b and Supplementary Fig. 9). This indicates a potential role for the TIM-3/Gal-9 pathway in myeloid/T cells interaction that enhances the regulatory phenotype of myeloid cells in progressive patients, as observed in cancer patients^37,38^.

MHC-II molecules were decreased in progressive monocytes as detailed above (Figs. 3a, c, e and 4d). The alarmins S100A8/S100A9 are ranked among the top DEGs increased in progressive vs stable monocytes (Figs. 3a and 4d), as also shown by recent scRNA-seq COVID-19 studies^39,40^, and as observed in SARS-CoV infection^41^. Of note, S100A8/9 expression is also influenced by tocilizumab treatment as discussed in the tocilizumab effects section below. Given that S100A9 is a marker of myeloid-derived suppressive cells (MDSCs)^42^ and can promote IL-10 production and suppressive capacity of MDSCs^43,44^, the signature of monocytes in progressive patients somewhat resembles that of MDSCs^45^. Indeed, one of the two classical monocyte clusters (cluster #1, Fig. 1f—dashed box) is enriched with MDSCs associated genes (*S100A8, S100A9, IL1R2, IL10*) with low expression of MHC-II. Furthermore, this monocyte cluster exhibits highly overlapped transcriptional features with a recently identified monocyte population in severe sepsis^46^. Unexpectedly, pro-inflammatory monocyte markers such as *IL1B* and *TNF* are downregulated in COVID-19 monocytes relative to controls (Supplementary Fig. 11), both in progressive and in stable patients, although *IL1B* was slightly less downregulated in stable patients. This observation is consistent with recent reports highlighting an immunosuppressive phenotype in severe respiratory failure in COVID-19 patients^47^.

Taken together, these findings revealed a skewed regulatory signature of monocytes in progressive patients, which resembles immunoparalysis^48^. Given that many of these genes associated with an immunosuppressive phenotype are regulated downstream of IFN-I signaling (*AREG, IL1R2, S100A8, S100A9, IL10*), this shift of classical monocytes toward MDSC-like suppressive cells might stem from the strong IFN-I response. In addition, our connectome analysis highlights the enhanced TIM-3/Gal-9 circuit, which may contribute to the aberrant regulatory myeloid signature. This potentially premature shift to a resolution phase might interrupt appropriate antiviral immune responses, contributing to the delayed virus clearance and deleterious clinical manifestations observed in severe COVID-19^4^.

### CD8^+^ T cells exhibit an enhanced effector signature in progressive patients

We next attempted to examine the gene expression differences in CD8^+^ T-cell subpopulation between the disease conditions. A detailed analysis of sub-clustered 19,458 CD8 T cells (Fig. 4e and Supplementary Fig. 12a) showed a clear separation between stable and progressive patients, driven mainly by a higher expression of ISGs in the progressive patients (Figs. 3a and 4e), but also by higher expression of effector cytokines such as *GZMB* (Fig. 3a). Most of the cells from the IFN-activated CD8^+^ T cell cluster are located in the progressive pole and overlap with the effector T cell cluster (Fig. 4e). There are clear shifts in the gene expression profile from the early time-point A to the late time-point B (Supplementary Fig. 12b–d) that are mainly driven by the decrease in ISG signature (Fig. 2a). The differential connectivity map analysis demonstrates an increased expression of the co-inhibitory receptor *LAG3* in T lymphocytes of progressive patients, while its ligands, which are MHC-II molecules, are decreased in antigen-presenting cells (Fig. 3b, c). This mismatch, which was validated by flow cytometry (Fig. 4f), is part of the immune system dyssynchrony we observed in progressive patients that required ICU admission.

### Tocilizumab effects differ across cell types and associate with levels of *IL6R* and *IL6ST*

**Fig. 5:**
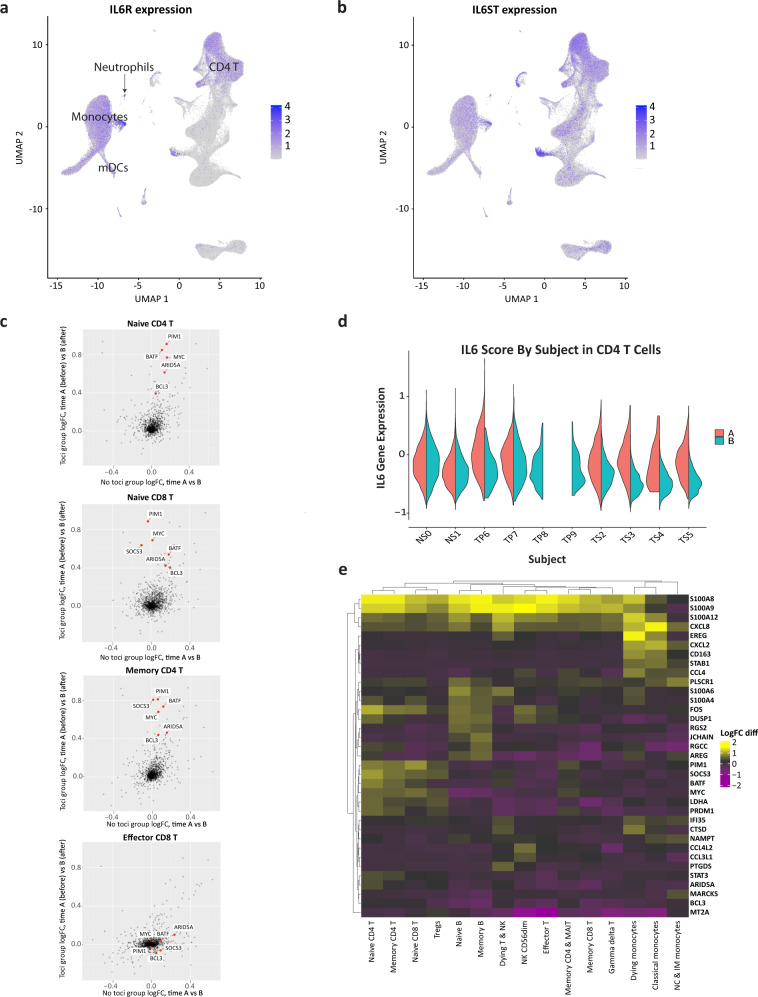
Tocilizumab exerts differential gene expression effects in different immune cells. **a**, **b** UMAP representations of *IL6R* (**a**) and *IL6ST* (**b**) expression in PBMCs. Note that *IL6R* is highest for monocytes, dendritic cells, CD4^+^ T cells (including Tregs), and naive CD8^+^ T cells, while *IL6ST* expression is similar in the majority of cell types. **c** Scatter plots of the logFC from time-point A to B in patients treated (*Y* axes) compared to those not treated with tocilizumab (*X* axes) for several T cell subtypes. This comparative model demonstrates a marked effect of tocilizumab on IL-6 pathway genes (shown in red) in CD4^+^ and naive CD8^+^ T cells, but not in effector CD8^+^ T cells, in which *IL6R* expression is low (see Supplementary Fig. 13 for the full panel with all cell types). **d** IL-6 score in CD4^+^ T cells is decreased at time-point B in all the patients that were treated with tocilizumab, but not in the non-treated patients (NS0, NS1). **e** A heatmap showing the expression of genes that were significantly differentially expressed (LogFC > 0.4, adjusted *p*-value < 0.05 calculated by Wilcoxon rank-sum test with Bonferroni correction for multiple comparisons) between time point A and B in tocilizumab-treated patients, but not in patients that were not treated by tocilizumab, across PBMC subtypes. All the entries in the heatmap matrix are the differences of logFC in tocilizumab and in non-tocilizumab groups. Also shown is a hierarchical clustering according to cell types (horizontal) and individual genes (vertical).

Eight of ten COVID-19 patients in our study were treated with tocilizumab, an anti-IL-6 receptor (IL-6R) antibody. We further examined the differential gene expression pattern that is associated with tocilizumab treatment. *IL6R* is highly expressed in monocytes, dendritic cells, neutrophils, CD4^+^ T cells (including FoxP3 regulatory T cells (Tregs)) and naive CD8^+^ T cells (Fig. 5a). On the other hand, *IL6R* expression is low in the other types of lymphocytes including memory CD8^+^, effector CD4^+^ & CD8^+^ T cells, gamma-delta T cells, B cells and NK cells. *IL6ST* (encoding gp130), responsible for signal transduction of IL-6 following binding to IL-6R, is expressed in all types of PBMCs (Fig. 5b). To identify the transcriptional effects of tocilizumab treatment in COVID-19 patients, we compared gene expression changes from time point A to B for patients in the tocilizumab treatment group versus those not treated with tocilizumab (Fig. 5c and Supplementary Fig. 13). We highlight six tocilizumab responsive genes (*ARID5A, BCL3, PIM1, SOCS3, BATF, MYC*) that are associated with IL-6 pathway and known to be perturbed by tocilizumab treatment in rheumatoid arthritis patients^49^. Of note, those transcriptional changes by tocilizumab are observed mainly in the cell types that highly express *both IL6R* and *IL6ST*, such as naive CD4^+^ T cells, memory CD4^+^ T cells, naive CD8^+^ T cells, and Tregs. To quantify this effect, we generated an IL-6 score (a composite score of the aforementioned six tocilizumab responsive genes). We demonstrated a significant decrease of IL-6 score in CD4^+^ T cells in all patients who received tocilizumab, but not in ones who did not (Fig. 5d and Supplementary Data 10).

Next, we sought to identify the other genes that are perturbed by tocilizumab in COVID-19 patients. To minimize the confounding effects of disease-related gene expression changes over time, we focused on genes that are not decreased over time in the non-tocilizumab group but significantly decreased following tocilizumab treatment (log-fold-change [logFC] > 0.4, Fig. 5e). We demonstrate that *S100A8* and *S100A9* expression are highly downregulated by tocilizumab treatment across the majority of the cell types, but not changed or even slightly increased in non-tocilizumab group, leading to a large logFC difference (Fig. 5e). Given that a positive feedforward loop between S100A8/9 and IL-6 can drive pro-inflammatory circuit^50–52^ and that elevated serum S100A8/9 is one of the hallmarks of severe COVID-19 patients^53,54^, it is possible that tocilizumab can exert its effect partly through the inhibition of S100A8/9 expression in COVID-19. Of interest, the expression of *IL6R* is higher than that of *IL6ST* in myeloid cells, while it is lower in all other cell types, leading to a difference in *IL6R/IL6ST* ratio. According to a recent study^55^, this ratio determines the type of response to IL-6 signaling: anti-inflammatory classical signaling in cells with high *IL6R/IL6ST* (as observed in our myeloid cells) or pro-inflammatory trans-signaling in cells with low *IL6R/IL6ST* (non-myeloid cells), possibly explaining the observed difference between cell types in response to tocilizumab (Fig. 5e). While we detected a response to tocilizumab at a cellular level, our study was neither designed nor powered to detect any clinical effect of the therapy. Nonetheless, these gene expression patterns may suggest a link between tocilizumab effects, *IL6R, IL6ST,* and S100A8/9 in COVID-19 patients.

### Surface protein-based immune-phenotyping of peripheral blood cells in COVID-19

We next constructed an independent immunophenotypic map of PBMCs using CITE-seq^18^. To better identify cellular multiplets and enable us to super-load the cells onto the 10× platform, we used Cell Hashing technique and multiplexed 5-6 samples in each 10× reaction. We adopted 189 oligonucleotide-labeled antibodies (Total seq C antibody panel from BioLegend) (Supplementary Data 5). Multiplets and cells with unidentifiable sample origin were removed from the downstream analysis and 83.2% of the cells were analyzed (*n* = 43,349; Supplementary Data 6). Following unsupervised clustering, annotation for CITE-seq cells was performed with both gene expression and antibody-derived counts (ADT) by using a manually curated marker gene list (Supplementary Data 7). We observed a cluster of undefined cells (*n* = 8032) that are positive for multiple linage markers of ADT signals and/or display elevated signals unlikely to be explained by immunological evidence. Those cells were removed from the analysis, and all other cells were plotted on UMAP space (Supplementary Fig. 14a). In order to investigate the coherence of both annotations, we calculated the percentage of shared cells between RNA and ADT-annotated cell types (Supplementary Fig. 14b).

### HLA-DR^+^CD38^+^ T cells express higher co-inhibitory receptors in progressive patients

**Fig. 6:**
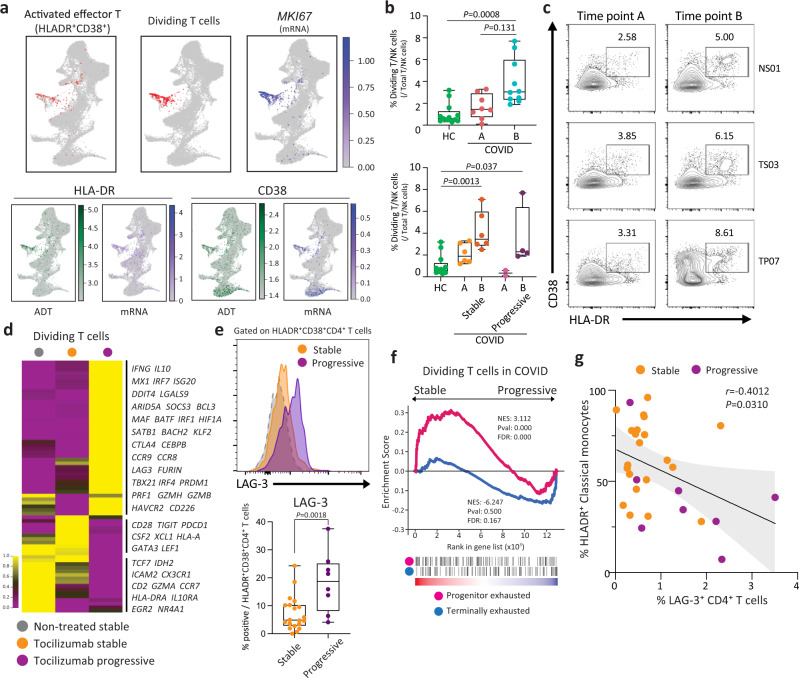
Multi-omics immune profiling identifies HLA-DR^+^CD38^+^ T cells in progressive COVID-19 patients. **a** Visualization of ADT cluster #15 (HLA-DR^+^CD38^+^ activated effector T cells) and TCR^+^ cells in GEX dividing T cells cluster on the GEX UMAP (red highlighted). ADT and RNA expression of *MKI67* (only RNA), HLA-DR/*HLADRA*, and CD38/*CD38* projected on GEX UMAP. **b** Boxplots show frequency of dividing T cells within the total T cell population at early and late time-points (top; *n* = 13 for HC, 8 for time A, and 10 for time B), and in stable and progressive patients at early and late time-points (bottom; *n* = 13 for HC, 6 for Stable-A, 6 for Stable-B, 2 for Progressive-A, and 4 for Progressive-B). One-way ANOVA and Sidak’s multiple comparisons test. **c** Representative contour plot of flow cytometry analysis of PBMCs from COVID-19 patients (left). The frequency of HLA-DR^+^CD38^+^ cells assessed by ADT (top right) and flow cytometry (bottom right) in samples from stable and progressive COVID-19 patients is shown. **d** Heatmap representation of DEGs in dividing T cells across three patient groups. **e** Representative histogram of LAG-3 expression on HLA-DR^+^CD38^+^ CD4^+^ T cells from stable and progressive patients assessed by flow cytometry. Gray shaded plot represents a fluorescence minus one staining for LAG-3 (top). Quantification of LAG-3 positive cells within HLA-DR^+^CD38^+^ CD4^+^ T cells (bottom). Mann–Whitney *U* test (two-tailed). **f** GSEA of a signature of progenitor exhausted versus terminally exhausted CD8^+^ T cells in chronic infection (GSE84105). Progenitor exhausted signature (red), terminally exhaustion signature (blue), in the ranked list of genes differentially expressed by dividing T cells from stable versus progressive patients. **g** Correlation between frequency of LAG-3 positive CD4^+^ T cells and HLA-DR^+^ classical monocytes in COVID-19 patients. Pearson’s *r*, exact two-sided *p-*value, and the 95% confidence interval are shown. The results in **b** and **e** are depicted in boxplots, in which the value for each patient is represented by a dot, the upper and lower bounds represent the 75% and 25% percentiles, respectively. The center bars indicate the medians, and the whiskers denote values up to 1.5 interquartile ranges above the 75% or below the 25% percentiles. Source data are provided as a Source Data file.

Among the overlapping cell types, we found that 49% of ADT-annotated activated effector T cells cluster (HLA-DR^+^CD38^+^, ADT cluster #15) are overlapped with GEX dividing T/NK cluster, indicating expression of both *HLADRA*/*CD38* and *MKI67* marks a unique T cell subset in COVID-19 (Fig. 6a and Supplementary Fig. 14b). Dual expression of HLA-DR and CD38 or a higher expression of Ki67 are known to mark a highly activated T cell population in acute viral infection^56–59^. We observed that *MKI67*-expressing TCR^+^ T cells within the GEX dividing T/NK cluster were increased in COVID-19 patients, which was further validated by using flow cytometry with the same samples (Fig. 6b, c) and a different cohort, specifically in CD4^+^ T cells from progressive patients (Supplementary Fig. 14c, d). This observation is supported by a recent study using flow cytometry with a larger number of COVID-19 patients^60^.

CITE-seq technology further allowed us to elucidate the transcriptional signature of this activated T cell population. Compared to the other T cell clusters, the HLA-DR^+^CD38^+^ T cell cluster #15 exhibits enriched expression of co-inhibitory receptors (*LAG3, CTLA4, PDCD1, ENTPD1, HAVCR2*)^61,62^ and lower expression of naive/stemness markers (*TCF7, LEF1*)^63–65^ and cytotoxic T cell markers (*NKG7, KLRG1, PRF1, GZMH*) (Supplementary Fig. 14e). Transcription factors (TFs) promoting T cell exhaustion (*PRDM1, MAF*)^66^ are also enriched in this cluster. These data suggest that T cells in this cluster display skewed transcriptional signature toward terminal differentiation.

We next determined whether there are transcriptional differences in this activated T cell cluster between stable and progressive COVID-19 patients. Progressive patients exhibited higher expression of IFN-I response genes (*MX1, IRF7, ISG20*) and cytotoxic/pro-inflammatory cytokines (*PRF1, GZMH, IFNG*), and lower expression of stemness/progenitor markers (*TCF7, LEF1*). Interestingly, while most of the co-inhibitory receptors were enriched in progressive patients (*LAG3, CTLA4, HAVCR2*), some were enriched in stable patients (*PDCD1, TIGIT*). Exhaustion/effector driving TFs (*PRDM1, MAF*) and the immunoregulatory cytokine *IL10*, which is also co-expressed in exhausted T cells, were upregulated in progressive patients (Fig. 6d). We found that LAG-3 was the most upregulated co-inhibitory receptor in T cells specifically in activated T cells from progressive COVID-19 patients, which is validated by flow cytometry with a different cohort of COVID-19 patients^4^ (Figs. 4f and  6e). Given that the higher expression of co-inhibitory receptors mark exhausted T cells and recent studies demonstrated exhaustion-like gene expression patterns observed in T cells in COVID-19^23,67^, we sought to determine the gene expression signature of these dividing T cells in progressive patients by using gene set enrichment analysis (GSEA). Dividing T cells in progressive patients exhibited more terminally exhausted T cell signature and IFN-I response signature than those in stable patients (Fig. 6f, Supplementary Fig. 14f, and Supplementary Data 8). Of note, this transcriptional signature in progressive COVID-19 patients overlapped with that of HIV-specific T cells from HIV progressors compared to HIV controllers (Supplementary Fig. 14f). Although it is too early to observe T cell exhaustion at this acute phase of viral infection, given that the IFN-I pathway is implicated to facilitate the T cells exhaustion in both tumor infiltrated T cells^63^ and chronic viral infections^68,69^, our data suggest that the stronger or prolonged IFN-I response in progressive COVID-19 patients may promote T cell differentiation prematurely.

In light of these observations, we further sought to understand the alteration of immune cell interaction between stable and progressive patients. Among the ligands of LAG-3, we observed significant decreases of MHC-II molecules on myeloid cells and B cells in progressive COVID-19 patients, which is also highlighted in our differential connectome analysis in progressive versus stable COVID-19 patients (Fig. 3c). Flow cytometry analysis demonstrated the negative correlation between LAG-3 on CD4^+^ T cells and HLA-DR on CD14^+^ classical monocytes (Fig. 6g), suggesting that altered LAG-3/MHC-II interaction might play a role in disease progression.

Taken together, our scRNA-seq analysis and flow cytometry-based validation revealed the increase of an activated T cell population marked by higher expression of LAG-3 in COVID-19 patients. Furthermore, transcriptional analysis of this population demonstrated a terminally differentiated T cell-like signature in progressive COVID-19 patients with higher co-inhibitory receptor expressions. Unbalanced LAG-3/MHC-II interactions between T cells and antigen-presenting cells may reflect the failure of appropriate innate-adaptive cells interaction, resulting in aberrant expression of cytotoxic cytokines that may, in turn, contribute to immunopathology^4^.

### Skewed T cell receptor repertoire in CD8^+^ T cells of progressive patient

**Fig. 7:**
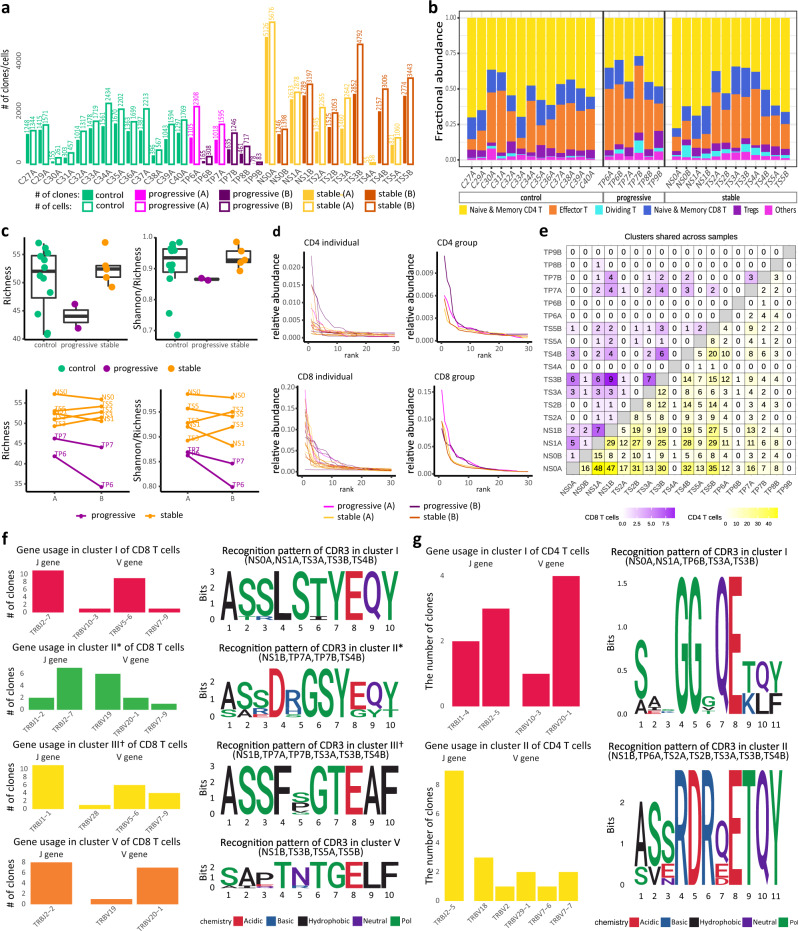
TCR data analysis of COVID-19 patients and controls. **a** The number of cells and clones across all samples. Cells with TCR data of low quality are excluded. **b** Fractional abundance of cells with high-quality TCR data among different cell types. **c** Rarefied diversity indices (richness and evenness) of the naive and memory CD8^+^ T cells at time point A are significantly different between stable (*n* = 5) and progressive (*n* = 2) patients presented in boxplots (top panels). *p*-value = 0.045 for richness and 0.005 for evenness by one-sided Student’s *t*-test. The results are depicted in boxplots, in which the value for each patient is represented by a dot, the upper and lower bounds represent the 75% and 25% percentiles, respectively. The center bars indicate the medians, and the whiskers denote values up to 1.5 interquartile ranges above the 75% or below the 25% percentiles. Changes in these diversity indices after treatment are shown between stable (*n* = 5) and progressive (*n* = 2) patients (bottom panels). *p*-value = 0.283 for richness and 0.240 for evenness by Student’s *t*-test. **d** Rarefied relative abundance of the top 30 clones from CD4^+^ T cells (top panels) and CD8^+^ T cells (bottom panels) between stable and progressive patients. **e** The number of clone clusters identified by GLIPH2 in CD4^+^ T cells (lower triangle) and CD8^+^ T cells (upper triangle) that have clones from every pair of samples based on the top 24 CD8^+^ and 172 CD4^+^ T cell SARS-CoV-2-specific clone clusters. **f** Clone clusters’ TRBV and TRBJ gene usage distribution in CD8^+^ T cells based on the 4 chosen SARS-CoV-2-specific expanded clone clusters. The CDR3β motif found in each cluster with global similarity is shown as well as the samples that contribute clones to the cluster. * Clusters with dividing CD8^+^ T cells. ^†^ Clusters with IFN-activated CD8^+^ T cells. **g** Clone clusters’ TRBV and TRBJ gene usage distribution in CD4^+^ T cells based on the 2 chosen SARS-CoV-2-specific expanded clone clusters. The CDR3β motif found in each cluster is shown as well as samples that contribute clones to them. Source data are provided as a Source Data file.

In order to characterize the T cell receptor (TCR) repertoire relevant for immunity to SARS-CoV-2, we conducted a single-cell V(D)J analysis of COVID-19 and control samples. TCR data was captured for 67,393 cells in total with a median of 1954 cells per sample. Quality assessment and control of the data filtered out 8303 cells, leaving a median of 1778 cells per sample. Based on these high-quality data, cells with the same V(D)J sequences of beta and alpha chains from the same subject were grouped into clones. In total, 41,742 unique clones were identified with a median of 1297 clones per sample (Fig. 7a). The cell-type composition of these cells for each sample is shown in Fig. 7b.

Alpha diversity with rarefaction was calculated using the Alakazam R package^70^ for both memory and naive CD4^+^ T cells and memory and naive CD8^+^ T cells. The diversity of memory and naive CD8^+^ T cells at both time points showed lower richness (Student’s *t*-test *p*-value = 0.045) and evenness (i.e., Shannon index/richness, Student’s *t*-test *p*-value = 0.005) in progressive patients than stable patients (Fig. 7c), which is consistent with the higher expansion of CD8^+^ T cell clones in progressive patients (Fig. 7d)^16,40,71^. This difference in alpha diversity was not observed in memory and naive CD4 T cells. The change in CD4^+^ and CD8^+^ T cell clonal richness and evenness between time-point A and B were not significantly different between progressive and stable patients, possibly due to the small number of progressive patients with data at both time points. (Fig. 7c, bottom panel).

### Identification of COVID-19-specific CDR3 regions

In order to identify characteristics of the TCR regions that may confer specificity to SARS-CoV-2, we used GLIPH2^72^ to assess the similarity of complementarity-determining region 3 (CDR3) sequences among COVID-19 patients. We specifically looked for CDR3 motifs in β chains that were shared across several COVID-19 patients but in none of the 13 control subjects. Stringent filters were applied to the GLIPH2 CDR3 specificity groups (or clusters) to improve accuracy, including requiring Fisher’s score < 0.0001, ≥3 unique TCRs in the specificity group, and significant V-gene bias (*p* < 0.05). The filtered specificity groups with any clone from control samples were filtered out to enhance the likelihood of specificity to SARS-CoV-2 instead of to other common viruses such as cytomegalovirus. After heavy filtering, 24 and 172 groups remained for CD8^+^ and CD4^+^ T cells, respectively. Most of the identified specificity groups included clones from different samples, suggesting a large similarity in the CDR3 sequence in the potential SARS-CoV-2-specific clones (Fig. 7e and Supplementary Fig. 15). To further enhance the specificity to clonally-expanded SARS-CoV-2 responsive T cells, we focused on 10 CD8^+^ and 12 CD4^+^ T cell groups that have clones from ≥3 subjects with at least one of these clones with ≥2 cells. The V and J gene usage analysis showed a strong usage bias for J gene in 3 CD8^+^ groups and 1 CD4^+^ group (Fig. 7f, g). Some VJ combinations showed a dominant usage such as TRAV5/TRAJ12/TRBJ2-7/TRBV5-6 for cluster 1 in CD8^+^ T cells (Fig. 7f and Supplementary Figs. 16, 17). For the CD4^+^ T groups, there is no obvious V gene usage bias and the J gene usage is dominated by TRBJ2-5 (Fig. 7f, g).

Among the 10 and 12 putative SARS-CoV-2-specific and expanded groups, we further chose those that include clones from ≥3 different COVID-19 patients with ≥55% clones having more than one cell, resulting in five and two groups for CD8^+^ and CD4^+^ T cells, respectively. The chosen clusters were also the top five and two clone clusters with the best composition score by GLIPH2, which measures the strength of a specificity group based on global/local similarities, enrichment of common V-genes, a limited CDR3 length distribution, expanded clones (ECs), and cluster size. This suggests that the chosen specificity groups are likely from SARS-CoV-2-specific ECs and shared across COVID-19 patients with a highly conserved CDR3 amino acid (AA) sequence. All specificity groups are identified based on global similarities in the CDR3 region, except for cluster IV in CD8^+^ T cells, whose member clones have different CDR3 lengths but share the motif “QDIG”. The CDR3 sequence motifs of the specificity groups with global similarity are shown in Fig. 7f, g. We confirmed that our samples were not biased by HLA genotype (Supplementary Figs. 18 and 19). The CDR3 motifs in Fig. 7f, g were compared to those found in two recent SARS-CoV-2 studies with TCR repertoire data^73,74^, which collected samples mainly from recovered and convalescent SARS-CoV-2 patients. The comparison showed that our CD8^+^ specificity group V motif (TNTGE) had a similar pattern to a motif (TGTGE) found in Schultheiß et al. ^74^. The study did not find this motif among the top 31 motifs shared among recovered SARS-CoV-2 patients but found it shared between longitudinal samples during active disease and at recovery from one patient with mild disease and recovered patients, suggesting the specificity of this motif to SARS-CoV-2. This overlap validates the specificity of our CD8 group V motif to SARS-CoV-2 infection. It also demonstrates the power and importance of our TCR analysis due to sample collection during active disease and GLIPH2 analysis with the exclusion of specificity groups present in control samples.

### Single-cell V(D)J B cell receptor repertoire analysis

**Fig. 8:**
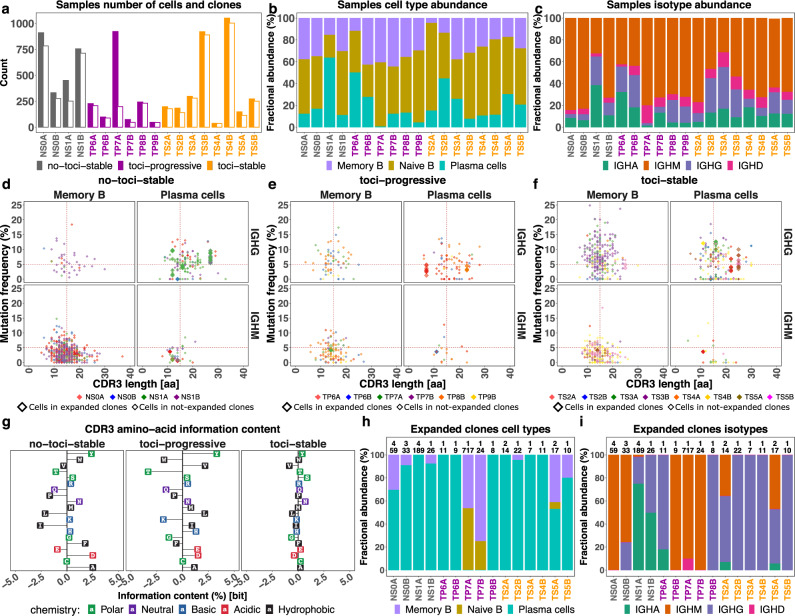
BCR data analysis, part 1. **a** Number of cells (closed-bars) and number of clones (open-bars) in patients colored based on the treatment and status of the disease. **b** Fractional abundance of memory B cells, naive B cells, and plasma cells in each sample. **c** Fractional abundance of isotypes (IGHM/D/G/A) in each sample. **d**–**f** CDR3 amino acid length (*x* axis) and mutation frequency (*y* axis) for each cell type (memory B cell and plasma cell columns) and isotype (IGHG and IGHM rows) of stable patients with no treatment, progressive patients under treatment, and stable patients under treatment. Colors indicate different samples. Vertical dashed line represents 15 amino acid CDR3 length reference point and horizontal line represents 5% mutation frequency reference point. Points with larger size belong to the expanded clones. **g** CDR3 amino acid usage at time-point B relative to A for patients grouped based on the treatment and status of the disease. **h** Cell type fractional abundance of expanded clones in each sample. Labels in each bar represent the number of expanded clones (top) and number of cells (bottom). **i** Isotype fractional abundance of expanded clones in each sample. Labels as described in **h**. Source data are provided as a Source Data file.

For each sample, a summary of the number of cells, frequency of each B cell type (naive B, memory B, and plasma cells), and frequency of each isotype (IGHM/D/G/A) is provided in Fig. 8a–c, respectively. Overall, the single-cell V(D)J library contains 7177 cells distributed across 18 samples. Gene usage and mutation frequency dynamics across three cell types, per each patient and time point, are shown in Supplementary Figs. 20 and 21, respectively.

### COVID-19 patients with stable status show higher mutation frequency and longer CDR-H3 length

IGHV/IGHJ mutation frequency and CDR-H3 length varied by antibody isotype and cell type within COVID-19 patients (Fig. 8d–f). IGHM memory and plasma B cells had mutation frequencies significantly lower than 5% (mutations/nucleotide, *p*-value < 0.01), regardless of treatment or disease progression group. As expected, IGHG memory B cells and plasma cells had mutation frequencies higher than IGHM cells. In particular, plasma cells in stable COVID-19 patients without treatment had mutation frequencies significantly higher than 5% (mean = 5.6 ± 3%; *p*-value < 0.01). Memory cells in stable patients under tocilizumab treatment had even higher mutation frequencies (mean = 7.5 ± 4.6%).

The CDR-H3 length of IGHG and IGHM B cells generally varied between 10 and 20 AAs (we used 15 AAs as a reference point for downstream comparisons) across all cell types (Fig. 8d–f). However, the CDR-H3 length of IGHG plasma cells in stable COVID-19 patients was significantly larger than 15 AAs (*p*-value < 0.01), while the CDR-H3 length of IGHG memory cells do not show significant differences from 15 AAs (mean = 15.2 ± 3.9 AAs across all samples). On average, CDR-H3 lengths of IGHG plasma cells were larger in stable no-tocilizumab patients (mean = 18.4 ± 5.4 AAs) than in stable tocilizumab-treated patients (mean = 17.2 ± 5 AAs).

### Stable patients under treatment do not show change in CDR-H3 amino acid usage

We sought to investigate the differences in CDR-H3 AA usage between the two blood draw time points (A and B) (Fig. 8g). We tackled this query by calculating the conditional information content (IC) of each AA in the CDR-H3 segment at time point B with respect to time point A (see “Methods” section). We averaged the conditional ICs for patients belonging to three different groups: (1) stable patients under no treatment (no-tocilizumab-stable); (2) progressive patients under tocilizumab treatment (tocilizumab-progressive); and (3) stable patients under tocilizumab treatment (tocilizumab-stable). Our results indicate that the profile of AA usage for tocilizumab-stable patients is quite different from the other groups. In fact, the IGH repertoires of tocilizumab-stable patients do not show any change in preferences toward the usage of specific AAs in their CDR-H3 segment between the time points. In contrast, the IC profiles of tocilizumab-progressive and no-tocilizumab-stable patients vary across AAs. In particular, tocilizumab-progressive and no-tocilizumab-stable patients show evidence of increased usage of alanine (A), aspartic acid (D), and tyrosine (Y), and decreased usage of proline (P), glutamine (Q), and threonine (T) at time-point B relative to A.

### High frequency of plasma cells with expanded clonal lineages in COVID-19

To explore antigen-driven B cell responses in the COVID-19 patients, we investigate expanded clonal lineages (Fig. 8h, i). We identified 20 expanded clones (ECs, as defined in Methods section) with members found in 15/18 samples containing 1157/7177 cells (16% of all B cells). Plasma cells were significantly enriched in the ECs (mean = 78% across all samples; mean odds ratio = 5.7; *p*-value < 0.01) (Fig. 8h). ECs in samples TP7A and TP7B did not contain any plasma cells. IGHG cells were significantly enriched in ECs from tocilizumab-stable patients (mean = 84% across 6 samples, mean odds ratio = 8.2; *p*-value < 0.01) IGHM cells were enriched in ECs from tocilizumab-progressive patients (mean = 58% across 5 samples, mean odds ratio = 1.2) (Fig. 8i).

We further investigated the mutation frequency and CDR-H3 length of cells within ECs (Fig. 8d–f). We observed that the mutation frequency of IGHG plasma cells from stable patients was higher on average (mean = 5.5 ± 4%) than that in progressive patients (mean = 3.2 ± 0%), regardless of treatment status. The CDR-H3 length of IGHG plasma cells from stable patients was significantly larger than 15 AAs (*p*-value < 0.01). In particular, patients without treatment had a larger mean CDR-H3 length (mean = 20.2 ± 6.6 AAs) than those under treatment (mean = 17.5 ± 5.4 AAs). IGHG plasma cells from progressive patients had CDR-H3 lengths significantly shorter than 15 AAs (mean = 14.5 ± 8.2 acid AAs; *p*-value < 0.01).

**Fig. 9:**
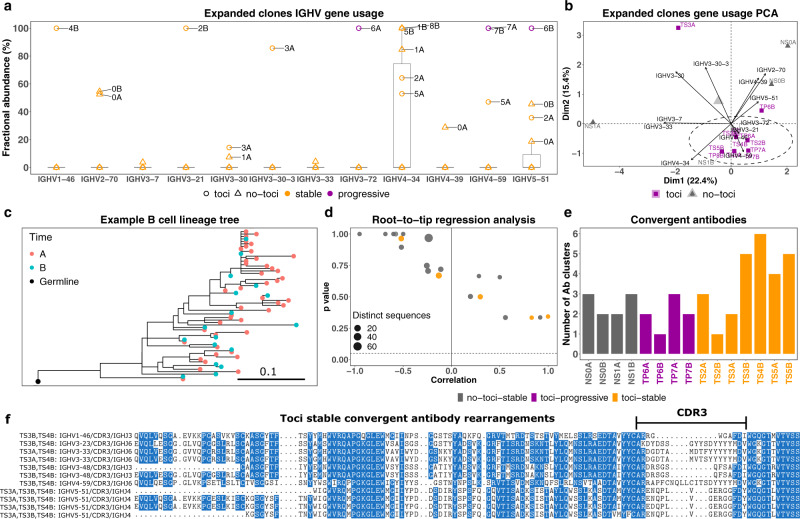
BCR data analysis, part 2. **a** IGHV gene (*x* axis) fractional abundance (*y* axis) of expanded clones in each sample (label). Colors and shapes represent patients grouped based on the status of the disease and treatment. The results are depicted in boxplots, in which the value for each patient is represented by a dot, the upper and lower bounds represent the 75% and 25% percentiles, respectively. The center bars indicate the medians, and the whiskers denote values up to 1.5 interquartile ranges above the 75% or below the 25% percentiles. Data beyond the end of the whiskers are outliers. *N* = 10 for Stable and 5 for Progressive. **b** PCA of IGHV genes (arrows label) based on the fractional abundance from expanded clones of each sample (points label). Colors and shapes represent patients grouped based on treatment. **c** An example of a B cell clonal lineage tree. Branch lengths represent the expected number of substitutions per codon (see scale bar). **d** A root-to-tip correlation analysis. Pearson correlation coefficient between divergence and time within each B cell lineage tree (*x* axis), with corresponding *p*-values calculated using a permutation test (*y* axis). The size of each point corresponds to the number of distinct sequence/time point combinations within each clone. Dashed line shows *p*-value = 0.05. **e** Number of convergent antibody clusters within each sample. **f** Convergent antibodies (VDJ) that are specific to patients with stable status and under treatment. Source data are provided as a Source Data file.

Selection of particular IGHV genes in response to a particular antigen has been observed in other antiviral responses, such as the preference for IGHV1-69 in response to some influenza virus antigens^75,76^. Therefore, we sought to identify IGHV genes under selection in COVID-19 patients (Fig. 9a). IGHV4-34 gene was highly used in ECs of stable patients with odds ratio of ~10 among patients under treatment and ~9.5 among patients without treatment. Progressive patients showed a lower usage of IGHV4-34 with an odds ratio of ~5.9. We further performed principal component analysis (PCA) of IGHV gene usages in expanded B cell clones (Fig. 9b). We have identified a cluster of patients under treatment, including both stable and progressive, whose corresponding ECs only contain IGHV1-46 (100% in TS4B), IGHV3-21 (100% in TS2B), IGHV3-30-3 (85% in TS3A), and IGHV3-72 (100% in TP6A).

### Unmutated IGHG clones and large clones with stable SHM frequency characterize severe COVID-19

BCR sequence analysis can provide important information about the dynamics of the B cell response within COVID-19. Prior work^77^ has demonstrated an elevated proportion of unmutated (median SHM < 1%) IGHG B cell clones in COVID-19 patients compared to healthy controls. This could be an indication of early class switching before GC entry in a primary immune response. We similarly observed that between 0% and 45.7% of IGHG clones within each patient were unmutated (mean = 19.8%; Fig. 8d and Supplementary Fig. 22), considering both expanded and non-expanded clones. Further, tocilizumab-stable patients had a higher fraction of unmutated IGHG clones compared to tocilizumab-progressive patients (mean = 30.8% vs 11.3%, respectively), though this difference was not significant (Supplementary Fig. 22, *p*-value = 0.057, Wilcoxon test). A recent analysis has shown that this expansion of unmutated plasmablasts is characteristic of hospitalized, rather than mildly symptomatic, COVID-19 patients^78^. These clones were primarily composed of memory and plasma cells, in contrast to IGHM clones, which were primarily composed of IGHM cells (Supplementary Fig. 22). We also observed multiple diverse B cell clones, both expanded and non-expanded, spanning both time points. To characterize potential affinity maturation in these clones, we built B cell phylogenetic trees for all clones containing at least three sequences that were either distinct or found at different time points (largest shown in Fig. 9c). We observed a relatively high level of SHM in these clones at time point A (mean = 4.7%). We then used a phylogenetic root-to-tip correlation test (see “Methods” section) to determine if divergence from the germline sequence increased between time points A and B in these 20 clones. None showed a significant positive correlation between sample time and divergence from the sequences’ most recent common ancestor (i.e., *p*-value > 0.05; Fig. 9d). These results indicate a lack of measurable SHM accumulation between time points in these clones. Taken together, the observed expansion of unmutated plasmablasts and lack of measurable B cell evolution is consistent with prior work showing evidence of disrupted germinal-center reactions in hospitalized COVID-19 patients^79^.

### Convergent antibody rearrangements are elicited in COVID-19 patients

We identified 19 convergent antibody clusters across eight patients. 79% (15/19) of convergent clusters included antibodies from tocilizumab-stable patients, 47% (9/19) included antibodies from no-tocilizumab-stable patients, and 31% (6/19) of convergent clusters included antibodies from tocilizumab-progressive patients (Fig. 9e). We next focused on the tocilizumab-stable patients, which were represented in the most convergent clusters. We identified six convergent clusters which were composed only of tocilizumab-stable patients (Fig. 9f). These convergent antibody clusters spanned between two patients, and were composed of IGHV1-46/IGHJ3, IGHV3-23/IGHJ6, IGHV3-33/IGHJ6, IGHV3-48/IGHJ3, IGHV4-59/IGHJ6, IGHV5-51/IGHJ4 genes.

Taken together, our multi-omics single-cell analysis revealed dynamic immune responses in patients with a stable and progressive manifestation of COVID-19 under tocilizumab treatment. Our comprehensive immune profiling underscores the overwhelming IFN-I response and desynchronized adaptive and innate immune interaction in COVID-19. Joint profiling of gene expression and surface proteins uncovered hyper IFN-I response in activated T cell subset in progressive patients. Excessive regulatory innate immune response and unique co-inhibitory receptor expression in activated T cells are hallmarks of progressive disease. Skewed T cell repertories in CD8^+^ T cell and uniquely enriched VDJ sequences are identified in COVID-19 patients. Plasmablasts expansion without acquiring somatic hypermutation is consistent with an early primary and/or extrafollicular response during the acute phase of SARS-CoV-2 infection. Lastly, we characterize the cellular response to tocilizumab, including decreased expression of S100A8/9 in tocilizumab-treated patients across most cell types.

## Discussion

Although initial studies in COVID-19 patients with severe respiratory failure revealed a dysregulated immune system with hyper-inflammatory responses and lymphopenia^12–14,80–82^, there are critical deficits in our understanding of the mechanisms underlying this immune dysfunction. Here, we applied multimodal single-cell analysis using 5′ scRNA-seq, CITE-seq, TCR, and BCR sequencing on 18 samples from 10 patients with COVID-19, which were compared to 13 control samples. Our experimental design allowed us to determine: (1) the dynamics of the immune response in COVID-19 over time; (2) general immune cell features in COVID-19 patients; (3) the specific immune signature associated with progressive disease; (4) an in-depth exploration of adaptive immunity using T cell and B cell repertoire analysis in COVID-19; and (5) the effects of tocilizumab treatment. Our unbiased systems biology approach utilizing novel multimodal single-cell analysis techniques reveals the temporal dynamics of immune responses to this disease. It highlights the unique immune features that distinguish stable and progressive COVID-19 patients. The immunological networks characterized in our study improve the understanding of the abundant cellular interactions and effects that promote pathology in severe disease, including dyssynchrony of the innate and adaptive immune response.

We observed dominant effects of a type-1 IFN response across all immune cells in all COVID-19 patients, especially at the earlier time-point A, consistent with the acute viral infection^83^. In this regard, we highlight the IFN-activated CD8^+^ T cell cluster as the extreme archetype of type-1 IFN response in T cells, which was almost exclusively found in COVID-19 patients, especially at time-point A (Fig. 1f). Type-1 IFN response was the main driver of gene expression changes between progressive and stable subjects, as depicted in Fig. 2a. Progressive patients had higher expression of ISGs in all major cell types. Type-1 IFN response, as reflected by the IFN gene score, decreased overwhelmingly from time point A to B in all patients, and was highly correlated with time (*R* = 0.97, Fig. 2f, g). This is not surprising given that the first blood draw was obtained at least 5 days after the beginning of symptoms, the onset of which occurs a median of 5.2 days after infection^84^. At this time-point, patients are expected to be on the descending slope of the viral load curve^30^, which type-1 IFN response closely follows (*R* = 0.8 for the correlation between log_10_ viral load and IFN score, Fig. 2e). Interestingly, the ISG signature at the later time-point B returned towards control level in all patients except for two out of four in the progressive group (Fig. 2a), in which both IFN score and viral load remained relatively high. This is consistent with a previous study in which severe COVID-19 patients had a higher viral load and longer virus shedding than mild cases^85^.

The joint profiling of gene expression and surface proteins shed light on a specific T cell population that expressed higher HLADR, CD38, and cell cycle markers (*MKI67, PCNA, AURKA*) and expanded with time. Although there was no significant difference in frequency noted between progressive vs stable COVID-19 patients in our cohort, we demonstrated the enrichment of ISGs and a unique set of co-inhibitory receptors (LAG-3 and TIM-3, coded by *HAVCR2*) in progressive patients. Interestingly, TIGIT and PD-1 (coded by *PDCD1*) were relatively higher in stable patients together with *TCF7* and *LEF1*, the markers for progenitor exhausted T cells^63^. In general, T cell exhaustion is observed in chronic infectious diseases and cancer where T cells receive sustained stimuli to be activated for long-term in an immunoregulatory milieu^86,87^. In contrast, our observation in severe SARS-CoV-2 infection indicates that a unique activated T cell phenotype can also be induced in acute viral infection^88^, which is not commonly seen. Among signatures observed in progressive patients, elevated *LAG3* expression was detected across most T/NK cell subsets, except for the Treg cluster^16,40,71^. Given that the expression of MHC-II molecules (which are the ligands for LAG-3) is markedly downregulated in APCs in progressive COVID-19 patients, the disruption of LAG3-MHC-II interaction might play a critical role in COVID-19 immunopathology. While the trigger-inducing LAG-3 on T cells is not well understood, co-expression of ISGs in T cells from progressive patients suggests a possible role of IFN-I on LAG-3 expression. Further studies focusing on the molecular mechanisms by which SARS-CoV-2 induces LAG-3 on T cells are warranted.

A skewed phenotype of myeloid cells toward a regulatory/immunosuppressive signature has been previously reported in severe COVID-19^13,14^. In contrast, a pro-inflammatory monocyte phenotype has been shown by others in patients with severe COVID-19^80–82^. Our findings of an anti-inflammatory monocyte cluster that is increased in COVID-19 patients, along with suppressive/tissue-repair gene expression changes in monocytes that are accentuated in progressive patients (including *CD163, IL1R2, AREG, MRC1*, *HAVCR2*, *LGALS9, IL10*), support the former evidence. We also observed the downregulation of IL-1β mRNA expression in COVID-19 patients, indicating a shift from pro-inflammatory to a regulatory phenotype in monocytes in COVID-19. Our multimodal single-cell analysis demonstrated that these regulatory monocytes are transcriptionally distinguished from the other classical monocytes and resemble myeloid-derived suppressor cells (MDSCs), a heterogeneous myeloid cell population characterized by strong immunosuppressive function increased in chronic infection and cancer. The increased fraction of MDSC-like anti-inflammatory monocytes facilitates the resolution of inflammation during acute viral infection, transitioning from inflammation to tissue repair^89,90^. However, an inadequate anti-inflammatory/tissue repair response can delay virus clearance, cause chronic inflammation, and lead to excessive tissue damage and even tissue fibrosis^91–94^. Therefore, a regulated transition of the immune response from inflammation to tissue repair with appropriate kinetics is essential to restore tissue homeostasis.

We observed an increased expression of the suppressive cytokine *IL10* in myeloid cells and several additional cell types in the progressive patients (Supplementary Fig. 8a). Levels of IL-10 in plasma are known to be increased in severe COVID-19, as reported in our recent study^4^ as well as by others^7,31^. While IL-10 is critical to protect the host from tissue damage during acute immune responses, it also exhibits a detrimental immunopathogenic effect during acute viral infections by downregulation of MHC-II expression^92^. Our data clearly demonstrated the upregulation of IL-10 and the downregulation of MHC-II expression in monocytes which may contribute to the detrimental clinical course in progressive patients compared with stable ones.

Our T cell receptor repertoire analysis shows a higher expansion/dominance and a lower richness of CD8^+^ but not CD4^+^ T cell clones in progressive COVID-19 in progressive patients relative to stable patients. In an attempt to identify specific clonotypes that are relevant to T cell response against SARS-CoV-2, we studied the CDR3 motifs that are shared by several COVID-19 patients but absent from the control subjects. Using this approach, we identified 10 CD8^+^ and 12 CD4^+^ T cell specificity groups, and described their specific V & J gene usage patterns. Moreover, we describe the specific CDR3 motifs that have the highest likelihood of being COVID-19 specific. In future experiments, these TCRs will be investigated for antigen specificity.

During viral infection, B cells are critical for the production of protective antibodies. The establishment of a diverse repertoire of antibodies is imperative to protect a host from pathogens, as well as to generate effective immune responses. One key finding is that CDR-H3 amino acid usage profiles were highly variable among patients (Fig. 8g). In particular, there was no preference for any amino acid between early and late time points in stable patients who received tocilizumab. In contrast, progressive and stable patients who were not treated with tocilizumab showed a different profile of CDR-H3 amino acid selection which generally varied across amino acids with a trend toward increased usage of alanine (A), aspartic acid (D), and tyrosine (Y) at time-point B relative to A. The importance of CDR-H3-tyrosine for optimal antibody binding was previously shown for the influenza A virus^95^. Another finding focused on the detection of convergent antibodies with highly similar VDJs across COVID-19 patients (Fig. 8n, o).

Our V(D)J B cell receptor repertoire analysis further suggests a complex B cell response in COVID-19. Consistent with the expectations of a primary immune response, we observed an high proportion of unmutated IGHG B cell clones, which has been reported in at least one other analysis of BCR repertoires from COVID-19 patients^77^. However, we also observed multiple mutated B cell clones that were persistent across the two measures time points, but did not measurably accumulate SHM between time points (Fig. 8l, m)^96^. These could result from cross-reactivity of memory B cells with other common corona-viruses, which has been documented in T cells^86^. Such memory B cells would have already accumulated SHM and likely avoid germinal-center re-entry^97^. This scenario may account for the quick expansion of plasma cells in COVID-19 patients (Fig. 1f), which has also been reported by others^14,60^. It is also possible these are clones are non-coronavirus-specific persistent clones sometimes observed in healthy older patients^98^. Importantly, these analyses were performed over a short time interval, and used a relatively small number of B cells with unknown specificity. We note, however, that the lack of observable SHM increase between time points is consistent with another recent study which found that levels of SHM in SARS-CoV-2-specific antibodies were stable (~3%) between 8 and 42 days post-diagnosis^99^.

The relatively small sample size (18 COVID-19 samples and 13 controls) is a limitation of this study, especially for the analysis of certain subgroups (e.g., only four samples from patients who did not receive tocilizumab). However, it is larger than many COVID-19 single-cell multi-omics studies published to-date^14,80,81,100^, and the similarity of baseline characteristics between stable and progressive patients and in comparison, to controls (Supplementary Table 1) helps increase confidence in our results. Although the timing of blood draw A (time-point A) relative to hospitalization was consistent across subjects, the timing of blood draw B (time-point B) was variable. We mitigated that by taking into account the variable time span between the two blood draws in some of the analyses, e.g., for the analysis of IFN score changes over time shown in Fig. 3a, b. This unique exploration of gene expression changes over time adds an essential dynamic layer that is critical to understand the biology of an acute viral disease, although tocilizumab treatment may have influenced the gene expression changes. However, when comparing the gene expression at time point A and B (Fig. 2a), most differentially expressed genes are related to the strong type-1 interferon response rather than tocilizumab effects. This led us to conclude that the response to the virus (rather than to the treatment) is the main driver of gene expression changes over time. Nonetheless, we devoted a part of the results to explore the effects of tocilizumab by comparing gene expression changes in the untreated patients across time to those of the treated patients, and uncovered genes (in each cell type) whose change is likely to be attributed to tocilizumab effects rather than time. Lastly, our analysis mostly relied on RNA-based analyses including gene expression and TCR/BCR repertoire analysis, with some protein-level validation by CITE-seq and flow cytometry. Additional mechanistic validation, while beyond the scope of this study, is warranted in future studies.

In conclusion, our in-depth multi-omics assessment of peripheral immune cells at single-cell resolution across patient severities and time highlights the desynchronized adaptive and innate immune response in progressive COVID-19 patients. A prominent type-1 interferon response is observed across all immune cells, especially in progressive patients, and wanes over time in correlation to the decrease in viral loads. Excessive regulatory innate immune response and LAG-3 positive activated T cells are the hallmarks of progressive disease. Skewed T cell receptor repertoires in CD8^+^ T cell and uniquely enriched V(D)J sequences are identified in COVID-19 patients. B cell receptor repertoire analysis reveals a high level of IGHG B cell clones with little or no somatic hypermutation, consistent with an early primary and/or extrafollicular immune response, as well as mutated clones which may reflect stimulation of pre-existing memory B cells. Overall, our comprehensive immune profiling underscores the desynchronized innate and adaptive immune interaction in progressive COVID-19, which may lead to delayed virus clearance. This high-resolution understanding of the immune cell profiles underlying severe COVID-19 will enhance our ability to develop immunomodulatory therapeutic approaches to prevent progression in COVID-19 patients.

## Methods

### Settings

The study was performed on deidentified, cryopreserved PBMC samples of 10 COVID-19 patients and 13 matched controls, obtained with informed consent on a protocol approved by Yale Human Research Protection Program Institutional Review Boards (FWA00002571, Protocol ID. 2000027690).

### Patients and samples

Ten COVID-19 patients hospitalized at Yale-New Haven Hospital (YNHH) were recruited for this study. All were confirmed to have COVID-19 by RT-PCR testing of nasopharyngeal samples. Four of the patients were “**P**rogressive” (TP6, TP7, TP8, TP9), defined as patients who required admission to the intensive care unit (ICU) and eventually succumbed to the disease. At the same time, the other 6 were “**S**table” (NS0, NS1, TS2, TS3, TS4, TS5), defined as patients hospitalized in non-ICU internal medicine wards who were eventually discharged. Eight patients (80%) were treated with **T**ocilizumab, a humanized anti-IL6 receptor antibody. Only patients NS0 and NS1 did not receive this drug (designated with “**N**”). Tocilizumab was given once at a dose of 8 mg/kg (up to a maximal dose of 800 mg), as part of the COVID-19 treatment algorithm used in the Yale-New Haven Health System; patients who required ≥3 l/min O_2_ or ≥2 l/min but with CRP > 70 were treated with tocilizumab. All patients were treated with antivirals (Atazanavir, except for patient NS1 which was treated with Remdesivir) and with Hydroxychloroquine (except for NS1). Two progressive subjects were treated with corticosteroids: patient TP7 was treated with Prednisone 40 mg daily for 2–3 days just before the blood draw A, and patient TP9 was treated with Methylprednisolone 120 mg daily for 1–2 days prior to blood draw B. No other immunosuppressive, immunomodulatory, or antiviral agents were used.

Eighteen blood samples were collected from these ten patients, at different time points as described in the results section and Fig. 1b, c. Thirteen control subjects were recruited prior to the COVID-19 pandemic. Baseline characteristics of COVID-19 patients and controls are presented in Supplementary Table 1. The timing of symptom onset, hospitalization, tocilizumab treatment, and blood draws for each patient is shown in Fig. 1d.

### Isolation of PBMC and cryopreservation

PBMCs were isolated from whole blood using density gradient centrifugation, according to the following protocol: Histopaque 20 ml was added to a 50 ml SepMate tube, then overlaid with fresh blood 1:1 diluted in PBS 2% fetal bovine serum (FBS) and centrifuged at 1200×*g* for 10 minutes. The PBMC layer was collected by quickly pouring the remaining contents above the SepMate insert into a fresh tube, and washed once with PBS at 650 × *g* for 10 min. The supernatant was decanted and ACK red blood cell lysis buffer (2 ml/sample) was added for 2 minutes; another wash with PBS 2% FBS was done, followed by centrifugation at 290 × *g* to remove platelets and supernatant aspirated. Following resuspension of the pellet, PBMCs were cryopreserved in aliquots of 5 × 10^6^ cells using 10% DMSO in heat inactivated-FBS as the cryopreservation solution. Cryovials were placed in a freezing container (Mr. Frosty) and transferred immediately to a −80 °C freezer for >24 h before being transferred to long-term liquid nitrogen storage.

### Sample preparation and 10x barcoding

All sample processing steps were done in a biosafety level 2+ laboratory. Samples were thawed in a water bath at 37 °C for ~2 min without agitation, and removed from the water bath when a tiny ice crystal still remains. After thawing, cells were gently transferred to a 50 mL conical tube using a wide-bore pipette tip, the cryovial was rinsed with a cold growth medium (10% FBS in DMEM) to recover leftover cells, and the rinse medium was added dropwise (1 drop per 5 s) to the 50 ml conical tube while gently shaking the tube. Next, we conducted serial dilutions with cold growth medium a total of 5 times by 1:1 volume addition with ~1 min wait between additions. Cold growth medium was added at a speed of 3–5 ml/s, achieving a final volume of 32 ml. The cells were then centrifuged at 300 × *g* for 5 minutes at 4 °C, and the supernatant was removed without disrupting the cell pellet. The pellet was resuspended in 1× PBS with 0.04% BSA, and the sample was filtered with a 40 μM strainer. Cell concentration was determined using Trypan blue staining with a Countess automated cell counter (ThermoFisher). Following this cell count, each sample was split into two parts (Fig. 1a): one was immediately loaded onto the 10x Chromium Next GEM Chip G, according to the manufacturer’s user guide (document number CG000208, revision E, February 2020), and the other was further processed for CITE-seq as described in the next section, and then loaded to the 10x Chromium Chip G. In total, we loaded 18 “conventional” samples into 18 Chip G lanes (aiming for recovery of 10,000 cells per lane), and 17 out of 18 “CITE-seq” samples into 6 Chip G lanes (each lane containing 5-6 pooled hashed samples, as portrayed in Supplementary Data 6). One out of 18 CITE-seq samples (TP8B) was not pooled because of very low cell concentration.

### CITE-seq and cell hashing

The lyophilized Total-seq C human panel (BioLegend) was resuspended with 35 μl of wash buffer, vortexed for 10 s, and incubated for 5 min at RT. Total-seq C human Hashtag antibodies (Biolegend) were centrifuged at 20,000 × *g* for 10 min and 6-fold diluted with wash buffer (2% FBS and 1 mM EDTA in PBS). To maximize performance, both were centrifuged at 20,000 × *g* for 10 min just before adding to the cells. See Supplementary Data 5 for a list of antibodies, clones, and barcodes used for CITE-seq and hashing samples.

PBMCs from each sample were reconstituted with wash buffer at the concentration of 10–20 × 10^6^ cells/ml and incubated on ice for 10 min with 5ul of Human F_C_ block (BD Biosciences) and 5 μl of TrueStain Monocyte Blocker (Biolegend). 10–20 μl (0.1–0.2 × 10^6^ cells) were transferred into a new tube and incubated on ice for 30 min with 5 μl of CITE-seq panels and 5 μl of Hashtag antibodies prepared as above. Cells were washed twice with wash buffer and with 2% FBS in PBS for the third wash. Samples were pooled into one tube based on cell counts, and super-loaded onto the 10× Chromium Chip G, aiming for recovery of ~20,000 cells per sample. See Supplementary Data 5 for the details of 6 pooled samples (CITE#1-CITE#6).

### cDNA libraries preparation and sequencing

The loaded Chip G was placed in the 10x Chromium controller to create Gel Beads-in-emulsion (GEMs). The next steps were carried out according to the manufacturer’s user guide, including GEM-RT incubation, post-GEM-RT Dynabead cleanup, and cDNA amplification. The cDNA samples were used to construct 4 types of cDNA libraries, according to the steps outlined in the user guide: gene expression libraries, T-cell receptor libraries, B-cell receptor libraries, and cell surface protein libraries (the latter only for samples processed with CITE-seq). cDNA libraries were then sequenced on an Illumina Novaseq 6000 platform.

### Flow cytometry

Freshly isolated PBMCs were incubated with F_C_ block reagent (Biolegend) for 10 min and stained with LIVE/DEAD Fixable Aqua Dead Cell Stain kit (ThermoFisher) for 20 min at 4 °C. Following a wash, cells were then blocked with Human TruStan F_C_X (BioLegend) for 10 min at RT. Cocktails of the following antibodies were directly added to this mixture for 30 min at RT. BB515 anti-HLA-DR (G46-6), BV605 anti-CD3 (UCHT1), BV785 anti-CD4 (SK3), APCFire750 anti-CD8 (SK1), BV421 anti-CCR7 (G043H7), AlexaFluor 700 anti-CD45RA (HI100), PE anti-PD1 (EH12.2H7), APC anti-TIM3 (F38-2E2), BV711 anti-CD38 (HIT2), BB700 anti-CXCR5 (RF8B2), PE-Cy7 anti-CD127 (HIL-7R-M21), PE-CF594 anti-CD25 (BC96), BV711 anti-CD127 (HIL-7R-M21), BV421 anti-LAG-3 (11C3C65). Cells were washed two times with staining buffer and acquired on a BD Fortessa or Cytoflex flow cytometer. FlowJo software (Treestar) was used for analysis.

### SARS-CoV-2 viral load measurements

Nasopharyngeal swabs and saliva samples were collected from COVID-19 diagnosed inpatients at -Yale-New Haven Hospital, as described elsewhere^101^. We extracted total nucleic acid using the MagMax Viral/Pathogen Nucleic Acid Isolation kit (ThermoFisher Scientific, Waltham, MA, USA) with 300 µl of input sample eluted into 75 µl, using a slightly modified protocol (dx.doi.org/10.17504/protocols.io.bg3pjymn). A total of 5 µl of extracted nucleic acid was used as input in the RT-qPCR assay for SARS-CoV-2 detection, as described elsewhere^102^. Briefly, we used the Luna Universal Probe One-Step RT-qPCR kit (New England Biolabs, Ipswich, MA, USA) with the CDC 2019-nCoV_N1, 2019-nCoV_N2, and human RNase P (RP) primer-probe sets (Integrated DNA Technologies, Coralville, IA, USA). Viral RNA copy numbers were calculated based on 10-fold dilution standard curves of the previously generated nucleocapsid (N) transcript standard^102^.

### Data processing of raw sequencing reads

Raw sequencing reads were demultiplexed using Cell Ranger mkfastq pipeline to create FASTQ files. Next, Cell Ranger count pipeline (v3.1) was employed in order to perform alignment (using STAR), filtering, barcode counting, and UMI counting. We have used GRCh38 (Ensembl 93) as the genome reference (corresponding to Cell Ranger reference GRCh38-3.0.0).

### ScRNA-seq sample aggregation

10× cell ranger count filtered output data of PBMCs from thirteen healthy controls were added to that of the eighteen COVID-19 samples. Seurat package^103,104^ (v3.1) was used for all downstream analyses. 10× gene expression matrices for each sample were converted and combined into one Seurat object. Cells with mitochondrial gene percentages higher than 12% and cells with less than 200 genes were excluded from the study to filter out dead and dying cells. For CITE-seq samples, following de-hashing, cell barcodes of multiplets (i.e., with 2 or more hashing antibody signals) or uncertain origin (i.e., with no clear hashing signal) were also removed. After these filtering steps, the gene-barcode matrix contained 35,538 genes and 163,452 barcoded cells.

### Integration, principal components analysis, and clustering

In accordance with the standard Seurat preprocessing workflow, sample gene expressions were normalized using Seurat’s “*LogNormalize*” method^103,104^. The “*FindVariableFeatures*” function selected the 3000 genes with the highest variance to mean ratio using the “*vst*” method. To remove single-subject effects, samples were integrated on a subject level using 2000 anchors with a dimensionality of 30^104^. The integrated data were then scaled with the “*ScaleData*” function.

Principal Component Analysis (PCA) was performed on the integrated data, and the first 30 Principal Components (PCs) were used in the “*FindNeighbors*” algorithm. The Louvain modularity optimization algorithm in “*FindClusters*” generated the clusters while the resolution was set to 0.75. Thirty PCs were used in the “*RunUMAP*” function to create the final UMAP, and thirty clusters were generated from the aforementioned pipeline (Supplementary Fig. 2a).

These thirty clusters were first annotated with the SingleR software (Supplementary Fig. 2d) and then annotated manually (Fig. 1e) by using cell-specific markers (Supplementary Fig. 1) plotted on UMAP space, and by examining the output of “*FindAllMarkers*” per cluster. Five clusters out of thirty were removed; namely: a nonspecific cluster of low UMI cells (cluster #8), monocyte-platelet multiplets (#22), B and T/NK multiplets (#24), erythroid cell contamination from a single subject (#25), and B cell-platelet multiplets (#29). Following the removal of these clusters, the final Seurat object contained 153,554 cells.

### Cell type proportions analysis

For each subject, the number of cells within a given cell type was normalized by the subject’s total number of cells. For each cell type, cell proportions were plotted in a boxplot by disease group, namely by (1) controls, (2) stable patients at time point A, (3) stable patients at time point B, (4) progressive patients at time point A, and (5) progressive patients at time point B. We used the Wilcoxon rank-sum test to compare the cell proportions of the following three groups: control subjects, stable patients, and progressive patients.

### Differential gene expression analysis

The “FindMarkers” function was used to identify differentially expressed genes (DEGs) across the following conditions: per cell type, (1) time point A versus time point B; (2) controls versus COVID-19 patients at time point A; and (3) progressive vs stable. For the time point comparison (comparison 1), the logistic regression test for differential expression with subjects set as latent variables was used to account for paired samples. For comparisons (2) and (3), the default Wilcoxon rank test was used. Genes were ranked by absolute log2 fold-change (logFC), and those with *p*-values > 0.05 (adjusted for multiple comparisons) were removed.

### Heatmap visualization of DEGs

DEGs were visualized as heatmaps which were generated by using the ComplexHeatmap package^105^. Cell types were binned into monocytes, CD4^+^ T cells, CD8^+^ T cells, and B cells, and “FindMarkers” distinguished DEGs for each cell type bin for (1) time point A versus time point B and (2) progressive versus stable. Genes with greater than 0.5 absolute logFC were included in visualization and EnrichR pathway analysis. Samples for the progressive versus stable time-point were hierarchically clustered.

### Gene pathway annotation

Gene list outputs from the “FindMarkers” function were fed into EnrichR for pathway and ontology analysis^106,107^. Gene set enrichment analysis^108^ was also performed on “Dividing T cells” cluster using KEGG^109–111^ and MSigDB Hallmark gene sets^112^, and custom gene sets (Supplementary Data 8).

### Gene list score analysis

Seurat Function “AddModuleScore” was used to combine the expression of genes from IFN Score A^113^ (*ISG15, IFI44, IFI27, CXCL10, RSAD2, IFIT1, IFI44L, CCL8, XAF1, GBP1, IRF7, CEACAM1*). This function was also used to combine other gene list scores as well, including scores for HLA type II (*HLA-DRA, HLA-DQA1, HLA-DPA1, HLA-DRB1, HLA-DPB1, HLA-DRB5, HLA-DQB1, HLA-DMA, HLA-DMB*) and the IL6 pathway (*ARID5A, SOCS3, PIM1, BCL3, BATF, MYC*)^49^. The differences in gene list scores were compared between (1) control versus COVID-19 patients, (2) time point A and time point B, and (3) progressive versus stable patients. For consistency with DEG analysis and assumption of non-normality, Wilcoxon rank-sum tests were conducted on the gene set “module scores” to compare between any two given conditions and corrected for multiple comparisons (i.e., family-wise error rate (FWER)).

### Demultiplexing (de-hashing) of CITE-seq samples

In order to demultiplex cells in the CITE-seq samples and attribute them a biological sample, hashing antibody-derived tag (ADT) counts were normalized by library size, square-root transformed, and normalized for every row in the data matrix of each CITE-seq sample. To account for the inherent background noise of ADT and accurately identify a cell as tagged by a hashing ADT, histograms of each hashing ADT counts in each CITE-seq sample were used to determine the optimal threshold of significance for hashing ADTs. As distributions appeared bimodal for the majority of hashing, we manually set the threshold between the two modes.

Based on the previous threshold, data matrix rows with two or more significant ADT were flagged as doublets, and rows with zero significant ADT flagged as unidentified, thus removed for downstream analysis.

### CITE-seq ADT preprocessing and downstream analysis

Once the cells were demultiplexed and hashing ADT counts were removed, the remaining ADT counts (192) for each CITE-seq sample were combined into one single matrix. The counts for the remaining 43,349 cells were normalized by library size and square root transformed. We visualized the data set using Uniform Manifold Approximation and Projection (UMAP). Cells were clustered using the Louvain community detection on a 15-nearest neighborhood graph and were manually annotated using a panel of ADT markers for each cell type (cell types include: CD4^+^ T cells, CD8^+^ T cells, B cells, NK cells, monocytes, macrophages, DCs, plasma cells, neutrophils, eosinophils, platelets, and red blood cells). We also manually annotated the clusters based on surface marker lists proposed by the Human Immunology Project Consortium (HIPC) (https://www.immuneprofiling.org) (Supplementary Data 7). As gene expression (GEX) data from CITE-seq was incorporated in the standard scRNA-seq analysis, there were two different annotations: one based on GEX, and one based on ADT. To measure the concordance between the two annotations, the percentage of shared cells between each annotated cluster was computed (Supplementary Fig. 14b).

Differential expression analysis was performed using the Wilcoxon rank-sum test, and *p*-values were adjusted for multiple hypothesis testing using the Benjamini–Hochberg correction. Data preprocessing and analysis (for ADT analysis only) was performed in Python (version 3.8.0) using Scanpy (version 1.4.6)^114^.

### Differential connectivity (connectomic) analysis

For connectomic analysis, the cell parcellation shown in Fig. 1e was used except for IFN-activated CD8^+^ T cells, which were lumped into the Effector T cell cluster. These data were then mapped against a version of the FANTOM5 database of ligand–receptor interactions, modified to include additional immunomodulatory cues of interest to the authors (Supplementary Data 9). Each parcellation, in a given experimental condition, was then treated as a single signaling node for network analysis. Average expression values were calculated for all ligand and receptor genes on a per-cell-type basis. Then an unfiltered edgelist (connectome) was created linking all producers of a ligand to all producers of a receptor, with associated quantitative edge attributes, as previously described.

To compare experimental conditions, the connectomes from two experimental conditions were directly compared to yield log-fold changes for the sending (ligand) side and receiving (receptor) side of all edges. In addition, a “perturbation score” was calculated, which allows plotting of differential edges proportional to the degree of change, allowing both negative and positive log-fold changes and incorporating information from both sides of a given edge. The perturbation score that we used was defined, for every cell vector from Cell_*i*_ to Cell_*j*_ for ligand–receptor mechanism (*k*), as:

$${{{{{{{\mathrm{score}}}}}}}}^{{ijk}}=\left|{\log }\left(\frac{{{{{{{{\mathrm{Cell}}}}}}}}_{i}^{{L}_{k}^{{{{{{{\mathrm{test}}}}}}}}}-{{{{{{{\mathrm{Cell}}}}}}}}_{i}^{{L}_{k}^{{{{{{{\mathrm{control}}}}}}}}}}{{{{{{{{\mathrm{Cell}}}}}}}}_{i}^{{L}_{k}^{{{{{{{\mathrm{control}}}}}}}}}}\right)\right|\times \left|{\log }\left(\frac{{{{{{{{\mathrm{Cell}}}}}}}}_{j}^{{R}_{k}^{{{{{{{\mathrm{test}}}}}}}}}-{{{{{{{\mathrm{Cell}}}}}}}}_{j}^{{R}_{k}^{{{{{{{\mathrm{control}}}}}}}}}}{{{{{{{{\mathrm{Cell}}}}}}}}_{j}^{{R}_{k}^{{{{{{{\mathrm{control}}}}}}}}}}\right)\right|$$

Edges were then plotted which (1) had >10% of the sending and receiving cluster expressing the given ligand and receptor, respectively, and (2) which displayed an adjusted *p*-value < 0.05 via a Wilcoxon Rank-Sum test comparing identical cell types to each other across experimental conditions.

Chord diagram plotting was performed using a custom implementation of the *circlize* package with directed edge thickness between cell type nodes proportional to the above-described perturbation score, scaled per-plot. The software used for the connectomic analysis is available at https://github.com/msraredon/Connectome (10.5281/zenodo.5574620) (ref:10.1101/2021.01.21.427529v1).

### Tocilizumab treatment effect analysis

To investigate the treatment effects of tocilizumab on transcription levels for different cell types, we conducted differential expression analysis between the two sampling time points for patients in the tocilizumab treatment group and those in the non-tocilizumab group separately. The logFC from these two separate analyses, i.e., for the tocilizumab group and non-tocilizumab group, was scatter plotted for each cell type in order to identify genes in which the differential expression pattern observed between the two-time points is due to a treatment effect rather than the natural course of the disease progression (Fig. 5c). In addition, we investigated the correlations across cell types and compared results between the tocilizumab and non-tocilizumab groups (Supplementary Fig. 13). Six IL-6 pathway-related genes, which are known to be associated with tocilizumab treatment^49^, are highlighted in red (Fig. 5c and Supplementary Fig. 13). All the entries in the heatmap matrix (Fig. 5e) are the differences in logFC between tocilizumab and non-tocilizumab groups.

### T cell receptor V(D)J data processing

The raw sequencing reads of the T cell receptor (TCR) libraries were processed using the Cell Ranger V(D)J pipeline by 10x Genomics™, which assembled read-pairs into V(D)J contigs for each cell, identified cell barcodes from targeted cells, annotated the assembled contigs with V(D)J segment labels and located the CDR3 regions. We only considered V(D)J contigs with high confidence defined by cell ranger under the default settings for downstream analysis. Contigs that were not recognized as either alpha chain or beta chain and cells with no beta chains were removed. Only the alpha and beta chains with the largest UMI count were kept for cells with more than one alpha and/or beta chains. After the filtering, each cell has only one beta chain contig and zero or one alpha chain contig.

The data were further examined and processed for sample-to-sample contamination and potential cell doublets. First, we removed cells with cell barcodes found in more than 2 samples. Second, cells barcodes overlapped between TCR and BCR data were extracted and checked for their cell types determined based on the scRNA-seq gene expression data. Only cell barcodes from T cells were kept. Finally, we checked the gene expression-based cell types of all cells, and cells without an assigned cell type or not belonging to the T cell category were removed. The T cell category includes 13 cell types: Naive CD4^+^ T, Tregs, Naive CD8^+^ T, Effector T, NK CD56dim, Memory CD4^+^ T, NK CD56bright, Dividing T & NK, Memory CD8^+^ T, Dying T & NK, Memory CD4^+^ & MAIT, Gamma-delta T, and IFN-activated CD8^+^ T.

### TCR clone Identification

Before defining clones, we re-annotated the contigs using Change-O^70^. A TCR clone was defined as a group of cells sharing an identical nucleic acid sequence of TCR alpha chain and beta chain in the repertoire of the same subject.

### Specificity group identification by GLIPH2

It was observed that antigen-specific pools of TCRs were enriched for similar CDR3 sequences^115^. To identify clone clusters of TCRs with a high probability of sharing antigen specificity (specificity groups), we applied GLIPH2^72^ to cluster CD4^+^ and CD8^+^ TCR clones from all samples. Clones from the same cluster are predicted to bind the same antigen. Significant clonal groups reported by GLIPH2 were identified based on either local motif-based similarity (shared CDR3 amino acid motifs are comparatively rare in a reference population of naive T-cells) or global similarity (CDR3 differing by up to one amino acid). GLIPH2 assesses the quality of clusters by their global/local similarities, cluster size, and enrichment of common V-genes, a limited CDR3 length distribution, and clonally-expanded clones. The confidence of identified clusters was examined by Fisher’s exact test, which tests for the enrichment of unique CDR3s in each cluster compared to the reference naive CD4^+^ and CD8^+^ T cell repertoire provided in GLIPH2. The V and J gene usage was calculated as the frequency of clones with the corresponding genes in a given clone cluster.

### B cell receptor V(D)J data processing

B cell receptor (BCR) V(D)J repertoire data processing and analysis were carried out using tools in the Immcantation framework (www.immcantation.org). V(D)J genes were re-assigned from CellRanger output using IgBLAST v.1.15.0. Cells with multiple IGH V(D)J sequences were assigned to the most abundant IGH V(D)J sequence by UMI count. Following V(D)J gene annotation, non-functional sequences were removed from further analysis, and functional V(D)J sequences were assigned into clonal groups using Change-O v.1.0.0. Sequences were first partitioned based on common IGHV gene annotations, IGHJ gene annotations, and junction lengths (the junction region is defined as the complementarity-determining region-3 plus the conserved flanking amino acid residues). Within these groups, sequences differing from one another by a length normalized Hamming distance of 0.15 within the junction region were defined as clones by single-linkage clustering^116^ using the DefineClones function from Change-O v.1.0.0 package. This distance threshold was determined at an equal distance between the two modes of the within-sample bimodal distance-to-nearest histogram across all patients. The distance-to-nearest distribution was calculated using distToNearest function from SHazaM v.1.0.0 in R v.3.6.3. Germline sequences were then reconstructed for each sequence with D segment and N/P regions masked (replaced with “N” nucleotides) using the CreateGermlines.py function within Change-O v.1.0.0. The IMGT/GENE-DB v3.1.26 reference database was used to assign B cell gene segments.

### Expanded B cell clonal lineages identification

We identified expanded clonal lineages based on the fractional abundance of each lineage. The fractional abundance of a lineage is defined as the number of cells within that lineage divided by the total number of cells observed in the repertoire at a given time point. Expanded lineages were identified among lineages with fractional abundance above 1% of the repertoire at either time point. To account for the low sequencing depth, we further required expanded clones to contain at least 5 cells.

### Analysis of somatic hypermutation (SHM) from single-cell V(D)J library

Mutations in IGHV and IGHJ relative to germline sequences were quantified using SHazaM v.1.0.0 in R v.3.6.3.

### CDR3 amino acids information content

For a given patient we computed the frequency of observed amino acids in the CDR-H3 segment for each time point A and B. Then, the fold changes were calculated as the log2 ratio of each amino acid frequency at B divided by the corresponding amino acid frequency at A. Finally, each full change was multiplied by the frequency of amino acid at B to calculate the conditional information content of the given amino acid.

### Convergent antibody identification

To identify putative SARS-CoV-2-specific antibody signatures, we first grouped together heavy chain sequences that utilized the same IGHV and IGHJ gene, and had CDR-H3 regions with the same length. We then grouped these sequences using single-linkage clustering with a threshold of 85% amino acid identity in the CDR-H3 sequence. Within these clusters, we identified sequences that were found in at least two COVID-19 patients.

### Identification of unmutated IGHG clones

As specified in a recent study^77^, B cell clones consisting of any cellular subtype (naive, memory, plasma) were separated by isotype. These isotype-specific clonal clusters were considered “unmutated” if the median SHM frequency of their constituent sequences was <1%.

### Lineage tree analysis

B cell lineage trees were built for all clones found at both time points using IgPhyML v1.1.3^117^ and Change-O v1.0.0^70^. Within each time-point, identical sequences and those differing only by ambiguous characters (e.g., “N”) were collapsed. Only clones containing at least three distinct sequences (i.e., sequences that were either unique or sampled at different time points) were included. We estimated maximum likelihood tree topologies and branch lengths for each clone, as well as repertoire-wide model parameters, shared among all clones, using the GY94 model^118^. Using these tree topologies, we then estimated maximum likelihood branch lengths for each clone and repertoire-wide substitution model parameters using the HLP19 model with separate ω parameters for FWR and CDR partitions and separate *h* parameters for all six canonical somatic hypermutation (SHM) hot- and cold-spot motifs^117^. Branches with lengths < 0.001 were collapsed to zero. Trees were visualized using ggtree v2.0.2^119^. We used a root-to-tip correlation test^120^ to test for evidence of continued SHM between time points within these B cell lineage trees. For each tip we calculated the divergence, which is the sum of branch lengths leading to the most recent common ancestor (MRCA) of all observed sequences. Predicted germline sequences were excluded because their sampling time is unknown. Clones in which all sequences were equally diverged from the MRCA were discarded. We then calculated the Pearson correlation coefficient between divergence and time point (*A* = 0, *B* = 1). If B cell clones continued to accumulate SHM between time points, we would expect a positive correlation between divergence and time. We tested the significance of this correlation by randomizing time point labels within each tree, re-calculating the correlation between divergence and time, and repeating for 10,000 repetitions. We calculated the *p*-value that the correlation was positive as the proportion of repetitions in which the observed correlation was less than or equal to the correlation in randomized trees.

### Reporting summary

Further information on research design is available in the Nature Research Reporting Summary linked to this article.

## Supplementary information

Supplementary Information

Description of Additional Supplementary Files

Supplementary Data 1–10

Reporting Summary

## Data Availability

Our data have been deposited in the GEO database under accession code GSE155224. The results can be further explored through the COVID-19 Cell Atlas Data Mining Site (www.covidcellatlas.com). This user-friendly site has a graphical user interface for quick visualization of our scRNA-seq data, which allows users to (1) explore the expression levels of single genes or gene sets of interest across all cell types and (2) conduct comparisons of COVID-19 vs controls, progressive vs stable patients, and early vs late time points across all immune cells in our data set. Source data are provided with this paper.

## Source data

Source Data

## Code availability

The software used for the connectomic analysis is available at https://github.com/msraredon/Connectome. (10.5281/zenodo.5574620) (ref: 10.1101/2021.01.21.427529v1).
