# Supplementary material for: Single-cell multi-omics reveals dyssynchrony of the innate and adaptive immune system in progressive COVID-19
Source: Nat Commun. 2022 Jan 21;13:440. doi: 10.1038/s41467-021-27716-4 (PMC8782894; doi:10.1038/s41467-021-27716-4)
Supplement: Supplementary file 1 — Supplementary Information [file 41467_2021_27716_MOESM1_ESM.pdf]

Supplementary Information

Single-Cell Multi-Omics Reveals Dyssynchrony of the Innate and Adaptive Immune System in Progressive COVID-19

Avraham Unterman*[,1,2], Tomokazu S. Sumida*[,3,4], Nima Nouri[5,6,7], Xiting Yan[1,8], Amy Y. Zhao[1,9,10], Victor Gasque[11,12], Jonas C. Schupp[1,13], Hiromitsu Asashima[3,4], Yunqing Liu[8], Carlos Cosme Jr.[1], Wenxuan Deng[8], Ming Chen[8], Micha Sam Brickman Raredon[1,14,15], Kenneth Hoehn[5], Guilin Wang[16], Zuoheng Wang[8], Giuseppe DeIuliis[1], Neal G. Ravindra[11,12], Ningshan Li[8,17], Christopher Castaldi[18], Patrick Wong[4], John Fournier[19], Santos Bermejo[1], Lokesh Sharma[1], Arnau Casanovas-Massana[20], Chantal B.F. Vogels[20], Anne L. Wyllie[20], Nathan D. Grubaugh[20], Anthony Melillo[5], Hailong Meng[5], Yan Stein[2], Maksym Minasyan[1], Subhasis Mohanty[21], William E. Ruff[3,4], Inessa Cohen[3,4], Khadir Raddassi[3,4], the Yale IMPACT research team, Laura E. Niklason[22], Albert I. Ko[20], Ruth R. Montgomery[10], Shelli F. Farhadian[3, 21], Akiko Iwasaki[4,23], Albert C. Shaw[21], David van Dijk[11,12], Hongyu Zhao[8,9,17,24], Steven H. Kleinstein[4,5,24], David A. Hafler**[,3,4], Naftali Kaminski**[,1], Charles S. Dela Cruz**[,1,25]

* Corresponding authors; ** These authors jointly supervised this work

Affiliations

1. Section of Pulmonary, Critical Care and Sleep Medicine Section, Department of Internal Medicine, School of Medicine, Yale University, New Haven, CT, USA.
2. Pulmonary Institute, Tel Aviv Sourasky Medical Center & Tel Aviv University, Tel Aviv, Israel.
3. Department of Neurology, School of Medicine, Yale University, New Haven, CT, USA.
4. Department of Immunobiology, School of Medicine, Yale University, New Haven, CT, USA.
5. Department of Pathology, Yale School of Medicine, New Haven, CT, USA.
6. Center for Medical Informatics, Yale School of Medicine, New Haven, CT, USA.
7. The Jackson Laboratory for Genomic Medicine, Farmington, CT, USA.
8. Department of Biostatistics, Yale School of Public Health, Yale University, New Haven, CT, USA.
9. Department of Genetics, Yale School of Medicine, New Haven, CT, USA.
10. Department of Internal Medicine, Yale School of Medicine, New Haven, CT, USA.
11. Department of Computer Science, Yale University, New Haven, CT, USA.
12. Cardiovascular Research Center, Section of Cardiovascular Medicine, Department of Internal Medicine, Yale School of Medicine, New Haven, CT, USA.
13. Department of Respiratory Medicine, Hannover Medical School and Biomedical Research in End-stage and Obstructive Lung Disease Hannover, German Lung Research Center (DZL), Hannover, Germany

14. Department of Biomedical Engineering, Yale University, New Haven, CT, USA.
15. Medical Scientist Training Program, Yale School of Medicine, New Haven, CT, USA.
16. Yale Center for Genome Analysis/Keck Biotechnology Resource Laboratory, Department of Molecular Biophysics and Biochemistry, Yale School of Medicine, New Haven, CT, USA.
17. SJTU-Yale Joint Center for Biostatistics and Data Science, Department of Bioinformatics and Biostatistics, School of Life Sciences and Biotechnology, Shanghai Jiao Tong University, Shanghai, China.
18. Yale Center for Genome Analysis, Yale School of Medicine, New Haven, CT, USA.
19. School of Medicine, Yale University, New Haven, CT, USA.
20. Department of Epidemiology of Microbial Diseases, Yale School of Public Health, New Haven, CT, USA.
21. Section of Infectious Diseases, Department of Internal Medicine, Yale School of Medicine, Yale University, New Haven, CT, USA.
22. Departments of Anesthesiology & Biomedical Engineering, Yale University, New Haven, CT, USA.
23. Howard Hughes Medical Institute, Chevy Chase, MD, USA.
24. Inter-Departmental Program in Computational Biology and Bioinformatics, Yale University, New Haven, CT, USA.
25. West Haven Veterans Affair Medical Center, West Haven, CT, USA

Corresponding authors

Avraham Unterman, MD, MBA. Director, Pulmonary Fibrosis Service, Pulmonary Institute, Tel Aviv Sourasky Medical Center, Tel Aviv University. Former Instructor, Section of Pulmonary, Critical Care and Sleep Medicine, Department of Internal Medicine, Yale School of Medicine. 6 Weizmann Street, Tel Aviv, Israel, 6423906. Phone: 972 3 697-4476, Email: ramiu@tlvmc.gov.il.

Tomokazu S. Sumida, MD, PhD. Assistant Professor, Department of Neurology, Yale School of Medicine, 300 George Street, Room 349, New Haven, CT, 06511. Phone: 203 785-6351, Email: tomokazu.sumida@yale.edu.

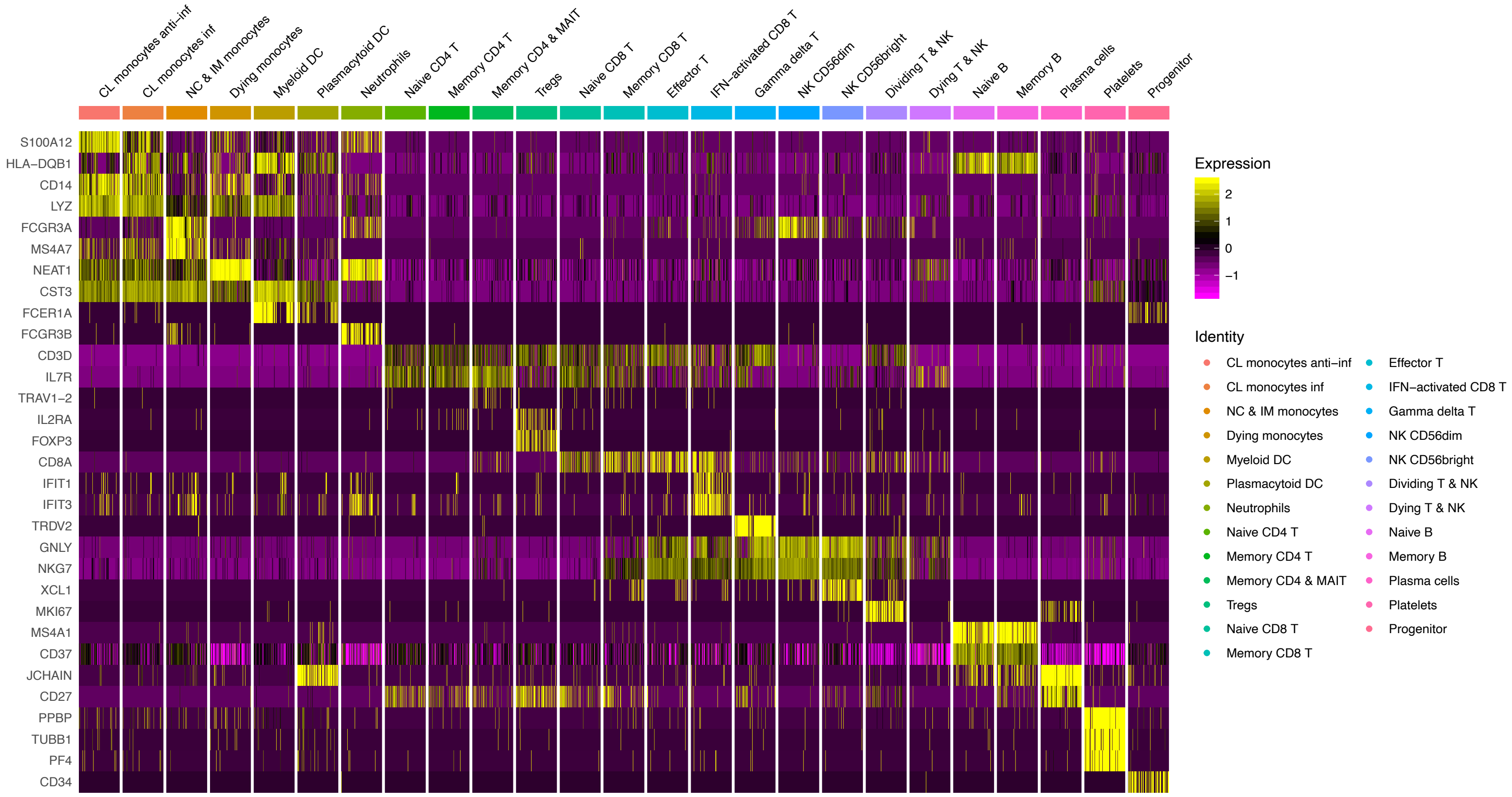

Supplementary Figure 1 Heatmap of main markers used for manual cell type annotation
Shown is a heat map displaying the RNA expression of each of the indicated genes in all the specified cell types. These genes were used as markers to annotate cell types in all samples.

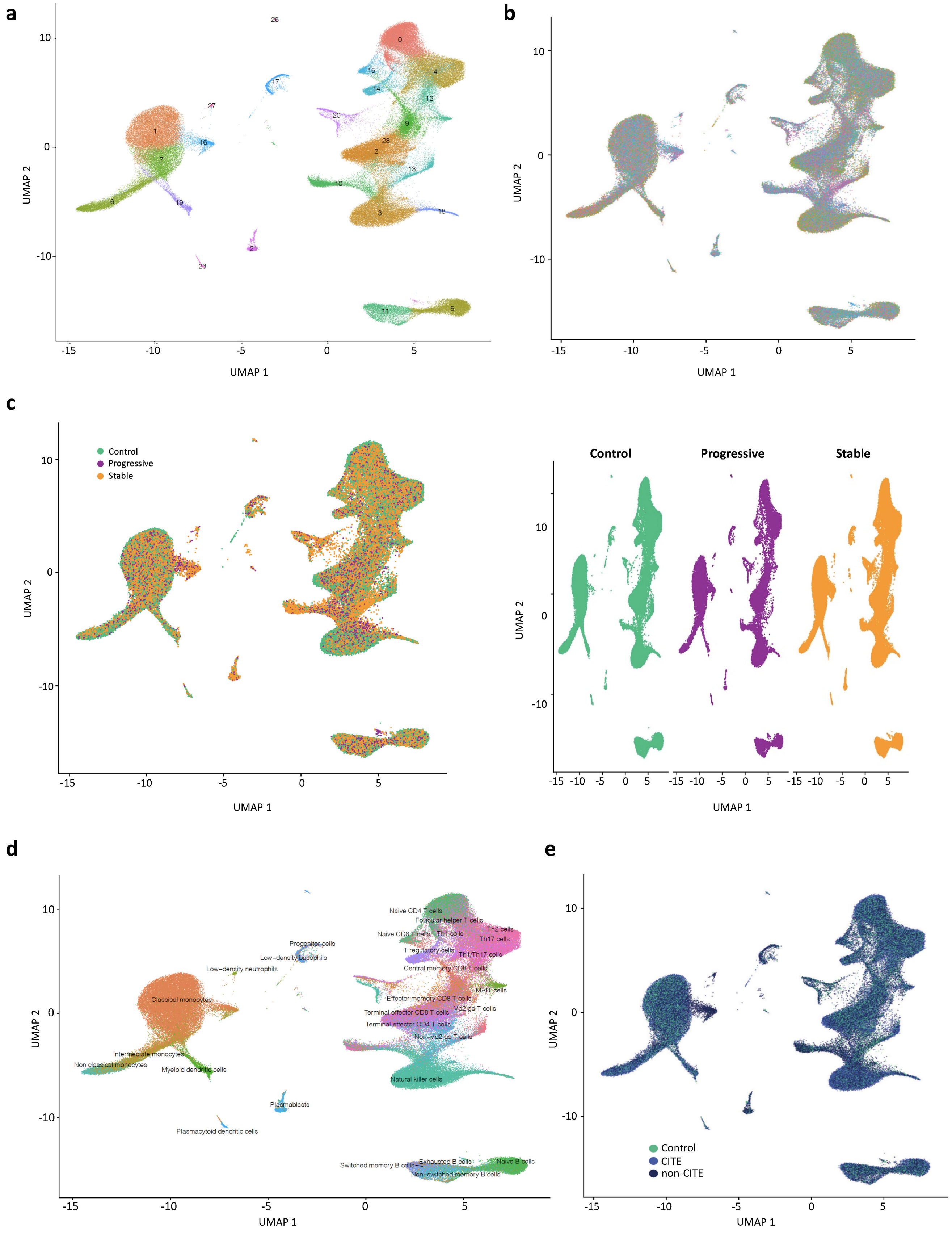

Supplementary Figure 2 Additional UMAPs

a UMAP of Seurat clusters numbered from largest to smallest. Five clusters out of 30 were removed (see Methods for details).

b-c UMAPs demonstrating good overlap between different samples (b) and subgroups (c).

d Automated annotation results using “Single R” package. e Good overlap was also noted between cells processed with and without CITE-seq, except for the dying monocytes cluster (#16) which was reduced in CITE samples.

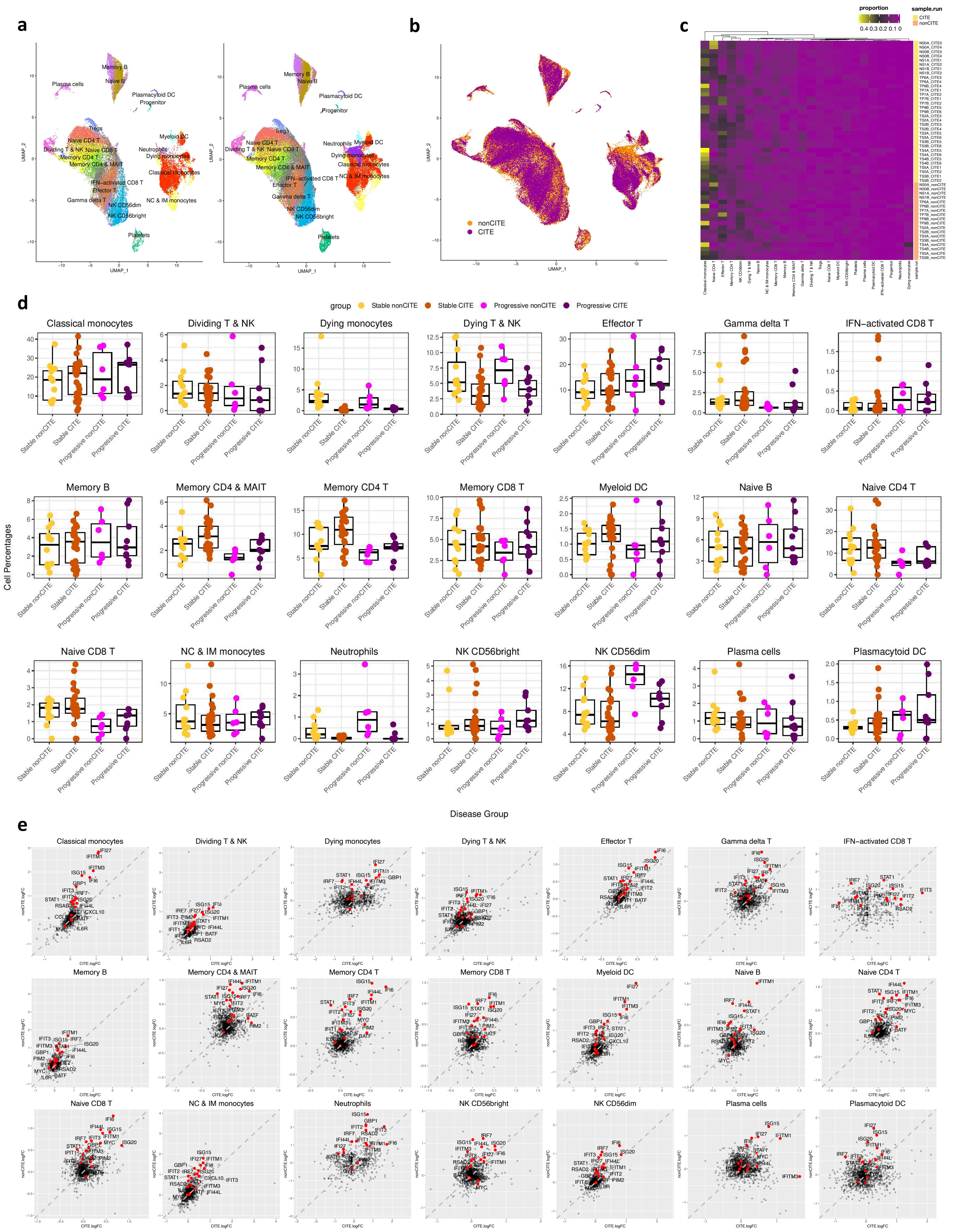

a
Memory B
Naive B
Plasma cells
Plasmacytoid DC
Progenitor
Tregs
Naive CD4 T
Dividing T & NK
Naive CD8 T
Memory CD4 T
Memory CD4 & MAIT
Myeloid DC
Neutrophils
Dying monocytes
Classical monocytes
IFN−activated CD8 T
Effector T
Gamma delta T
NK CD56dim
NK CD56bright
NC & IM monocytes
Platelets
UMAP_1
UMAP_2
b
nonCITE
CITE
c
proportion
sample.run
d
group
Stable nonCITE
Stable CITE
Progressive nonCITE
Progressive CITE
Classical monocytes
Dividing T & NK
Dying monocytes
Dying T & NK
Effector T
Gamma delta T
IFN−activated CD8 T
Memory B
Memory CD4 & MAIT
Memory CD4 T
Memory CD8 T
Myeloid DC
Naive B
Naive CD4 T
Naive CD8 T
NC & IM monocytes
Neutrophils
NK CD56bright
NK CD56dim
Plasma cells
Plasmacytoid DC
Cell Percentages
Disease Group
e
CITE.logFC
nonCITE.logFC

Supplementary Figure 3 Comparison of the two sample processing methodologies: CITE-seq and conventional (non-CITE)

a UMAP embedding without data integration comparing between CITE-seq (left) and scRNA-seq (right) grouped by cell types in a separate view, suggesting limited platform effects.

b UMAP embedding grouped by platforms, i.e. CITE-seq and conventional scRNA-seq (nonCITE), with substantial overlap.

c Heatmap for cell type proportions in each run. The color pattern represents the difference across samples and platforms.

d Boxplots for cell type proportion comparisons across four groups: stable subjects by conventional scRNA-seq, stable subjects by CITE-seq, progressive subjects by conventional scRNA-seq, and progressive subjects by CITE-seq. Showing cell type proportion variations between progressive and stable subjects and between two platforms. The results are depicted in boxplots, in which the upper and lower bounds represent the 75% and 25% percentiles, respectively, the bars inside the boxplots denoting the medians, the whiskers denoting values up to 1.5 interquartile range (IQR) above the 75% percentile or below the 25% percentile. N = 12 for Stable nonCITE, 24 for Stable CITE, 6 for Progressive nonCITE, and 9 for Progressive CITE.

e Gene expression profile in CITE vs nonCITE for each cell type. Dots in grey are the top 2000 highly variable genes between progressive and stable individuals. ISGs and IL-6 pathway genes are highlighted in red. The grey dashed lines represent Y=X.

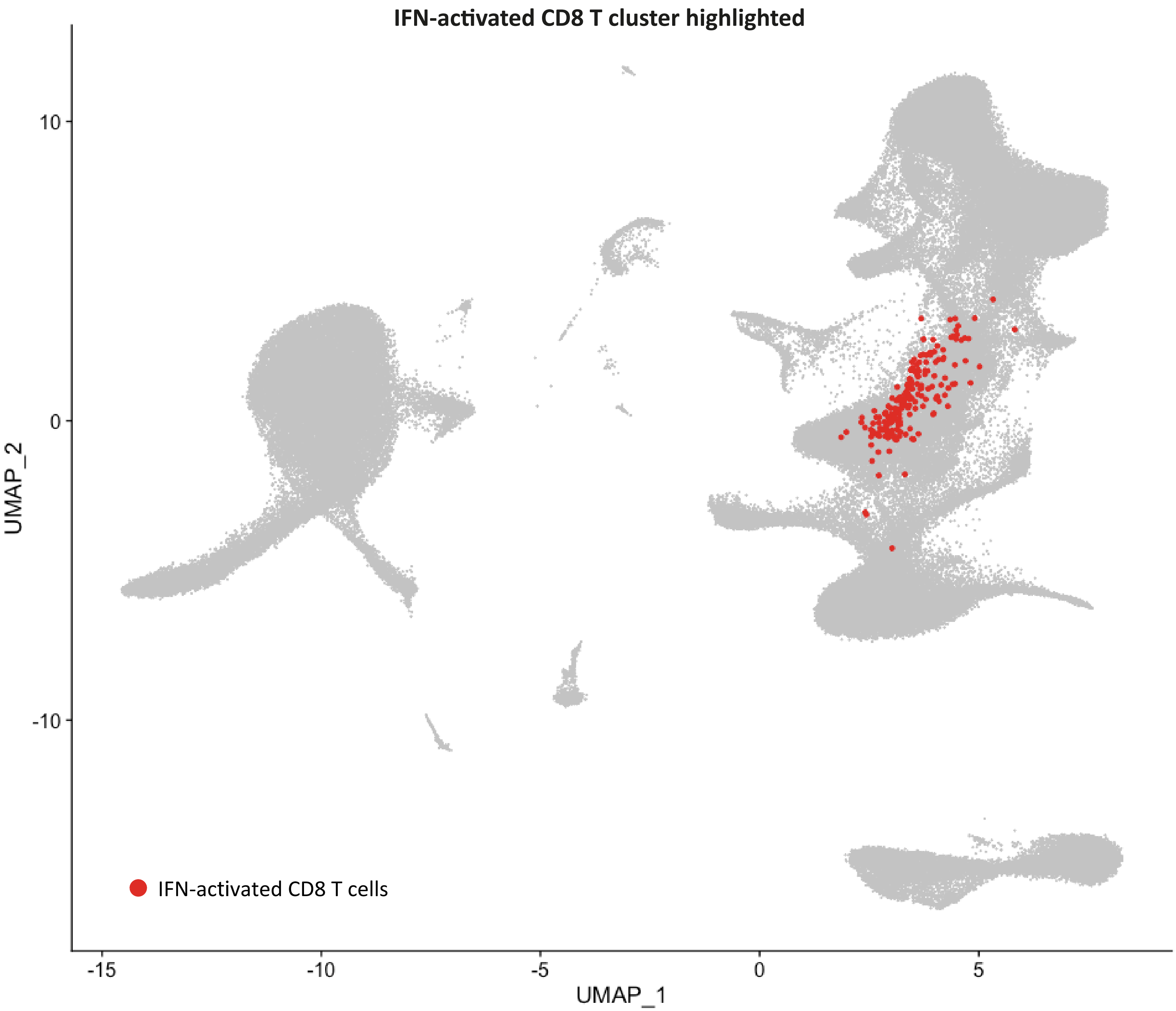

Supplementary Figure 4 IFN-activated CD8 T cell cluster highlighted
UMAP with the cells belonging to the IFN-activated CD8 T cell cluster highlighted in red. We found a higher percentage of cells belonging to the IFN-activated CD8 T cluster (out of all CD8 T cells) in COVID-19 vs Controls (p=0.00578, as calculated by Chi-Square test).

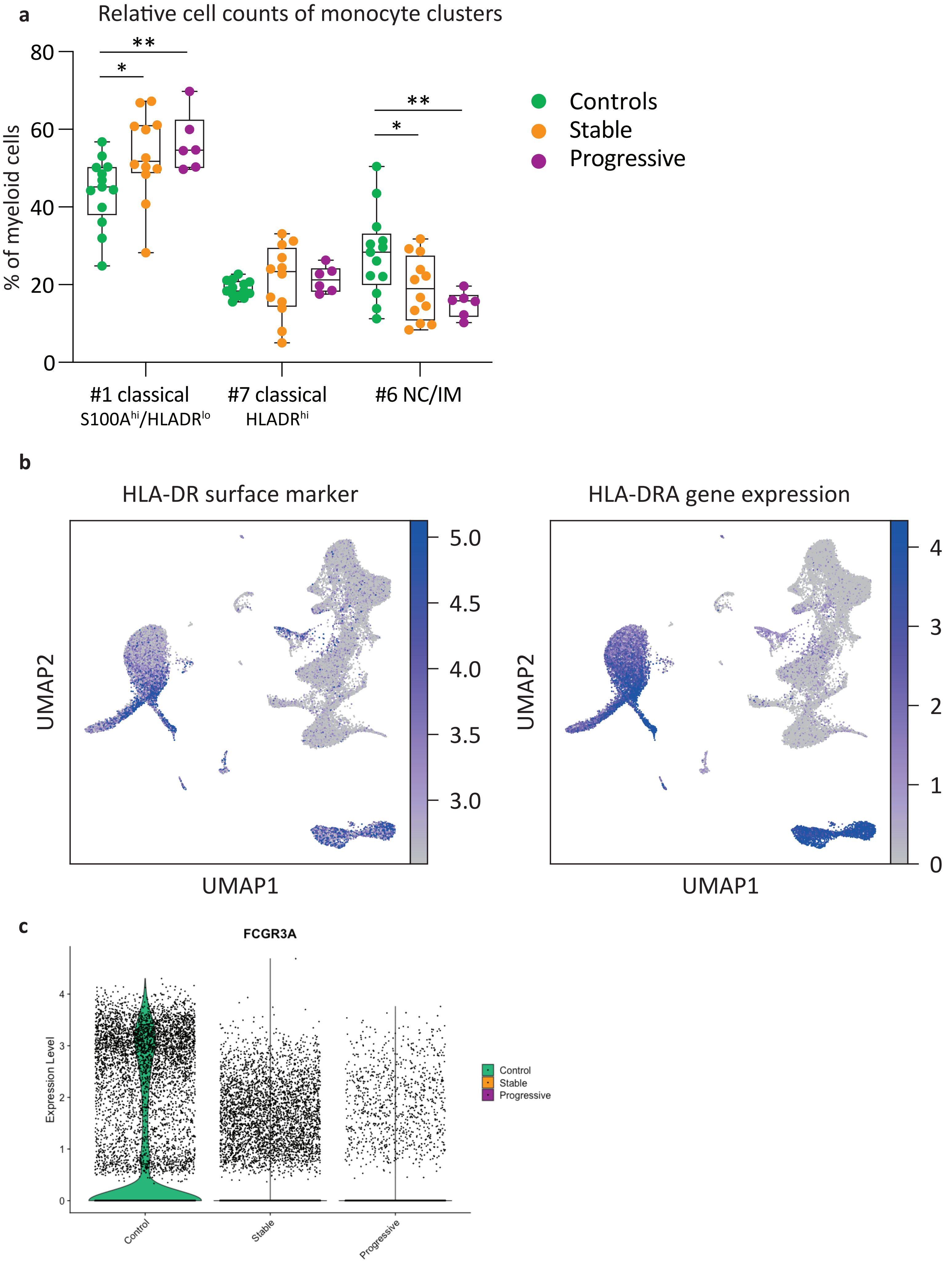
a
Relative cell counts of monocyte clusters
% of myeloid cells
0
20
40
60
80
**
*
#1 classical
S100A^hi/HLADR^lo
#7 classical
HLADR^hi
#6 NC/IM
Controls
Stable
Progressive
b
HLA-DR surface marker
HLA-DRA gene expression
UMAP2
UMAP1
5.0
4.5
4.0
3.5
3.0
4
3
2
1
0
c
FCGR3A
Expression Level
Control
Stable
Progressive

Supplementary Figure 5 Relative cell counts of the monocyte clusters, *HLA-DR* and *FCGR3A* expression

a Relative cell counts of monocyte clusters, showing an increase of S100A^{hi}/HLA-DR^{lo} monocytes in COVID-19, and a decrease in non-classical (NC)/indeterminate (IM) monocytes. The results are depicted in boxplots, in which the upper and lower bounds represent the 75% and 25% percentiles, respectively, the bars inside the boxplots denoting the medians, the whiskers denoting values up to 1.5 interquartile range (IQR) above the 75% percentile or below the 25% percentile. *, p-value < 0.05; **, p-value < 0.01. Two-way ANOVA with Tukey's multiple comparison test was performed. N = 13 for Controls, 12 for Stable, and 6 for Progressive.

b UMAPs depicting the expression of HLA-DR surface marker, as measured by CITE-seq, and HLADRA gene expression, showing two populations of classical monocytes: HLA-DR^{lo} (top) and HLA-DR^{hi}. Refer also to figure 1e.

c Lower *FCGR3A* expression in monocytes of Stable and Progressive COVID-19 patients compared to Controls.

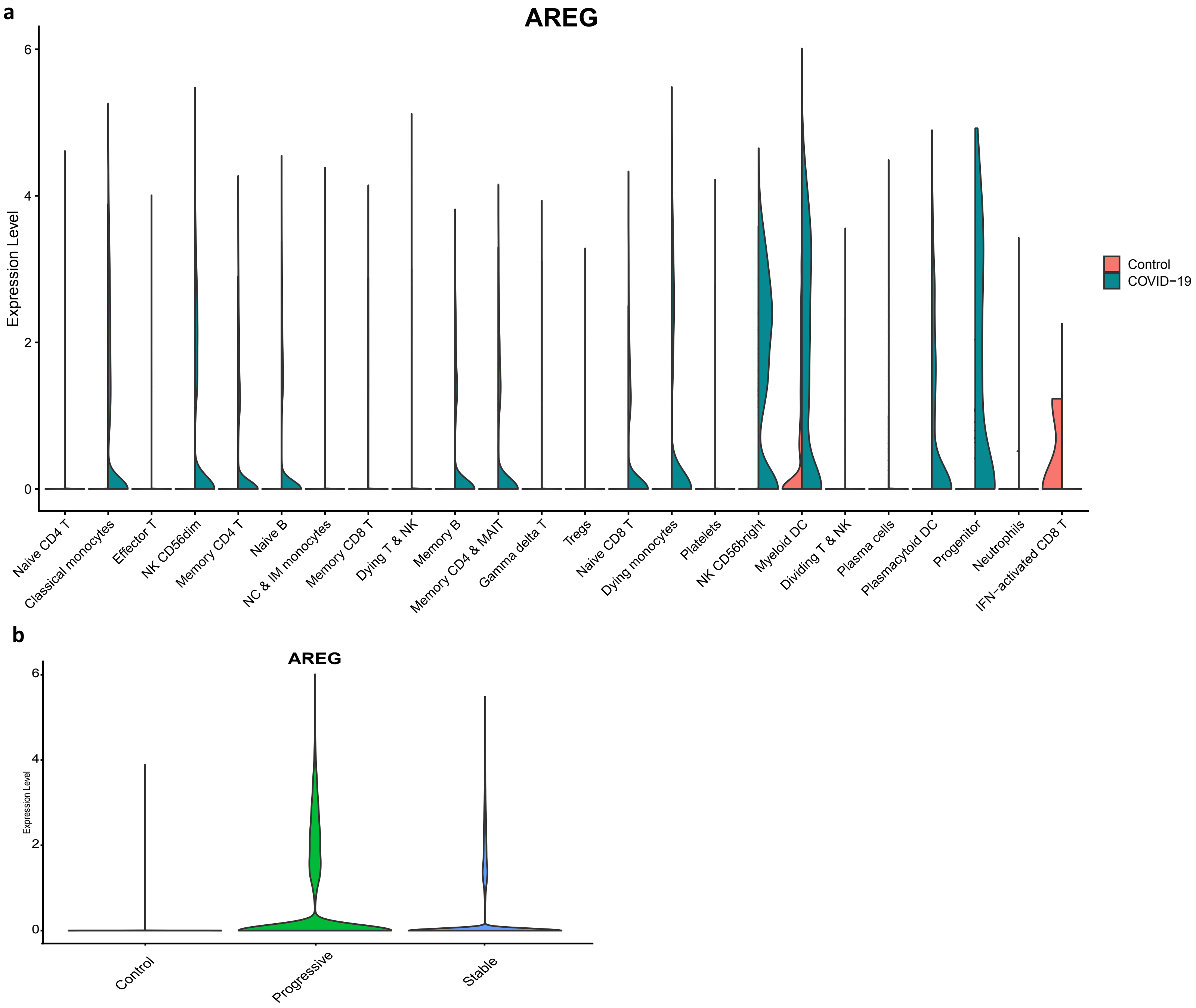

Supplementary Figure 6 ***AREG* expression**

a A comparison of the expression of *AREG i*n different cell types between control samples and all COVID-19 (progressive and stable) samples.

b Shown is the average expression of *AREG* between the different groups of COVID-19 patients and Controls.

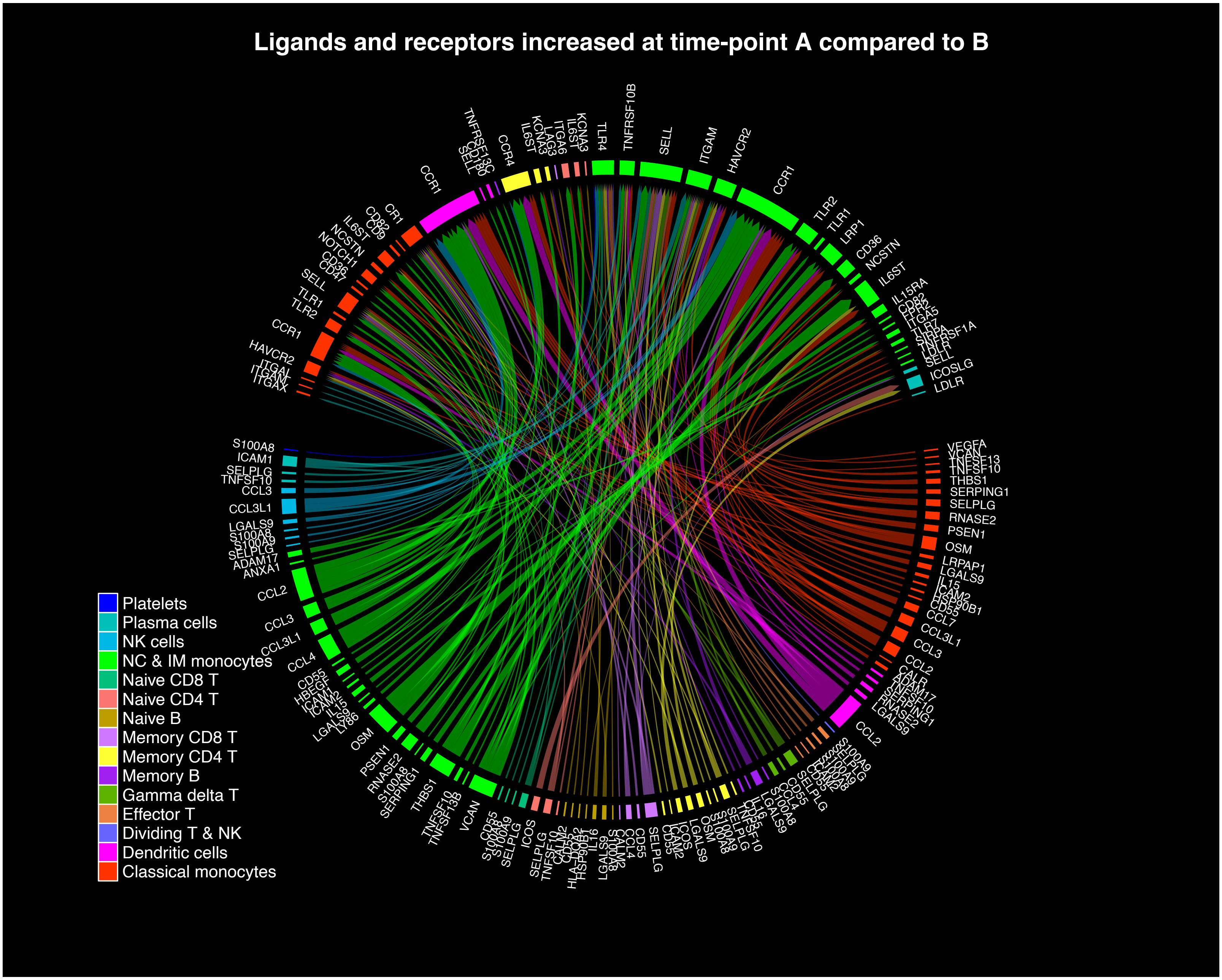

Supplementary Figure 7 Ligands and receptors increased in PBMCs at time-point A compared to B

Shown are differential connectivity maps ("connectomes") of ligands (bottom half) and receptors (upper half) that are increased at time-point A compared to time-point B. For each cell type, log fold changes of ligands and receptors were calculated, comparing progressive to stable COVID-19 patients; we only plotted edges with >10% of cells expressing the ligand and receptor, and with an adjusted p value<0.05 for the comparison, calculated by Wilcoxon rank-sum test with Bonferroni correction for multiple comparisons; edge size is proportional to the degree of change between progressive and stable patients.

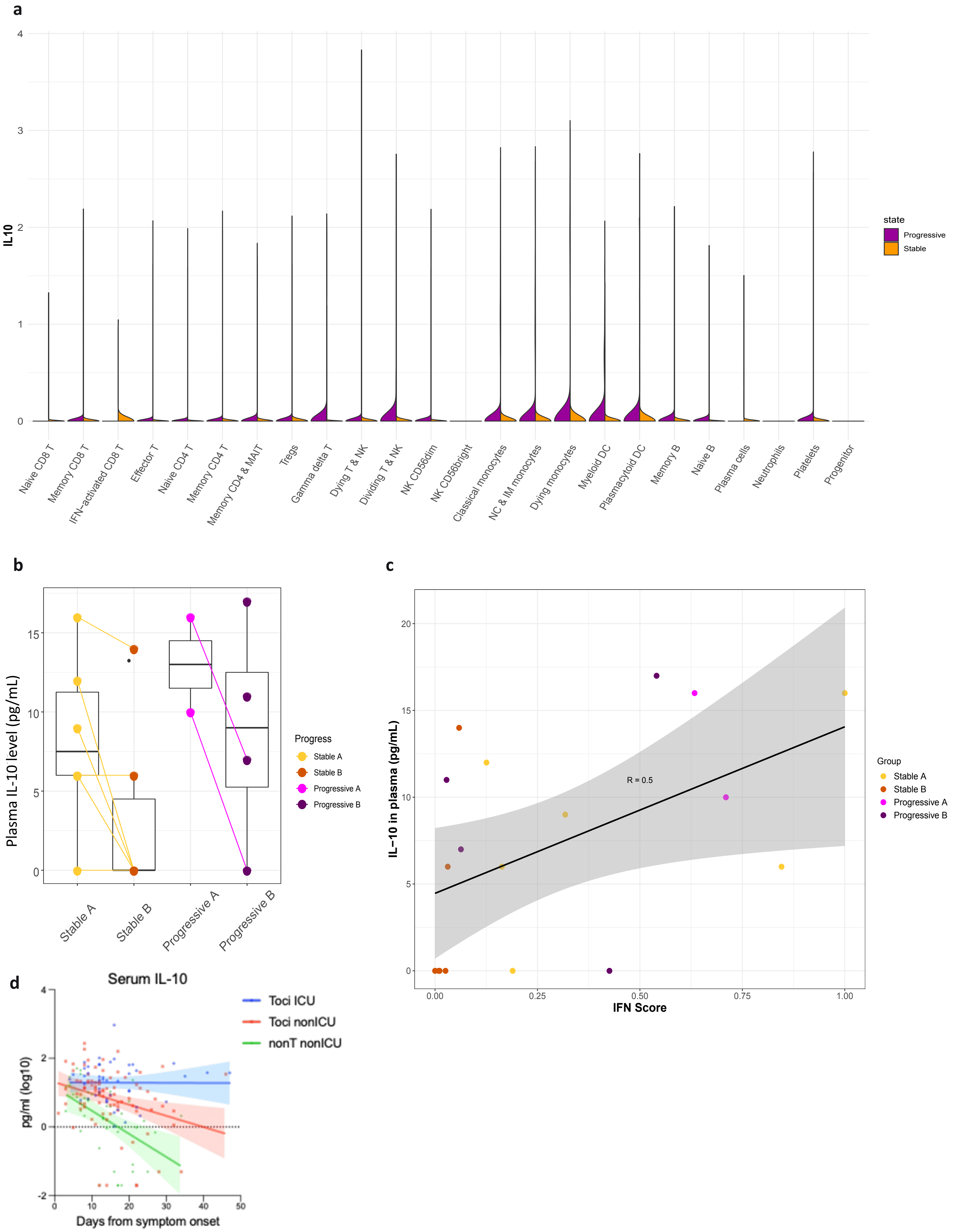
a
IL10
state
Progressive
Stable
Naive CD8 T
Memory CD8 T
IFN-activated CD8 T
Effector T
Naive CD4 T
Memory CD4 T
Memory CD4 & MAIT
Tregs
Gamma delta T
Dying T & NK
Dividing T & NK
NK CD56dim
NK CD56bright
Classical monocytes
NC & IM monocytes
Dying monocytes
Myeloid DC
Plasmacytoid DC
Memory B
Naive B
Plasma cells
Neutrophils
Platelets
Progenitor
b
Plasma IL-10 level (pg/mL)
Progress
Stable A
Stable B
Progressive A
Progressive B
c
IL−10 in plasma (pg/mL)
IFN Score
R = 0.5
Group
Stable A
Stable B
Progressive A
Progressive B
d
Serum IL-10
pg/ml (log10)
Days from symptom onset
Toci ICU
Toci nonICU
nonT nonICU

Supplementary Figure 8 ***IL10*** **expression in progressive vs stable patients, and correlation with IFN-I score**

a *IL10* expression in Progressive vs Stable COVID-19 patients, according to cell type, as measured by scRNA-seq.

b Plasma IL-10 protein levels in our patients according to disease severity and time. The results are depicted in boxplots, in which the upper and lower bounds represent the 75% and 25% percentiles, respectively, the bars inside the boxplots denoting the medians, the whiskers denoting values up to 1.5 interquartile range (IQR) above the 75% percentile or below the 25% percentile. N = 6 for Stable A, 6 for Stable B, 2 for Progressive A, and 4 for Progressive B.

c Scatter plot showing correlation between plasma IL-10 protein level and IFN-I score for each patient at each time-point (R=0.5). Error bands denote 95% confidence interval.

d Serum IL-10 concentration in a larger cohort of COVID-19 patients was plotted over time. Regression lines are shown as blue (patients admitted to ICU and treated with tocilizumab; Toci ICU), red (patients not admitted to ICU and treated with tocilizumab; Toci nonICU), and green (patients not admitted to ICU and not treated with tocilizumab; nonT nonICU). Error bands denote 95% confidence interval.

a

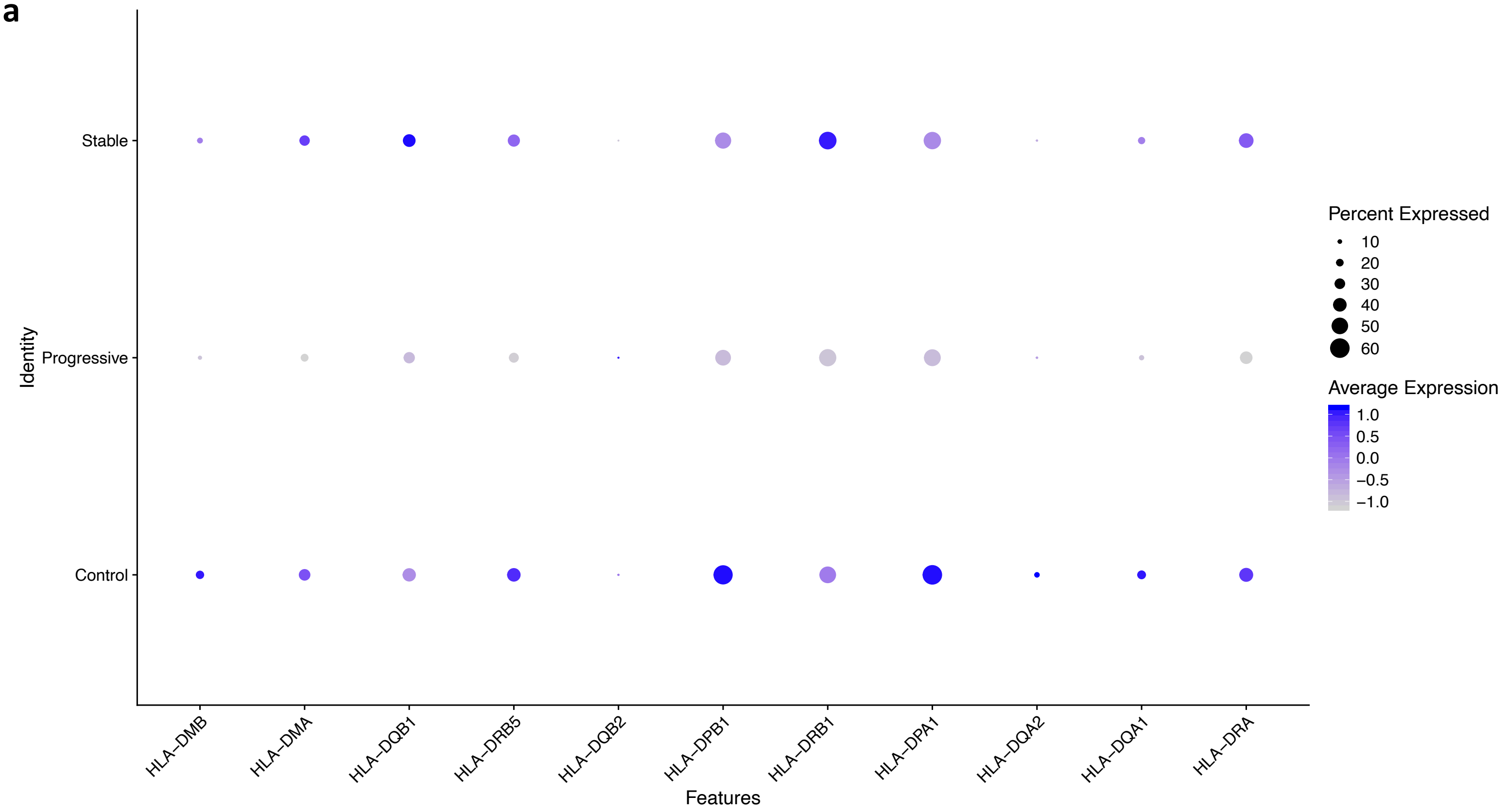

b

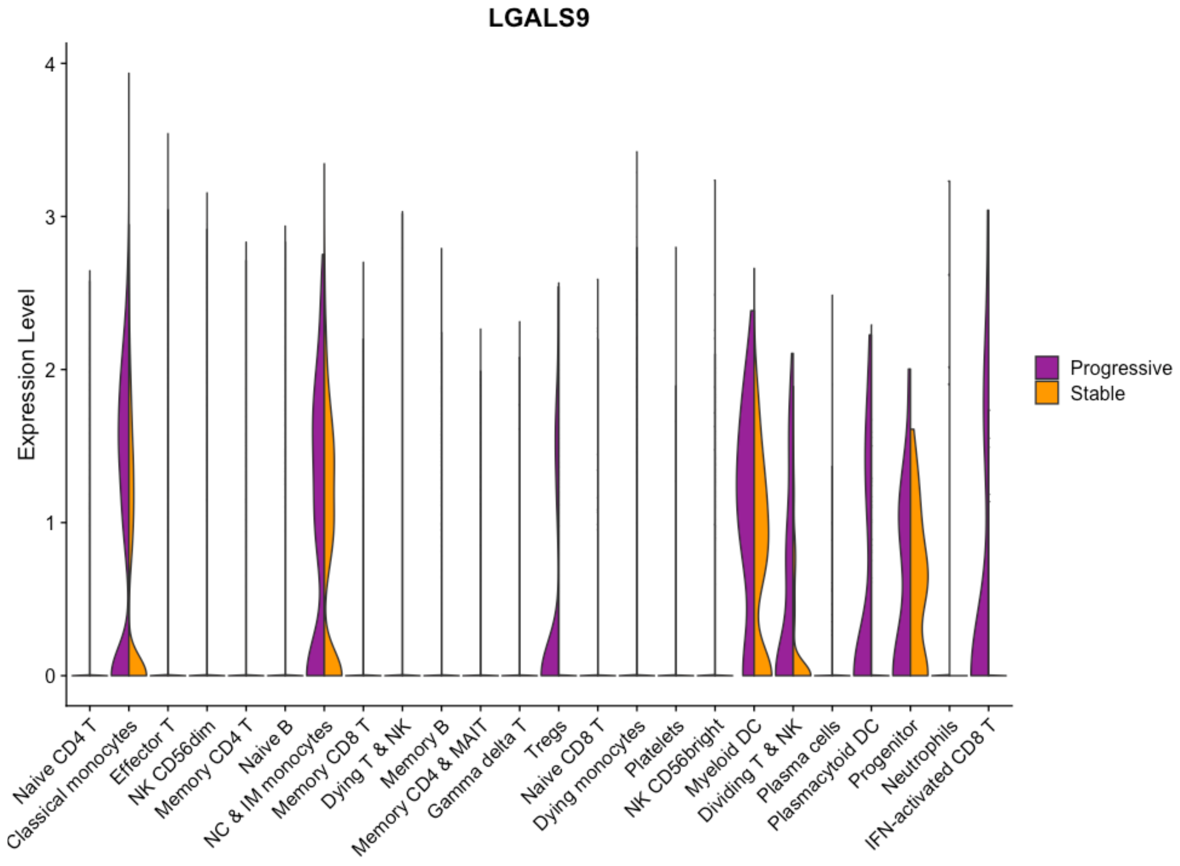

Supplementary Figure 9 Expression of MHC-II molecules in stable and progressive COVID-19 patients vs controls; Expression of *LGALS9*

a A dot plot depicting the expression of different MHC-II molecules in different patient subgroups. The size of each dot indicates the percentage of cells that express the indicated molecule, and the dot's color indicates the average expression level of each molecule.
b A violin plot depicting *LGALS9* expression levels in various cell types in progressive and stable COVID-19 patients.

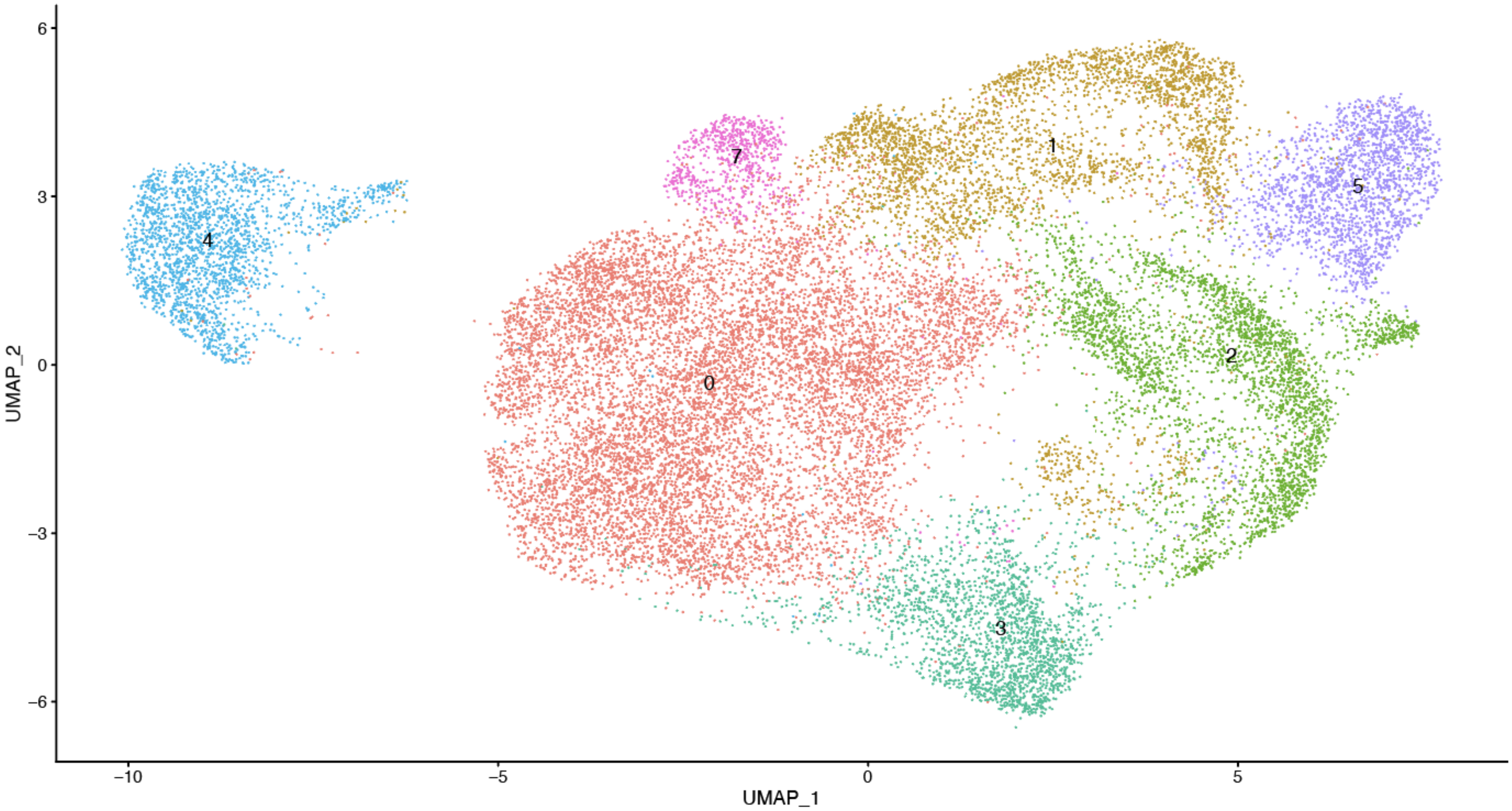

Supplementary Figure 10 UMAP of monocytes (sub-clustered)
Shown is a UMAP of monocytes following sub-clustering, with cluster numbers from largest (0) to smallest (7).

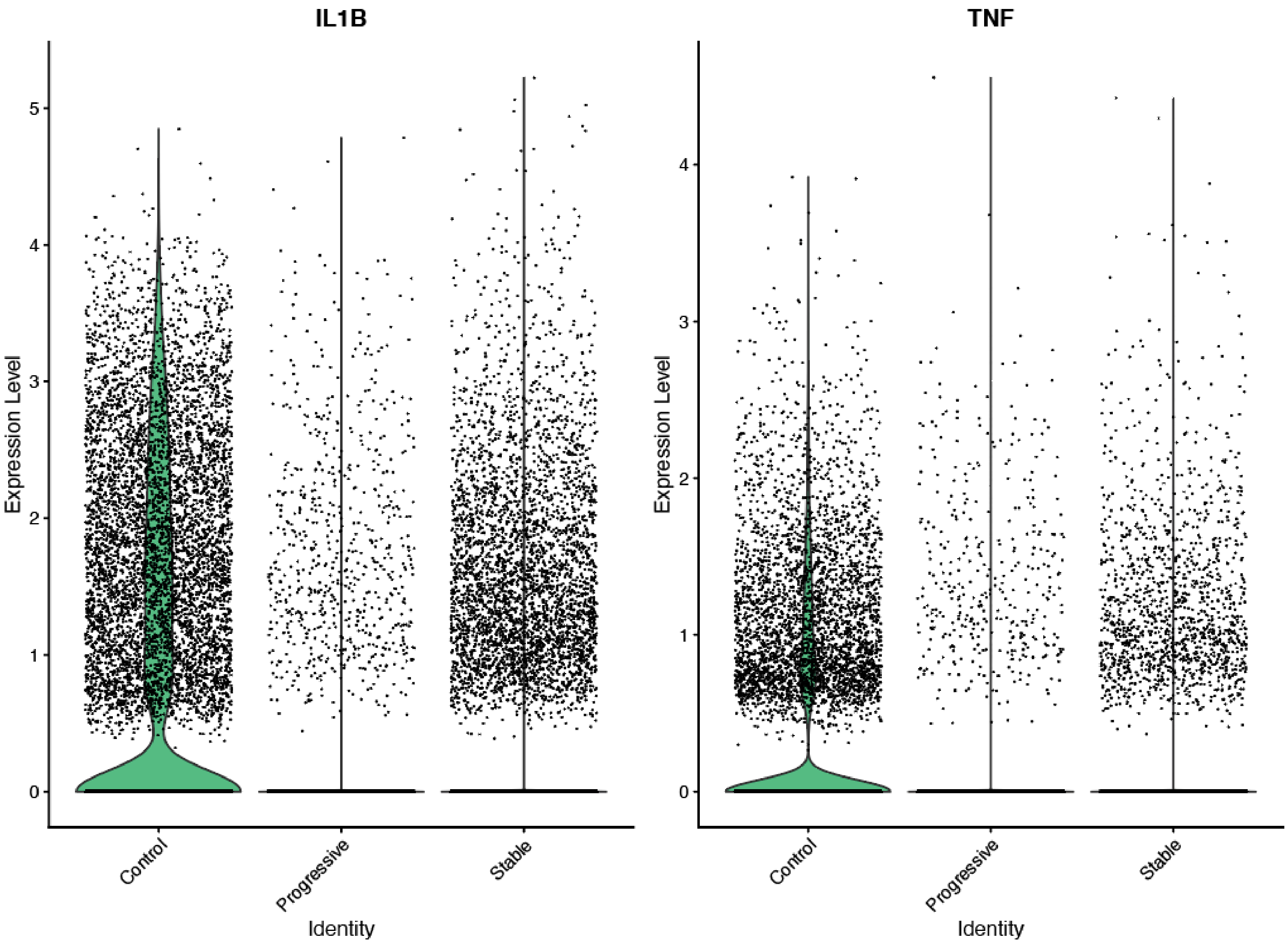

Supplementary Figure 11 ***IL1B*** **and** ***TNF*** **expression in monocytes**
Violin plots depicting the expression levels of *IL1B* and *TNF* in monocytes for different patient subgroups.

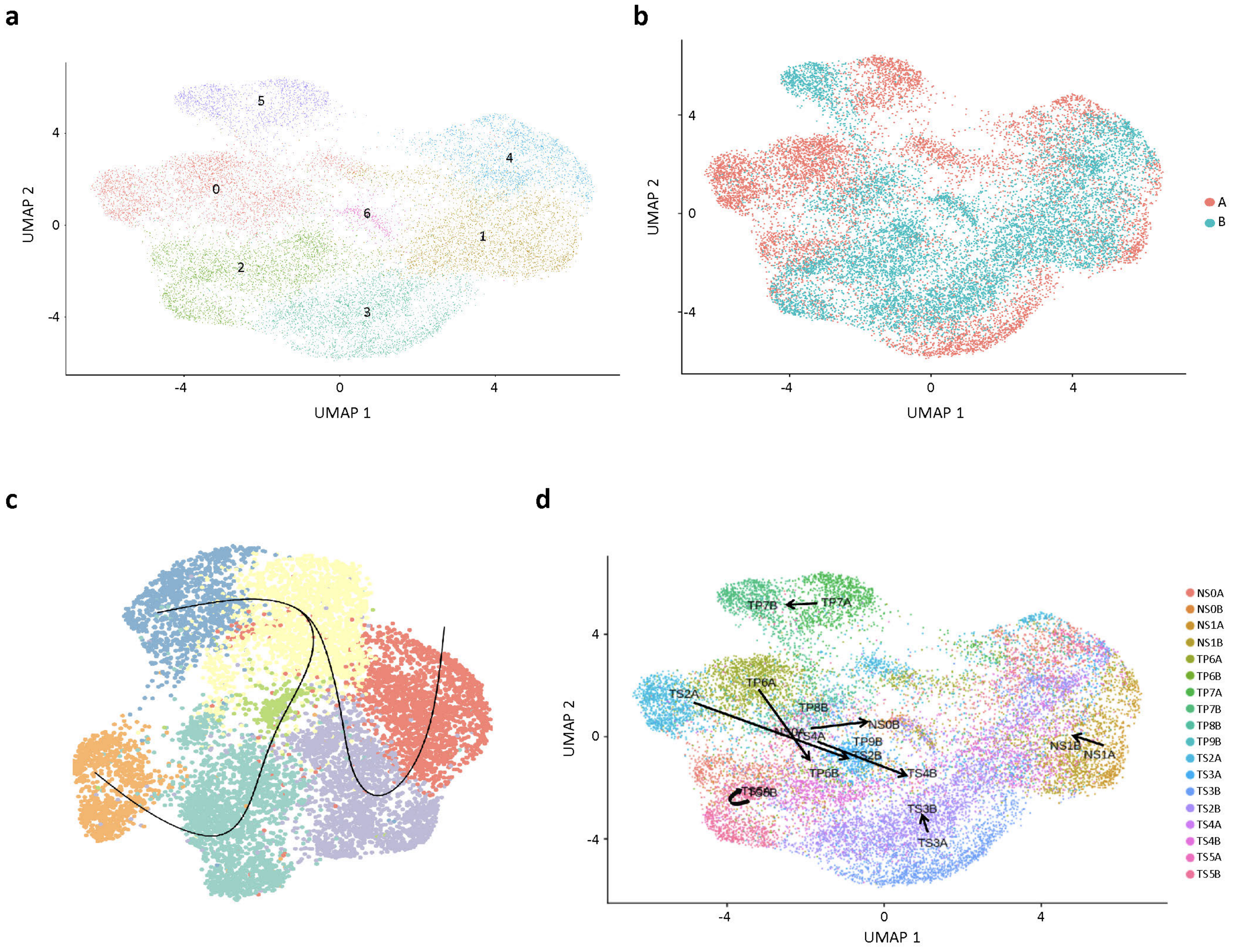

Supplementary Figure 12 UMAP of CD8⁺ T cells (sub-clustered) and shifts in gene expression from time A to B
This Figure is supplementary to Fig. 4e.

a Subclustering of all CD8⁺ T cells, with cluster numbers from largest (0) to smallest (6).

b UMAP showing differences between time-point A and B on the UMAP space.

c Pseudotime trajectories diverge towards the two poles of effector T cells at time-point A: one enriched with progressive samples and the other with stable ones.

d Arrows represent the shift on a UMAP space from time point A to B for each subject, with a general trend of moving from the periphery to the center, except for subject TP7 which maintains a high IFN score and high viral loads at time-point B.

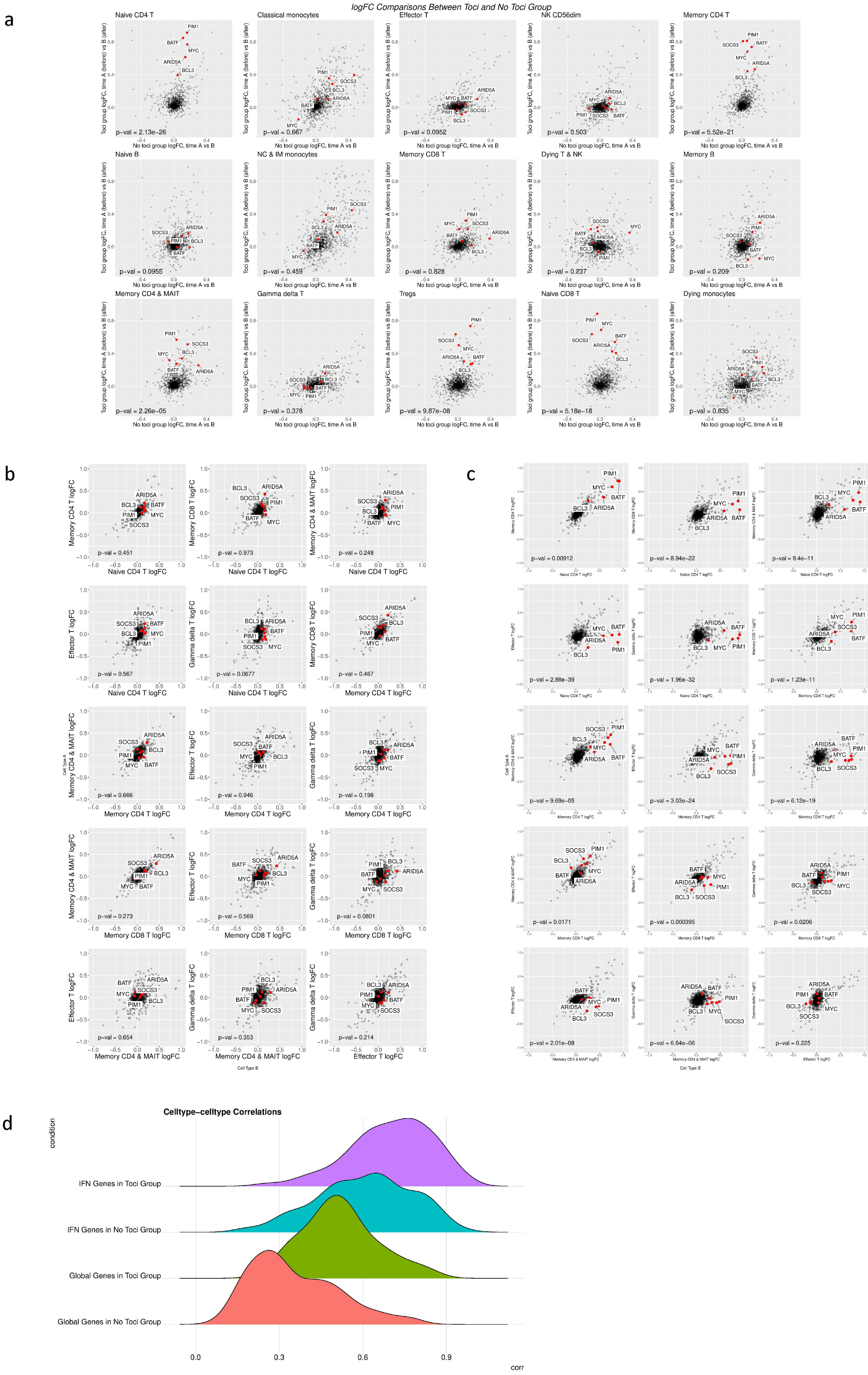
a
logFC Comparisons Between Toci and No Toci Group
Naive CD4 T
Classical monocytes
Effector T
NK CD56dim
Memory CD4 T
Naive B
NC & IM monocytes
Memory CD8 T
Dying T & NK
Memory B
Memory CD4 & MAIT
Gamma delta T
Tregs
Naive CD8 T
Dying monocytes
Toci group logFC, time A (before) vs B (after)
No toci group logFC, time A vs B
p−val = 2.13e−26
p−val = 0.667
p−val = 0.0952
p−val = 0.503
p−val = 5.52e−21
p−val = 0.0955
p−val = 0.459
p−val = 0.828
p−val = 0.237
p−val = 0.209
p−val = 2.26e−05
p−val = 0.378
p−val = 9.87e−08
p−val = 5.18e−18
p−val = 0.835
PIM1
BATF
MYC
ARID5A
BCL3
SOCS3
b
c
Cell Type A
Cell Type B
d
Celltype−celltype Correlations
condition
IFN Genes in Toci Group
IFN Genes in No Toci Group
Global Genes in Toci Group
Global Genes in No Toci Group
0.0
0.3
0.6
0.9
corr

Supplementary Figure 13 Analysis of the effects of tocilizumab

a Scatterplots of gene expression log fold-change (logFC), comparing tocilizumab-treated (Toci) group and non-tocilizumab-treated (non-Toci) group across the major cell types. Six IL-6 pathway genes are highlighted in red. X- and Y-axis are on the same scale. The vertical distributed patterns of highlighted genes indicate the treatment effects after removing confounding effects due to progressive time. To evaluate the differential logFC statistically, we tested and showed the p values of the interaction effects between time points and the IL-6 pathway genes by the linear model.

b - c Scatterplots for cell type-cell type correlations within Toci group (b) and non-Toci group (c) with the same scale of X- and Y-axis. The selected plots are the logFC comparisons between two time points from major T cell type pairs. The highlighted genes are the six IL-6 pathway genes. The cell type pairs have higher correlations if the highlighted genes as well as the global genes are distributed around the diagonal. To evaluate the differential logFC statistically, we tested and showed the p values of the interaction effects between the cell type and the IL-6 pathway genes by the linear model

d Ridgeplots of cell type-cell type correlation distribution across different groups. The cell type pair correlations are measured either by ISGs or the global 2000 highly variable genes within the Toci group and non-Toci group. The larger mean of the correlation distribution underlies a more coherent response to disease across different cell types.

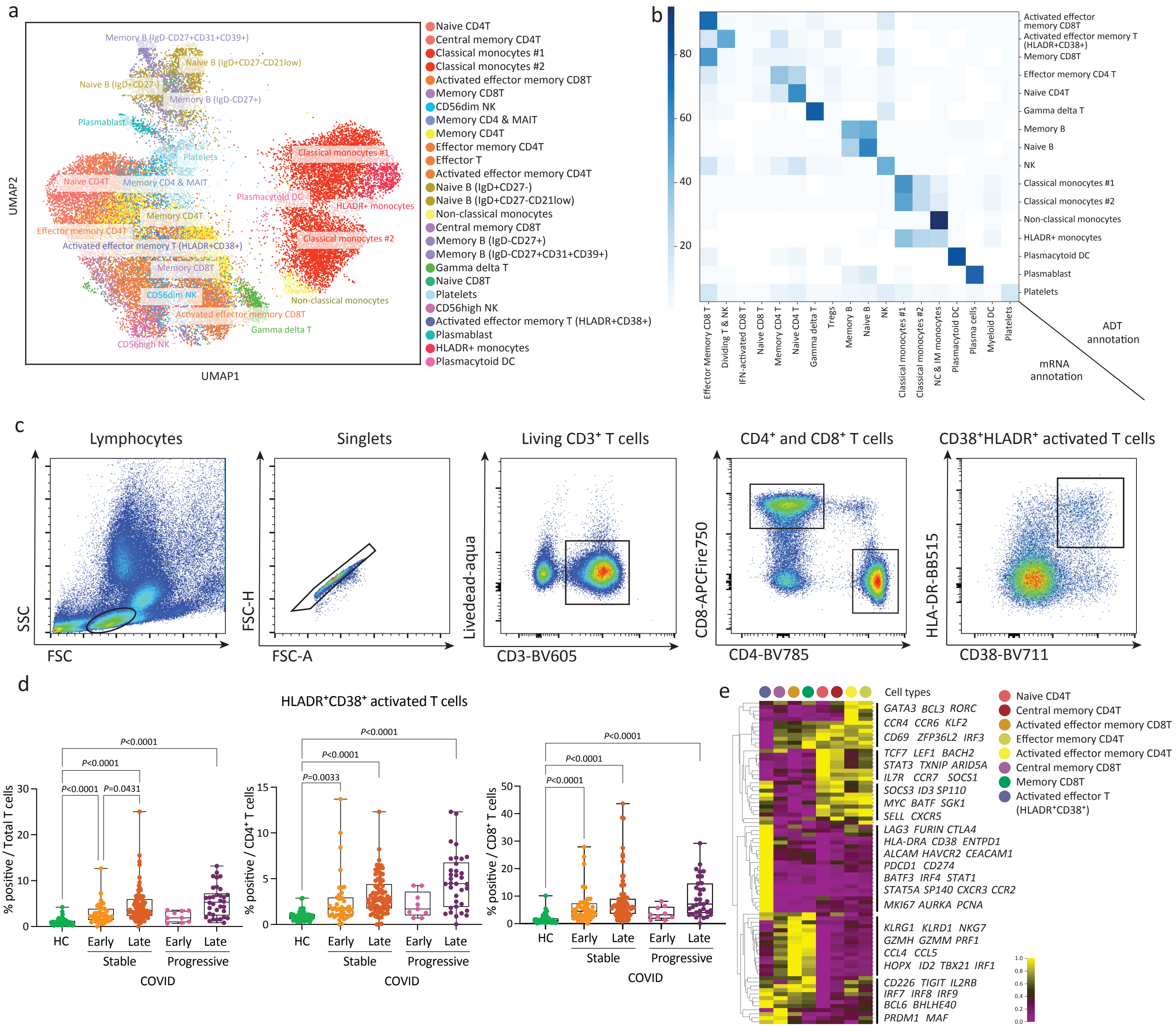

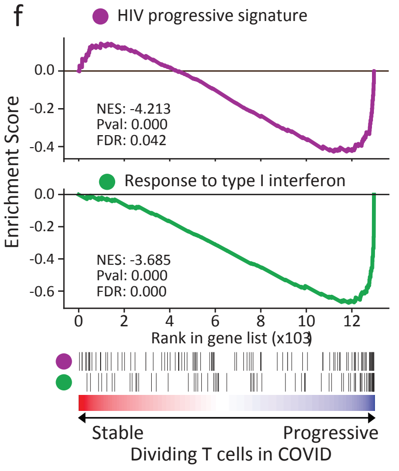

Supplementary Figure 14. CITE-seq and flow cytometry analysis

a UMAP embedding of the ADT data from 43,349 cells across COVID-19 patients, excluding a cluster of non-specific staining (8,032 cells, see results section). **b** Heatmap showing the overlap between ADT and RNA based annotations. **c** Gating strategy for the identification of CD38$^+$ HLADR$^+$ CD4$^+$ or CD8$^+$ T cells by Flow cytometry. **d** Flow cytometry data for the fraction of HLADR+CD38+ T cells over total T cells (left), CD4+ T cells (middle), and CD8+ T cells (right). Stable and progressive COVID-19 patients were divided into early and late time points (early; days from symptom onset <10, late; days from symptom onset >10). **e** Heatmap showing the expression of curated genes across the different T cell clusters. **f** GSEA of a signature of HIV specific CD8+ T cells in progressive chronic HIV patients (GSE24081) and type-1 IFN response (GO:0034340). Progressive HIV CD8+ T cell signature (purple), and type 1 IFN response signature (green) in the ranked list of genes differentially expressed by dividing T cells from stable versus progressive COVID-19 patients. Significance was assessed by Kruskal-Wallis rank-sum test with Dunn's post hoc test for multiple comparisons. Boxplots represent the median and bottom and upper quartiles; whiskers correspond to minimum and maximum values.

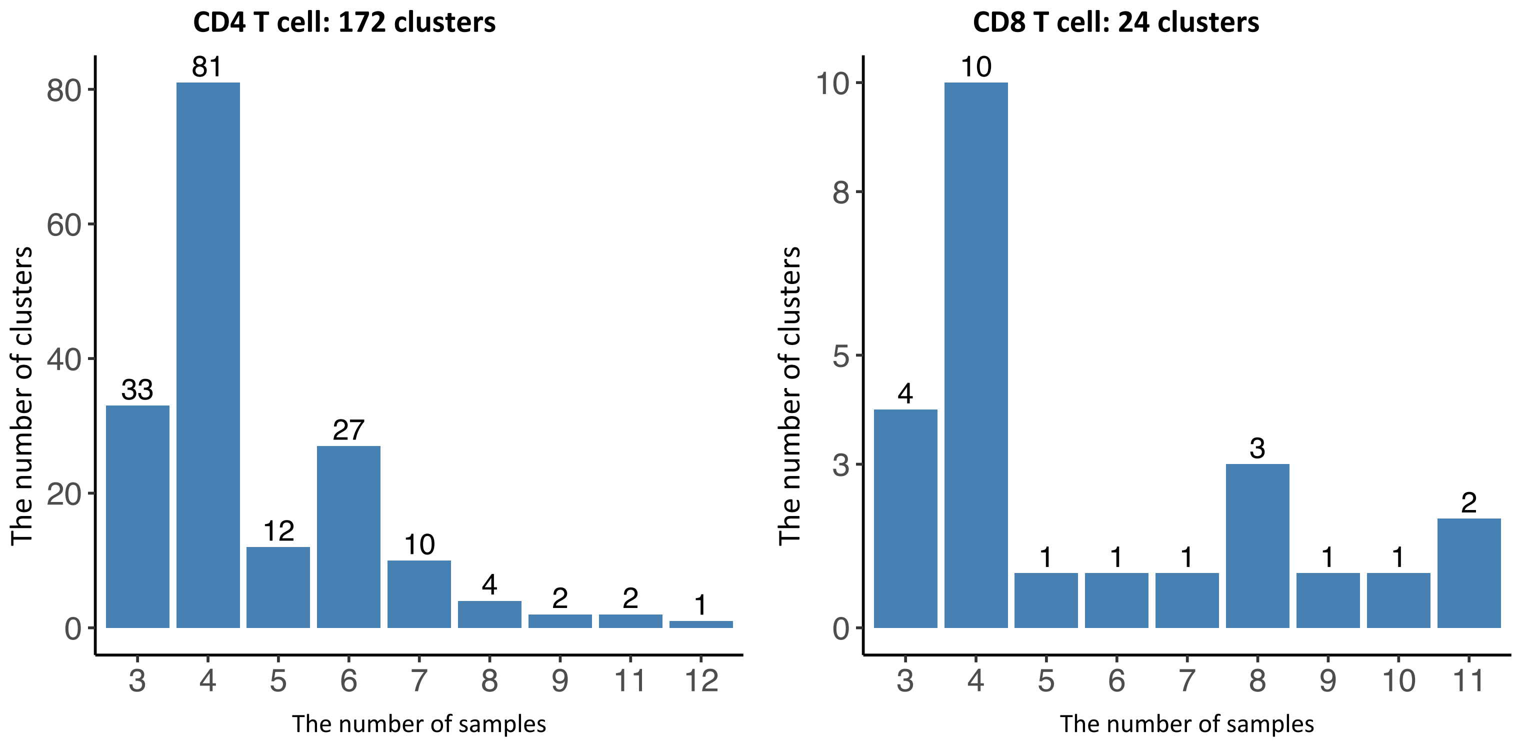

Supplementary Figure 15. TCR clone clusters overlapped between multiple subjects
TCR clone clusters overlapped between multiple subjects. Barplots show the number of TCR clone clusters with clones from more than two samples identified by GLIPH2 in $CD4^+$ T cells (left panel) and $CD8^+$ T cells (right panel) based on the top 172 $CD4^+$ and 24 $CD8^+$ T cell SARS-CoV-2 specific clone clusters.

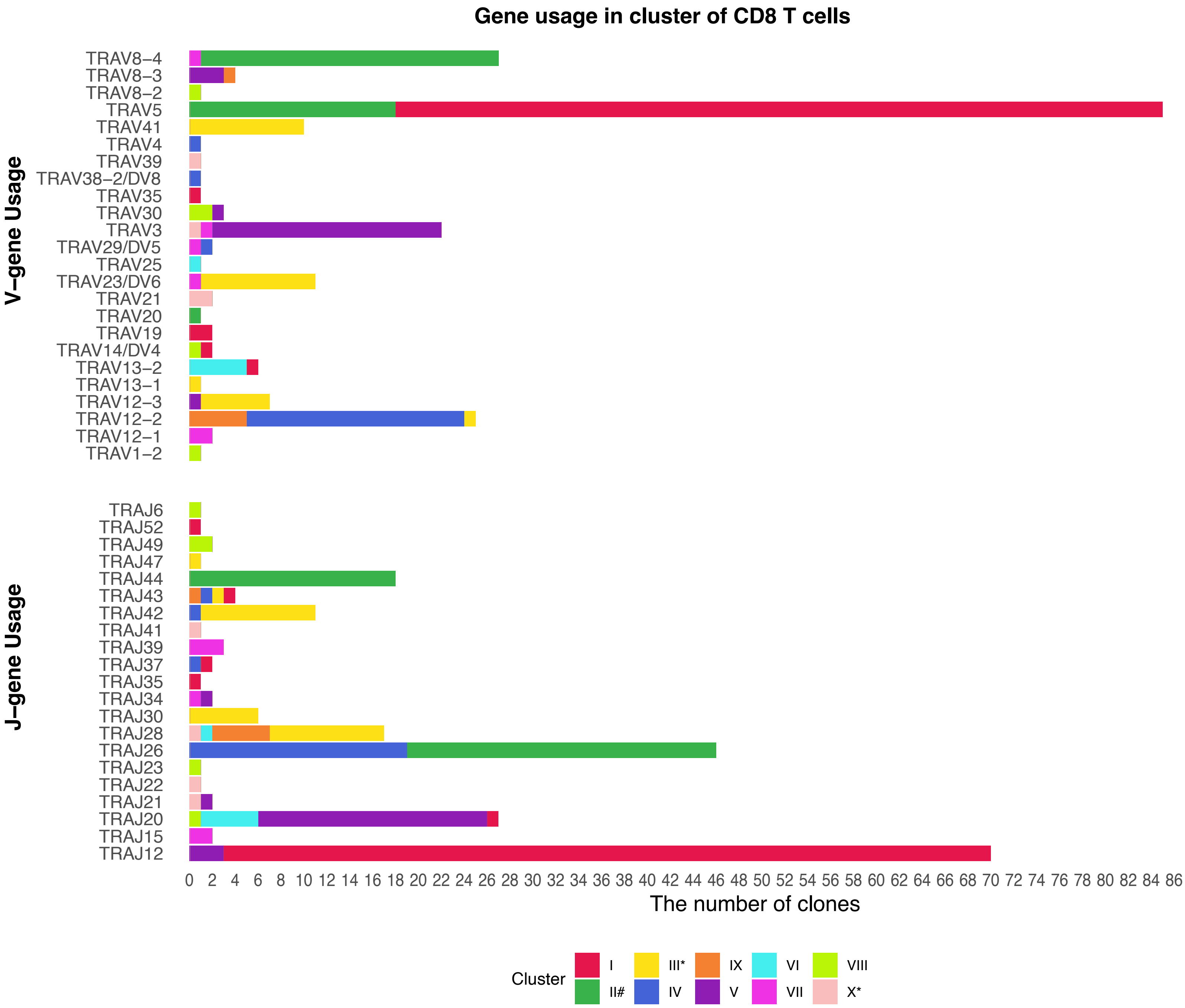

Supplementary Figure 16. TCRα gene usage in CD8⁺ T cells that belong to COVID19-specific CDR3 clusters

The TRAV and TRAJ gene usage distribution based on the top 10 SARS-CoV-2 specific expanded clone clusters in CD8⁺ T cells. The numbers of clones using each V-gene (top panel) and J-gene (bottom panel) across all clone clusters are shown in bar plots. Different colors represent different CDR3 clusters.

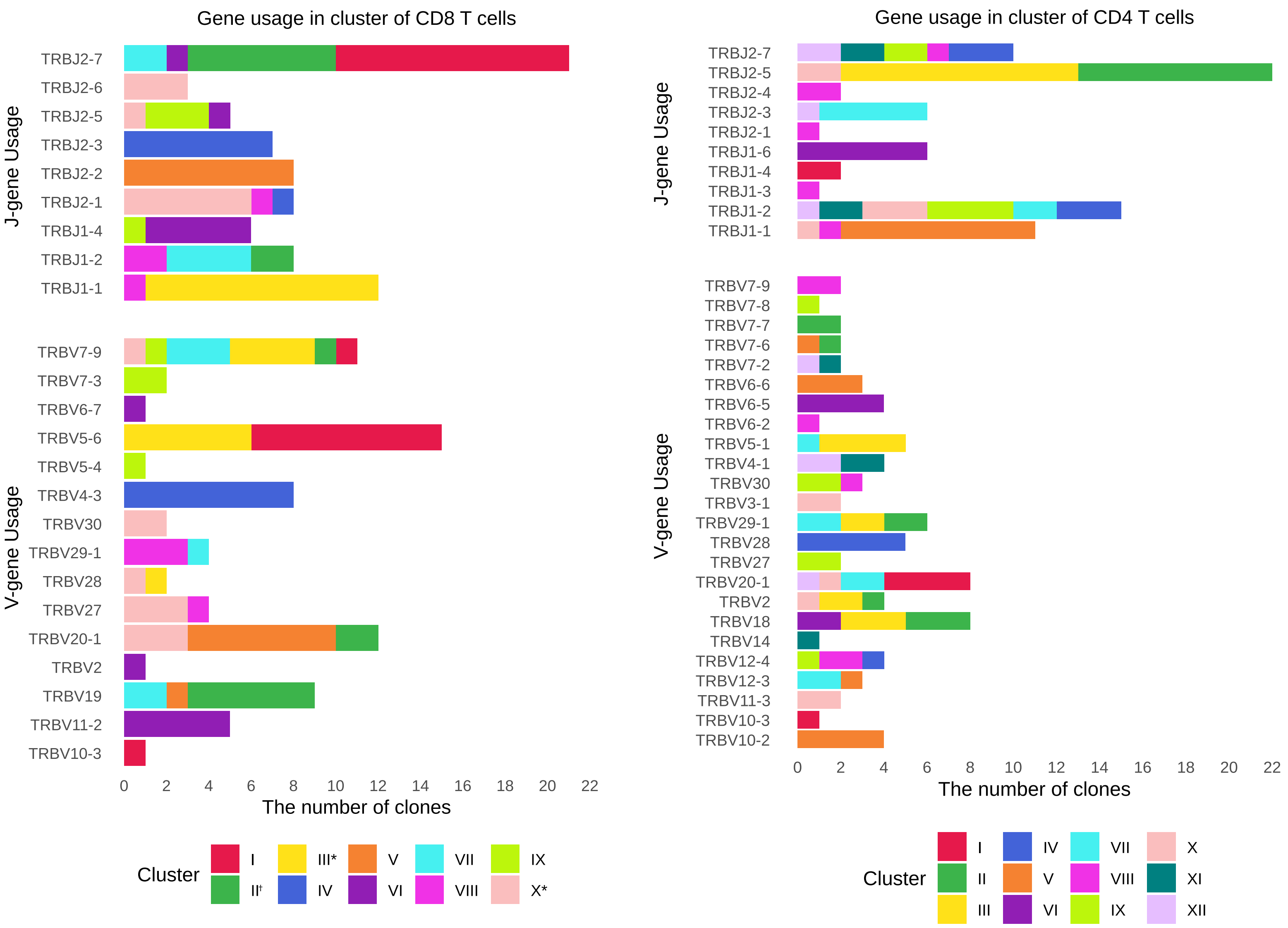

Supplementary Figure 17. Gene usage of the 10 and 12 specificity groups in CD8⁺ and CD4⁺ T cells.
Stacked barplots show the TRBV (bottom) and TRBJ (top) gene usage distribution based on the top 10 and top 12 SARS-CoV-2 specific clone clusters in CD8⁺ T cells (left panel) and CD4⁺ T cells (right panel), respectively.

index	sample	TcRb	TcRa	HLA.A		HLA.B		HLA.C		HLA.DPA1		HLA.DPB1		HLA.DQA1		HLA.DQB1		HLA.DRB1	
Group: I (Index: 993; global-GG%QE)	NS1A	SAEGGVQETQY	AVRGTSGSRLT	03:71	26:01	38:01	38:01	12:03	12:03	01:03	01:03	76:01	76:01	01:02	01:25	06:04	06:04	04:02	04:02
	TP6B	AISGGTQETQY	AANYNTDKLI	30:01	30:01	15:361	15:361	17:01	17:01	02:01	02:01	01:01	50:01	02:01	02:01	02:02	02:02	07:01	07:01
	NS0A	SESGGGQETQY	AVNDGQKLL	01:01	01:01	08:01	15:16	07:441	16:42	01:03	02:01	01:01	45:01	05:01	05:01	05:03!	05:03!	14:54	14:54
	TS3A	SAKGGGQEKLF	AVMAAGNKLT	29:02!	29:02!	50:31!	50:31!	16:85	16:85	01:03	01:03	49:01!	49:01!	01:01	01:25	05:02!	05:02!	01:01	01:01
	TS3B	SAKGGGQEKLF	AVMAAGNKLT	29:116!	29:116!	50:31!	50:31!	04:166	04:166	01:03	01:03	49:01!	72:01	01:01	01:25	05:02!	06:02	01:01	01:01
Group: II (Index: 2820; global-RD%QE)	TS3B	ASSRDRDETQY	ALARTGNQFY	29:116	29:116	50:31	50:31	04:166	04:166	01:03	01:03	49:01	72:01	01:01	01:25	05:02	06:02	01:01	01:01
	TS2B	SVERDRQETQY	AAYLIYNQGGKLI	02:02	02:02	07:02	07:02	04:01	04:01	01:03	01:03	584:01	584:01	02:01	02:01	06:09	06:09	07:01	07:01
	TS2A	SVERDRQETQY	AAYLIYNQGGKLI	02:02	02:02	07:02	07:02	04:01	04:01	01:03	01:03	584:01	584:01	02:01	02:01	06:09	06:09	07:01	07:01
	NS1B	ASNRDRQETQY	VVSRNSGGSNYKLT	03:71	26:01	38:01	38:01	12:03	12:03	01:03	01:03	76:01	76:01	01:02	01:25	05:02	05:02	16:01	16:01
	TS3A	ASSRDREETQY	AMREGASGTYKYI	29:02	29:02	50:31	50:31	16:85	16:85	01:03	01:03	49:01	49:01	01:01	01:25	05:02	05:02	01:01	01:01
	TS3B	ASSRDREETQY	AMREGASGTYKYI	29:116	29:116	50:31	50:31	04:166	04:166	01:03	01:03	49:01	72:01	01:01	01:25	05:02	06:02	01:01	01:01
	NS1B	ASSRDRQETQY	NA	03:71	26:01	38:01	38:01	12:03	12:03	01:03	01:03	76:01	76:01	01:02	01:25	05:02	05:02	16:01	16:01
	TP6A	ASSRDRQETQY	NA	30:01	30:01	42:01	42:01	14:111	17:01	02:02	02:02	01:01	59:01	02:01	02:01	04:02	04:02	07:01	07:01
	TS4B	ASSRDRQETQY	NA	25:01	25:01	18:01	18:01	12:03	12:03	01:03	02:01	76:01	76:01	01:02	01:02	06:02	06:02	12:01	12:01

Supplementary Figure 18. HLA alleles present within the 2 specificity groups in CD4+ T cells.
For each specific group, samples with contributing clones are shown together with their corresponding TcRb and TcRa sequences, followed by their HLA alleles. The predicted common HLA class II alleles for each specificity group are highlighted using exclamation marks. HLA alleles with the same first two fields are highlighted in yellow and those with the same first field are highlighted in green.

index	sample	TcRb	TcRa	HLA.A		HLA.B		HLA.C		HLA.DPA1		HLA.DPB1		HLA.DQA1		HLA.DQB1		HLA.DRB1	
Group: I (Index: 207; global-LS%Y)	NS1A	ATRLSTYEQY	ALSGSVDNDMR	03:71	26:01	38:01	38:01	12:03	12:03	01:03	01:03	76:01	76:01	01:02	01:25	06:04	06:04	04:02	04:02
	NS0A	ASSLSIYEQY	ALSAIVSLGFGNVLH	01:01	01:01	08:01	15:16	07:441	16:42	01:03	02:01	01:01	45:01	05:01	05:01	05:03!	05:03!	14:54	14:54
	TS3B	ASSLSTYEQY	AETMDSSYKLI	29:116!	29:116!	50:31!	50:31!	04:166	04:166	01:03	01:03	49:01!	72:01	01:01	01:25	05:02!	06:02	01:01	01:01
	TS3A	ASSLSTYEQY	AETMDSSYKLI	29:02!	29:02!	50:31!	50:31!	16:85	16:85	01:03	01:03	49:01!	49:01!	01:01	01:25	05:02!	05:02!	01:01	01:01
	TS3A	ASSLSTYEQY	NA	29:02!	29:02!	50:31!	50:31!	16:85	16:85	01:03	01:03	49:01!	49:01!	01:01	01:25	05:02!	05:02!	01:01	01:01
	TS3B	ASSLSTYEQY	NA	29:116!	29:116!	50:31!	50:31!	04:166	04:166	01:03	01:03	49:01!	72:01	01:01	01:25	05:02!	06:02	01:01	01:01
	TS4B	ASSLSTYEQY	AETMDSSYKLI	25:01	25:01	18:01	18:01	12:03	12:03	01:03	02:01	76:01	76:01	01:02	01:02	06:02	06:02	12:01	12:01
	TS3A	ASSLSTYEQY	AETMDSSYKLI	29:02!	29:02!	50:31!	50:31!	16:85	16:85	01:03	01:03	49:01!	49:01!	01:01	01:25	05:02!	05:02!	01:01	01:01
	TS3A	ASSLSTYEQY	AMREGAGSGNTGKLI	29:02!	29:02!	50:31!	50:31!	16:85	16:85	01:03	01:03	49:01!	49:01!	01:01	01:25	05:02!	05:02!	01:01	01:01
	TS3A	ASSLSTYEQY	AESQNDYKLS	29:02!	29:02!	50:31!	50:31!	16:85	16:85	01:03	01:03	49:01!	49:01!	01:01	01:25	05:02!	05:02!	01:01	01:01
	TS3A	ASSLSTYEQY	GGTSYGKLT	29:02!	29:02!	50:31!	50:31!	16:85	16:85	01:03	01:03	49:01!	49:01!	01:01	01:25	05:02!	05:02!	01:01	01:01
Group: II (index: 173; global-D%GSY)	TP7A	ASSDRGSYEQY	AVRSDYGQNFV	01:251	68:152!	14:01!	14:01!	04:01	04:01	01:03	02:01	13:01!	13:01!	01:01!	01:01!	03:01	03:01	11:04	11:04
	TP7A	ASSDRGSYEQY	AEKPGTASKLT	01:251	68:152!	14:01!	14:01!	04:01	04:01	01:03	02:01	13:01!	13:01!	01:01!	01:01!	03:01	03:01	11:04	11:04
	TP7B	ASSDRGSYEQY	AVRSDYGQNFV	01:251	68:152!	14:06!	14:06!	04:01	04:01	01:03	02:01	13:01!	13:01!	01:01!	01:01!	03:01	03:01	01:02	01:02
	TP7B	ASSDRGSYEQY	AEKPGTASKLT	01:251	68:152!	14:06!	14:06!	04:01	04:01	01:03	02:01	13:01!	13:01!	01:01!	01:01!	03:01	03:01	01:02	01:02
	TP7B	ASSDRGSYEQY	NA	01:251	68:152!	14:06!	14:06!	04:01	04:01	01:03	02:01	13:01!	13:01!	01:01!	01:01!	03:01	03:01	01:02	01:02
	TP7A	ASSDRGSYEQY	NA	01:251	68:152!	14:01!	14:01!	04:01	04:01	01:03	02:01	13:01!	13:01!	01:01!	01:01!	03:01	03:01	11:04	11:04
	TP7B	SARDHGSYGYT	NA	01:251	68:152!	14:06!	14:06!	04:01	04:01	01:03	02:01	13:01!	13:01!	01:01!	01:01!	03:01	03:01	01:02	01:02
	TS4B	SARDHGSYGYT	NA	25:01	25:01	18:01	18:01	12:03	12:03	01:03	02:01	76:01	76:01	01:02!	01:02!	06:02	06:02	12:01	12:01
	NS1B	ASEDSGSYEQY	AVWDNYGQNFV	03:71	26:01	38:01	38:01	12:03	12:03	01:03	01:03	76:01	76:01	01:02!	01:25!	05:02	05:02	16:01	16:01
Group: III (Index: 73; global-F%GT)	TP7A	ASSFPGTEAF	AMSARSMNRDDKII	01:251	68:152!	14:01!	14:01!	04:01	04:01	01:03	02:01	13:01!	13:01!	01:01!	01:01!	03:01	03:01	11:04	11:04
	TP7B	ASSFPGTEAF	AMSARSMNRDDKII	01:251	68:152!	14:06!	14:06!	04:01	04:01	01:03	02:01	13:01!	13:01!	01:01!	01:01!	03:01	03:01	01:02	01:02
	TP7A	ASSFPGTEAF	NA	01:251	68:152!	14:01!	14:01!	04:01	04:01	01:03	02:01	13:01!	13:01!	01:01!	01:01!	03:01	03:01	11:04	11:04
	TP7B	ASSFPGTEAF	NA	01:251	68:152!	14:06!	14:06!	04:01	04:01	01:03	02:01	13:01!	13:01!	01:01!	01:01!	03:01	03:01	01:02	01:02
	TS3A	ASSFSGTEAF	AAGPFAYSGAGSYQLT	29:02!	29:02!	50:31!	50:31!	16:85	16:85	01:03	01:03	49:01!	49:01!	01:01!	01:25!	05:02	05:02	01:01	01:01
	TS3A	ASSFSGTEAF	ARGHGSQGNLI	29:02!	29:02!	50:31!	50:31!	16:85	16:85	01:03	01:03	49:01!	49:01!	01:01!	01:25!	05:02	05:02	01:01	01:01
	TS3B	ASSFSGTEAF	ARGHGSQGNLI	29:116!	29:116!	50:31!	50:31!	04:166	04:166	01:03	01:03	49:01!	72:01	01:01!	01:25!	05:02	06:02	01:01	01:01
	TS3B	ASSFSGTEAF	AAGPFAYSGAGSYQLT	29:116!	29:116!	50:31!	50:31!	04:166	04:166	01:03	01:03	49:01!	72:01	01:01!	01:25!	05:02	06:02	01:01	01:01
	TS3A	ASSFSGTEAF	NA	29:02!	29:02!	50:31!	50:31!	16:85	16:85	01:03	01:03	49:01!	49:01!	01:01!	01:25!	05:02	05:02	01:01	01:01
	NS1B	ASSFVGTEAF	AVRGNNNDMR	03:71	26:01	38:01	38:01	12:03	12:03	01:03	01:03	76:01	76:01	01:02!	01:25!	05:02	05:02	16:01	16:01
	TS4B	ASSFGGTEAF	AARAGYGNKLV	25:01	25:01	18:01	18:01	12:03	12:03	01:03	02:01	76:01	76:01	01:02!	01:02!	06:02	06:02	12:01	12:01
Group: V (Index: 193; global-T%TG)	TS3B	AIETNTGELF	GRNDKLI	29:116	29:116	50:31	50:31	04:166	04:166	01:03	01:03	49:01	72:01	01:01	01:25	05:02	06:02	01:01!	01:01!
	TS5B	SAPTNTGELF	AVRDMGQRYKLS	02:58	02:58	39:31!	39:31!	01:06!	01:06!	01:03	02:01	677:01	678:01	02:01	02:01	03:03	03:03	01:03!	01:03!
	TS5A	SAPTNTGELF	AVRDMGQRYKLS	02:01	02:01	39:31!	39:31!	01:06!	07:429	01:03	02:01	02:01	677:01	02:01	02:01	03:19	03:19	01:03!	01:03!
	TS5A	SAPTNTGELF	AVGARDSSYKLI	02:01	02:01	39:31!	39:31!	01:06!	07:429	01:03	02:01	02:01	677:01	02:01	02:01	03:19	03:19	01:03!	01:03!
	TS5A	SAPTNTGELF	NA	02:01	02:01	39:31!	39:31!	01:06!	07:429	01:03	02:01	02:01	677:01	02:01	02:01	03:19	03:19	01:03!	01:03!
	TS5B	SAPTNTGELF	AVGARDSSYKLI	02:58	02:58	39:31!	39:31!	01:06!	01:06!	01:03	02:01	677:01	678:01	02:01	02:01	03:03	03:03	01:03!	01:03!
	TS5B	SAPTNTGELF	NA	02:58	02:58	39:31!	39:31!	01:06!	01:06!	01:03	02:01	677:01	678:01	02:01	02:01	03:03	03:03	01:03!	01:03!
	NS1B	SAQTRTGELF	AMSASSNFNKFY	03:71	26:01	38:01	38:01	12:03	12:03	01:03	01:03	76:01	76:01	01:02	01:25	05:02	05:02	16:01	16:01

Supplementary Figure 19. HLA alleles present within the 4 specificity groups in CD8$^+$ T cells.
For each specific group, samples with contributing clones are shown together with their corresponding TcRb and TcRa sequences, followed by their HLA alleles. The predicted common HLA class II alleles for each specificity group are highlighted using exclamation marks. HLA alleles with the same first two fields are highlighted in yellow and those with the same first field are highlighted in green.

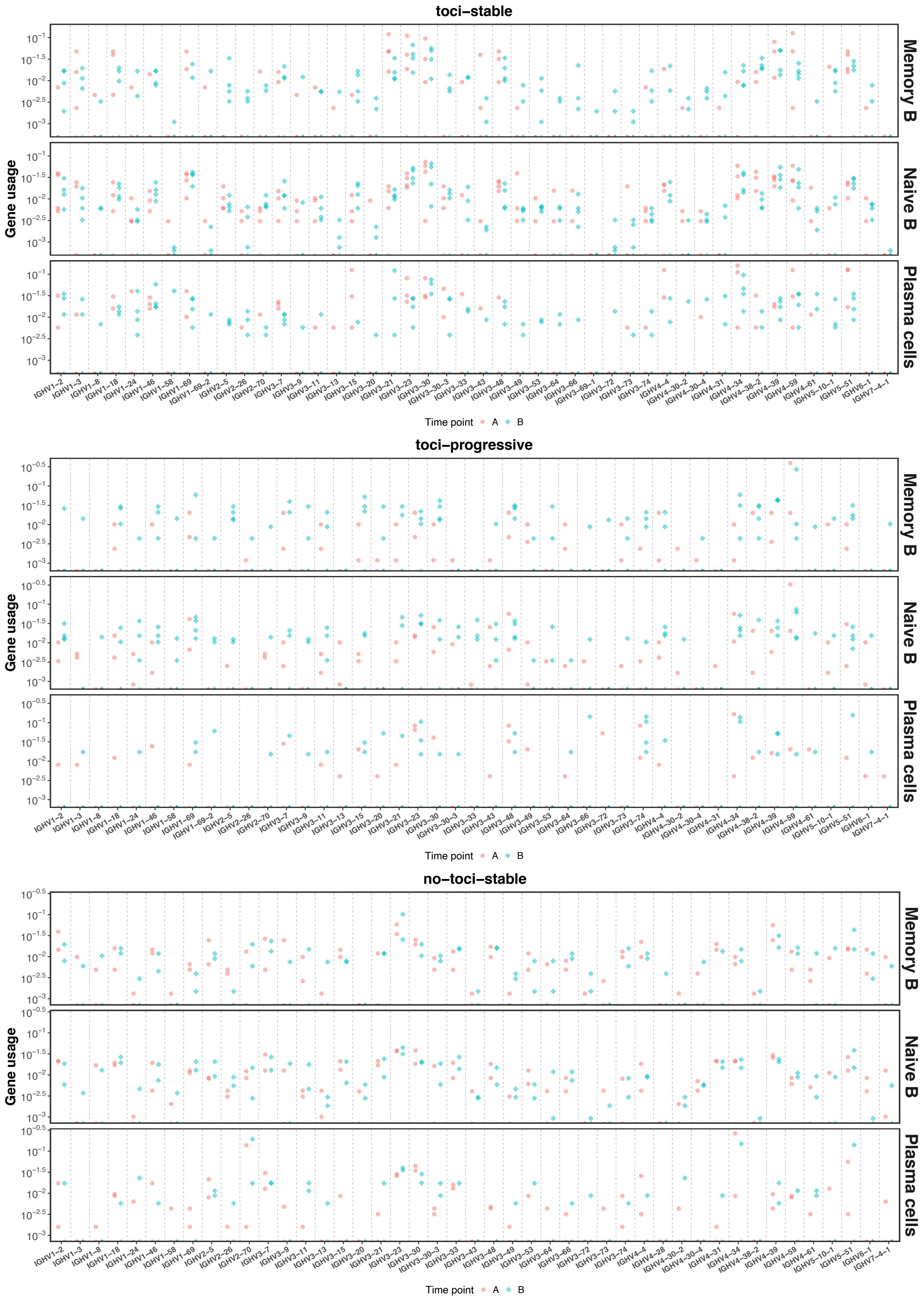

Supplementary Figure 20. Gene usage dynamics.
Gene usage across three cell-types, at each time for different patient (point) groups: stable patients under tocilizumab treatment (top panel); progressive patients under tocilizumab treatment (middle panel); and stable patients under no treatment (bottom panel).

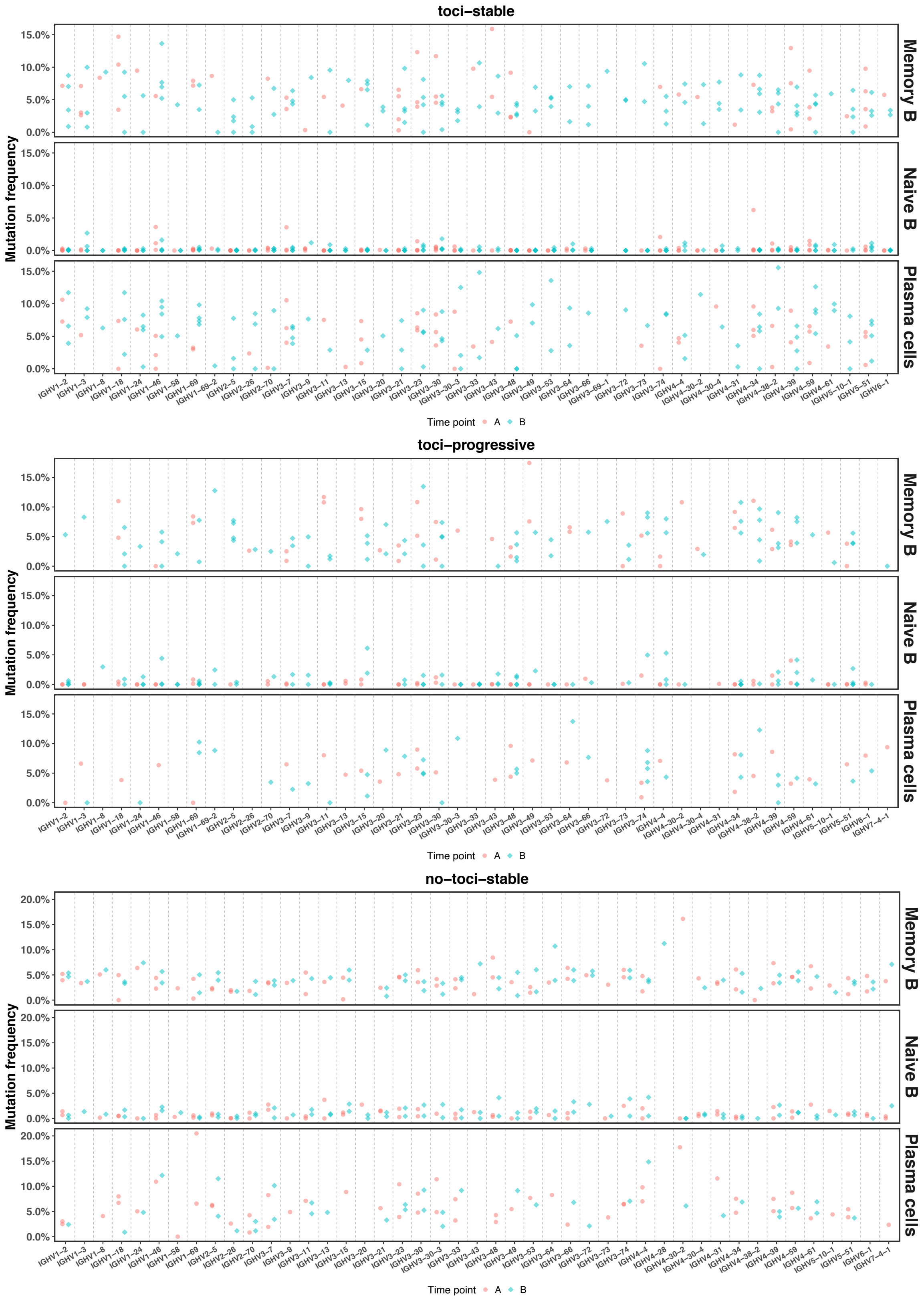

Supplementary Figure 21. Mutation frequency dynamics.

Mutation across three cell-types, at each time point (A and B), for different patient (point) groups: stable patients under tocilizumab treatment (top panel); progressive patients under tocilizumab treatment (middle panel); and stable patients under no treatment (bottom panel).

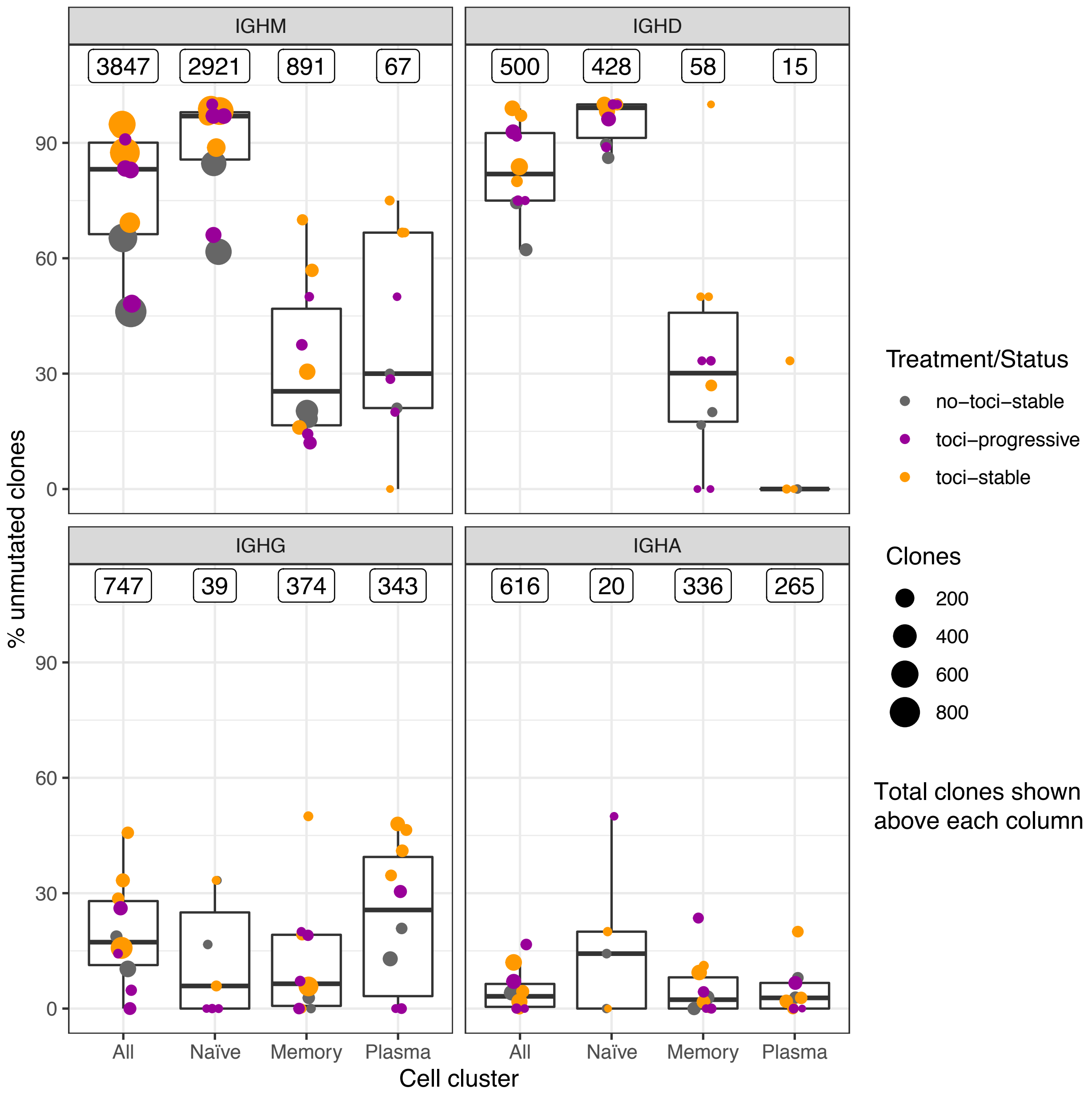

Supplementary Figure 22. **Unmutated B cell clone analysis.**
B cell clones were separated by cell type and isotype. These isotype-specific clonal clusters were considered "unmutated" if the median SHM frequency of their constituent sequences was < 1%. The results are depicted in boxplots, in which the value for each patient is represented by a dot, the upper and lower bounds represent the 75% and 25% percentiles, respectively. The center bars indicate the medians, and the whiskers denote values up to 1.5 interquartile ranges above the 75% or below the 25% percentiles.

Supplementary Tables

	Controls	COVID19 patients			Difference***
		All	Progressive*	Stable**	
Number of subjects	13	10	4	6	
Age (y)	71	71	73.8	69.2	NS; NS
Male Sex	77%	70%	75%	67%	NS; NS
White race	100%	60%	50%	67%	p=0.012; NS
Chronic lung disease	0%	10%	25%	0%	NS; NS
Chronic heart disease	n/a	60%	50%	75%	NS
Chronic hypertension	n/a	90%	100%	83%	NS
BMI	n/a	32.2	34.1	31.0	NS
Hemoglobin A1C	n/a	7.3%	7.1%	7.6%	NS
Symptoms before hospitalization (d)	-	5.5	5.8	5.3	NS
Time from hospitalization to blood sample before tocilizumab (d)	-	2.9	3*	2.8	NS
Time from hospitalization to blood sample after tocilizumab (d)	-	10.1	9.3	11**	NS
Time between samples (d)	-	6.3	5.5*	6.5	NS
Time from hospitalization to tocilizumab treatment (d)	-	4.3	3.3	5.3	NS
Tocilizumab dose (mg/kg)	-	7.8	7.8	7.8	NS
Modified SOFA score (time A)	-	3.3	8.5	1.5	p=0.001
Modified SOFA score (time B)	-	5.8	12.3	1.5	p=0.006
Outcome: discharged	-	60%	0%	100%	p=0.002
Outcome: expired	-	40%	100%	0%	

Supplementary Table 1
Baseline characteristics, timing parameters and outcome for COVID-19 and control subjects.
Values represent means, except for proportions which are presented as %.
P-values calculated with Chi-Square test for proportions and two-tailed T-test for other values.
* Blood before Tocilizumab available only for two progressive patients.
** Two stable patients didn't receive Tocilizumab.
*** P-values are given for (i) Control vs all COVID-19, and (ii) Progressive vs Stable, respectively.
Abbreviations: NS- non significant, n/a- not available.

Yale IMPACT Research Team

Abeer Obaid, Adam J. Moore, Alice Lu-Culligan, Allison Nelson, Amyn A. Malik, Anderson Brito, Angela Nunez, Anjelica Martin, Annie Watkins, Annsea Park, Arvind Venkataraman, Benjamin Goldman-Israelow, Bertie Geng, Camila Odio, Carolina Lucas, Chaney Kalinich, Christina Harden, Codruta Todeasa, Cole Jensen, Daniel Kim, David McDonald, Denise Shepard, Edward Courchaine, Elizabeth B. White, Erin Silva, Eric Song, Eric Y Wang, Eriko Kudo, Erin Silva, Feimei Liu, Harold Rahming, Hong-Jai Park, Irene Matos, Isabel M. Ott, Jessica Nouws, Ji Eun Oh, Jon Klein, Jordan Valdez, Joseph Fauver, Joseph Lim, Julio Silva, Kadi-Ann Rose, Kelly Anastasio, Kristina Brower, Laura Glick, Lina Vadlamani, Lorenzo Sewanan, Lynda Knaggs, M. Catherine Muenker, Marcella Nunez-Smith, Maria Batsu, Maria Tokuyama, Mary Petrone, Maura Nakahata, Maxine Kuang, Melissa Campbell, Melissa Linehan, Michael H. Askenase, Mikhail Smolgovsky, Molly L. Bucklin, Nicole Sonnert, Nida Naushad, Pavithra Vijayakumar, Peiwen Lu, Rebecca Earnest, Rupak Datta, Ryan Handoko, Saad Omer, Sarah Lapidus, Sarah Prophet, Sean Bickerton, Sofia Velazquez, Staci Cahill, Tara Alpert, Tianyang Mao, Tyler Rice, William Khoury-Hanold, Xiaohua Peng, Yexin Yang, Yiyun Cao, Yvette Strong.
