# Supplementary material for: Single-cell multi-omics reveals dyssynchrony of the innate and adaptive immune system in progressive COVID-19
Source: Nat Commun. 2022 Jan 21;13:440. doi: 10.1038/s41467-021-27716-4 (PMC8782894; doi:10.1038/s41467-021-27716-4)
Supplement: Supplementary file 2 — Description of Additional Supplementary Files [file 41467_2021_27716_MOESM2_ESM.pdf]

Description of Additional Supplementary Files

File Name: Supplementary Data 1-10

Description: This file contains the following 10 data sheets:

Supplementary Data 1: Differentially expressed genes (DEGs) in monocytes of progressive vs stable COVID-19 patients

Supplementary Data 2: DEGs in $CD4^+$ T cells of progressive vs stable COVID-19 patients

Supplementary Data 3: DEGs in $CD8^+$ T cells of progressive vs stable COVID-19 patients

Supplementary Data 4: DEGs in B cells of progressive vs stable COVID-19 patients

Supplementary Data 5: TotalSeq-C human panel for CITE-seq

Supplementary Data 6: CITE-seq de-hashing statistics and barcodes

Supplementary Data 7: CITE-seq panel for annotation

Supplementary Data 8: Gene Set Enrichment Analysis (GSEA) custom gene sets

Supplementary Data 9: Ligand-receptor pairs for the connectome analysis

Supplementary Data 10: P values for gene expression comparisons presented in the figure panels
