# Supplementary material for: Single-cell multi-omics reveals dyssynchrony of the innate and adaptive immune system in progressive COVID-19
Source: Nat Commun. 2022 Jan 21;13:440. doi: 10.1038/s41467-021-27716-4 (PMC8782894; doi:10.1038/s41467-021-27716-4)
Supplement: Supplementary file 4 — Reporting Summary [file 41467_2021_27716_MOESM4_ESM.pdf]

nature research

Corresponding author(s): Avraham Unterman, Tomokazu S. Sumida, Charles S. Dela Cruz

Last updated by author(s): Nov 4, 2021



# Reporting Summary

Nature Research wishes to improve the reproducibility of the work that we publish. This form provides structure for consistency and transparency in reporting. For further information on Nature Research policies, see our Editorial Policies and the Editorial Policy Checklist.

## Statistics

For all statistical analyses, confirm that the following items are present in the figure legend, table legend, main text, or Methods section.

| n/a | Confirmed | |
|---|---|---|
| ☐ | ☒ | The exact sample size ($n$) for each experimental group/condition, given as a discrete number and unit of measurement |
| ☐ | ☒ | A statement on whether measurements were taken from distinct samples or whether the same sample was measured repeatedly |
| ☐ | ☒ | The statistical test(s) used AND whether they are one- or two-sided<br>*Only common tests should be described solely by name; describe more complex techniques in the Methods section.* |
| ☐ | ☒ | A description of all covariates tested |
| ☐ | ☒ | A description of any assumptions or corrections, such as tests of normality and adjustment for multiple comparisons |
| ☐ | ☒ | A full description of the statistical parameters including central tendency (e.g. means) or other basic estimates (e.g. regression coefficient) AND variation (e.g. standard deviation) or associated estimates of uncertainty (e.g. confidence intervals) |
| ☐ | ☒ | For null hypothesis testing, the test statistic (e.g. $F$, $t$, $r$) with confidence intervals, effect sizes, degrees of freedom and $P$ value noted<br>*Give P values as exact values whenever suitable.* |
| ☒ | ☐ | For Bayesian analysis, information on the choice of priors and Markov chain Monte Carlo settings |
| ☒ | ☐ | For hierarchical and complex designs, identification of the appropriate level for tests and full reporting of outcomes |
| ☐ | ☒ | Estimates of effect sizes (e.g. Cohen's $d$, Pearson's $r$), indicating how they were calculated |

*Our web collection on statistics for biologists contains articles on many of the points above.*

## Software and code

Policy information about availability of computer code

| | |
|---|---|
| Data collection | Cell suspensions were immediately loaded onto the 10x Chromium Next GEM Chip G, according to the manufacturer's user guide (document number CG000208, revision E, February 2020).<br>Libraries were sequenced on an Illumina Novaseq 6000 platform. |
| Data analysis | Raw sequencing reads were demultiplexed using Cell Ranger mkfastq pipeline to create FASTQ files. Next, Cell Ranger count pipeline (v3.1) was employed in order to perform alignment (using STAR), filtering, barcode counting, and UMI counting. We have used GRCh38 (Ensembl 93) as the genome reference (corresponding to Cell Ranger reference GRCh38-3.0.0).<br>Seurat package3, 4 (v3.1) was used for downstream gene expression analyses. 10x gene expression matrices for each sample were converted and combined into one Seurat object. Cells with mitochondrial gene percentages higher than 12% and cells with less than 200 genes were excluded from the study to filter out dead and dying cells.<br>For CITE-seq samples, following de-hashing, cell barcodes of multiplets (i.e. with 2 or more hashing antibody signals) or uncertain origin (i.e with no clear hashing signal) were also removed.<br>Connectome (https://github.com/msraredon/Connectome) was used for connectomic analysis.<br>V(D)J genes were re-assigned and aligned from CellRanger output using IgBLAST v.1.15.0, IMGT/GENE-DB v3.1.26, and Change-O Python package v.1.0.0 with Python v3.6. GLIPH2 (http://50.255.35.37:8080) was used for clone clustering. R v.3.6.3 was used for downstream analysis with packages: alakazam 1.0.0, airr 1.3.0, iNEXT 2.0.20, dplyr 1.0.0, ggplot2 3.3.0, ggseqlogo 0.1, ggrepel 0.8.1, gridExtra 2.3., msa 1.18.0, Biostrings 2.54.0, ggtree 2.0.4, broom 0.7.0, factoextra 1.0.7, plyr 1.8.4, alakazam 1.0.0, shazam 1.0.0, ggplot2 3.3.2, and purrr 0.3.4. |

For manuscripts utilizing custom algorithms or software that are central to the research but not yet described in published literature, software must be made available to editors and reviewers. We strongly encourage code deposition in a community repository (e.g. GitHub). See the Nature Research guidelines for submitting code & software for further information.

## Data

Policy information about availability of data

All manuscripts must include a data availability statement. This statement should provide the following information, where applicable:

- Accession codes, unique identifiers, or web links for publicly available datasets
- A list of figures that have associated raw data
- A description of any restrictions on data availability

Our data have been deposited in the GEO database under accession code GSE155224. The results can be further explored through the COVID-19 Cell Atlas Data Mining Site (www.covidcellatlas.com). This user-friendly site has a graphical user interface for quick visualization of our scRNA-seq data, which allows users to 1) explore the expression levels of single genes or gene sets of interest across all cell types and 2) conduct comparisons of COVID-19 vs controls, progressive vs stable patients, and early vs late time points across all immune cells in our dataset. Source data are provided with this paper.

# Field-specific reporting

Please select the one below that is the best fit for your research. If you are not sure, read the appropriate sections before making your selection.

- [x] Life sciences
- [ ] Behavioural & social sciences
- [ ] Ecological, evolutionary & environmental sciences

For a reference copy of the document with all sections, see nature.com/documents/nr-reporting-summary-flat.pdf

# Life sciences study design

All studies must disclose on these points even when the disclosure is negative.

| | |
|---|---|
| Sample size | 10 COVID-19 patients and 13 matched healthy controls were included in this study. All patients were confirmed to have COVID-19 by RT-PCR testing of nasopharyngeal samples. 18 blood samples were collected from these 10 patients, at different time-points as described in the Methods and Results section. 13 healthy control subjects were recruited prior to the COVID-19 pandemic. Sample size was not pre-determined; available samples at the time of initiation of study were processed for sequencing. |
| Data exclusions | One out of 18 CITE-seq samples (TP8B) was not pooled because of too low cell concentration. |
| Replication | Due to limited cell number from patients sample, there was no replication of scRNA-seq experiment. Instead, we validated scRNA-seq results by flow cytometry. |
| Randomization | This was an observational study, which did not involve any intervention beyond the standard of care ongoing during the study period. Therefore, randomization is not relevant to this study. |
| Blinding | Blinding for samples from COVID-19 patients vs control was not done as the samples were processed at different period. At the time of patient sample acquisition and processing, patients' conditions were blinded. Blood acquisition was separately performed and recorded by clinical team. Information of patients' conditions was not available until after processing the cells by 10X instrument. Blinding for analysis was not performed because this study assessed changes of immune profiles over time and disease severities. |

# Reporting for specific materials, systems and methods

We require information from authors about some types of materials, experimental systems and methods used in many studies. Here, indicate whether each material, system or method listed is relevant to your study. If you are not sure if a list item applies to your research, read the appropriate section before selecting a response.

## Materials & experimental systems

| n/a | Involved in the study | |
|---|---|---|
| [ ] | [x] | Antibodies |
| [x] | [ ] | Eukaryotic cell lines |
| [x] | [ ] | Palaeontology and archaeology |
| [x] | [ ] | Animals and other organisms |
| [ ] | [x] | Human research participants |
| [x] | [ ] | Clinical data |
| [x] | [ ] | Dual use research of concern |

## Methods

| n/a | Involved in the study | |
|---|---|---|
| [x] | [ ] | ChIP-seq |
| [ ] | [x] | Flow cytometry |
| [x] | [ ] | MRI-based neuroimaging |

## Antibodies

| | |
|---|---|
| Antibodies used | BB515 anti-hHLA-DR (G46-6) (1:400) (BD Biosciences, Cat#564516),<br>BV605 anti-hCD3 (UCHT1) (1:300) (BioLegend, Cat#300460)<br>BV785 anti-hCD4 (SK3) (1:200) (BioLegend, Cat#344642), |

| | |
|---|---|
| | APCFire750 anti-hCD8 (SK1) (1:200) (BioLegend, Cat#344746),<br>BV421 anti-hCCR7 (G043H7) (1:50) (BioLegend, Cat#353208),<br>AlexaFluor 700 anti-hCD45RA (HI100) (1:200) (BD Biosciences, Cat#560673),<br>BV711 anti-hCD38 (HIT2) (1:200) (BioLegend, Cat#303528),<br>PECy7 anti-hCD127 (HIL-7R-M21) (1:50) (BioLegend, Cat#),<br>PE-CF594 anti-hCD25 (BC96) (1:200) (BD Biosciences, Cat#),<br>BV421 anti-hLAG-3 (11C3C65) (1:200) (Biolegend, Cat# 369314) |
| Validation | All antibodies used in this study are commercially available, and all have been validated by the manufacturers and used by other publications (https://doi.org/10.1038/s41586-020-2588-y).<br>Antibody validations performed by antibody suppliers are followings:<br>https://www.bdbiosciences.com/en-us/products/reagents/flow-cytometry-reagents/research-reagents/single-color-antibodies-ruo/alexa-fluor-700-mouse-anti-human-cd45ra.560673<br>https://www.biolegend.com/en-us/products/brilliant-violet-711-anti-human-cd38-antibody-7936<br>https://www.biolegend.com/en-us/products/brilliant-violet-421-anti-human-cd197-ccr7-antibody-7497<br>https://www.biolegend.com/en-us/products/apc-fire-750-anti-human-cd8-antibody-13035<br>https://www.biolegend.com/en-us/products/brilliant-violet-785-anti-human-cd4-antibody-15650<br>https://www.biolegend.com/en-us/search-results/brilliant-violet-605-anti-human-cd3-antibody-10421?GroupID=BLG5900<br>https://www.bdbiosciences.com/en-us/products/reagents/flow-cytometry-reagents/research-reagents/single-color-antibodies-ruo/bb515-mouse-anti-human-hla-dr.564516<br>https://www.biolegend.com/en-us/products/brilliant-violet-421-anti-human-cd223-lag-3-antibody-13668 |

# Human research participants

Policy information about studies involving human research participants

| | |
|---|---|
| Population characteristics | Baseline characteristics of COVID-19 patients and controls are presented in Supplementary Table 1. |
| Recruitment | Patients admitted to the Yale New Haven Hospital (YNHH) between the 18th of March through the 27th of May 2020, were recruited to the Yale IMPACT study (Implementing Medical and Public Health Action Against Coronavirus CT) after testing positive for SARS-CoV2 by qRT-PCR. Written informed consent was obtained by trained staff and sample collection commenced immediately upon study enrollment. |
| Ethics oversight | Research protocol was reviewed and approved by the Yale Human Research Protection Program Institutional Review Boards (FWA00002571, Protocol ID. 2000027690). |

Note that full information on the approval of the study protocol must also be provided in the manuscript.

# Flow Cytometry

## Plots

Confirm that:

- [x] The axis labels state the marker and fluorochrome used (e.g. CD4-FITC).
- [x] The axis scales are clearly visible. Include numbers along axes only for bottom left plot of group (a 'group' is an analysis of identical markers).
- [x] All plots are contour plots with outliers or pseudocolor plots.
- [x] A numerical value for number of cells or percentage (with statistics) is provided.

## Methodology

| | |
|---|---|
| Sample preparation | Freshly isolated PBMCs were stained for live and dead markers, blocked with Human TruStan FcX , stained for surface markers and then fixed with PFA 4%. For intracellular cytokine staining following stimulation , cells were surface stained, washed and fixed in 4% PFA. After permeabilization with 1X Permeabilization Buffer cells were stained for intracellular cytokines analysis. |
| Instrument | Data was acquired on a BD Fortessa flow cytometer. |
| Software | FlowJo software version 10.6 software (Tree Star). |
| Cell population abundance | Cell populations were reported as a proportion of a parent gate (% of CD3+ T cells). |
| Gating strategy | SSC-A and FSC-A parameters were used to select leukocytes from isolated PBMCs. Live and dead cells were defined based on live dead aqua staining. Singlets were separated based on SSC-H/SSC-W and FSC-H/FSC-W parameters. Total T cells were defined as CD3 positive population. |

- [x] Tick this box to confirm that a figure exemplifying the gating strategy is provided in the Supplementary Information.
